# Integrin **α**9 regulates smooth muscle cell phenotype switching and vascular remodeling

**DOI:** 10.1172/jci.insight.147134

**Published:** 2021-05-24

**Authors:** Manish Jain, Rishabh Dev, Prakash Doddapattar, Shigeyuki Kon, Nirav Dhanesha, Anil K. Chauhan

**Affiliations:** 1 Division of Hematology-Oncology and Blood & Marrow Transplantation, Department of Internal Medicine, Carver College of Medicine, University of Iowa, Iowa City, Iowa, USA.; 2 Department of Molecular Immunology, Faculty of Pharmaceutical Sciences, Fukuyama University, Hiroshima, Japan.

**Keywords:** Vascular Biology, Cell migration/adhesion, Integrins, Mouse models

## Abstract

Excessive proliferation of vascular smooth muscle cells (SMCs) remains a significant cause of in-stent restenosis. Integrins, which are heterodimeric transmembrane receptors, play a crucial role in SMC biology by binding to the extracellular matrix protein with the actin cytoskeleton within the SMC. Integrin α9 plays an important role in cell motility and autoimmune diseases; however, its role in SMC biology and remodeling remains unclear. Herein, we demonstrate that stimulated human coronary SMCs upregulate α9 expression. Targeting α9 in stimulated human coronary SMCs, using anti–integrin α9 antibody, suppresses synthetic phenotype and inhibits SMC proliferation and migration. To provide definitive evidence, we generated an SMC-specific α9-deficient mouse strain. Genetic ablation of α9 in SMCs suppressed synthetic phenotype and reduced proliferation and migration in vitro. Mechanistically, suppressed synthetic phenotype and reduced proliferation were associated with decreased focal adhesion kinase/steroid receptor coactivator signaling and downstream targets, including phosphorylated ERK, p38 MAPK, glycogen synthase kinase 3β, and nuclear β-catenin, with reduced transcriptional activation of β-catenin target genes. Following vascular injury, SMC-specific α9-deficient mice or wild-type mice treated with murine anti–integrin α9 antibody exhibited reduced injury-induced neointimal hyperplasia at day 28 by limiting SMC migration and proliferation. Our findings suggest that integrin α9 regulates SMC biology, suggesting its potential therapeutic application in vascular remodeling.

## Introduction

Percutaneous coronary intervention (PCI) followed by stent implantation currently remains the treatment of choice to treat obstructive coronary arteries. However, the procedure is hampered by in-stent restenosis, a phenomenon characterized by smooth muscle cell (SMC) phenotypic switching, SMC proliferation, and neoatherosclerosis (1). Care of patients with in-stent restenosis remains a prevailing clinical problem. Although drug-eluting stents reduce the incidence of in-stent restenosis, patients are not completely immune to restenosis. Evidence suggests that even with newer generation devices, the restenosis rate is approximately 5%–10% (2). According to the US guidelines, the patients who develop clinical restenosis following drug-eluting stent implantation may again be considered for PCI with balloon angioplasty or drug-eluting stents (3). Therefore, a better understanding of molecular mechanisms that regulate SMC phenotypic switching and proliferation is required to develop effective therapeutic strategies to improve clinical outcomes following PCI.

Integrins are heterodimeric transmembrane receptors responsible for numerous cellular functions, including cell adhesion and migration (4). The integrin family includes 18 α and 8 β subunits that form 24 distinct αβ heterodimers (5). Unlike other integrins that recognize Arg-Gly-Asp (RGD) sequence, α9β1 recognizes a Met-Leu-Asp sequence. Genetic knockout studies in mice revealed that phenotypes do not overlap, suggesting different functions in vivo (6). To date, β1 is the only reported subunit of α9. Besides SMCs, integrin α9β1 is expressed on several other cells, including neutrophils, hepatocytes, endothelial cells, and epithelial cells (7, 8). The potential mechanistic role of α9β1 in phenotypic modulation of SMCs and neointimal hyperplasia has not been investigated yet to our knowledge.

Herein, we determined the mechanistic role of integrin α9 in SMC phenotypic modulation in vitro and neointimal hyperplasia in normolipidemic and comorbid conditions of hyperlipidemia. We chose hyperlipidemic conditions in vivo because of the following reasons: first, patients with hyperlipidemia have a higher risk of developing restenosis following vascular injury (9, 10). PCI procedures are performed in patients who often have elevated levels of cholesterol or triglycerides. Second, hyperlipidemia, a causative factor for neoatherosclerosis, contributes to the progression of in-stent restenosis by potentiating inflammation (11). Herein, utilizing human coronary SMC and SMC-specific integrin α9^–/–^ mice, we demonstrate integrin α9β1 mechanistically regulates different aspects of SMC biology and promotes neointimal hyperplasia following vascular injury.

## Results

### Targeting α9 in stimulated human coronary SMCs suppresses synthetic phenotype and inhibits SMC proliferation and migration.

**Figure 1:**
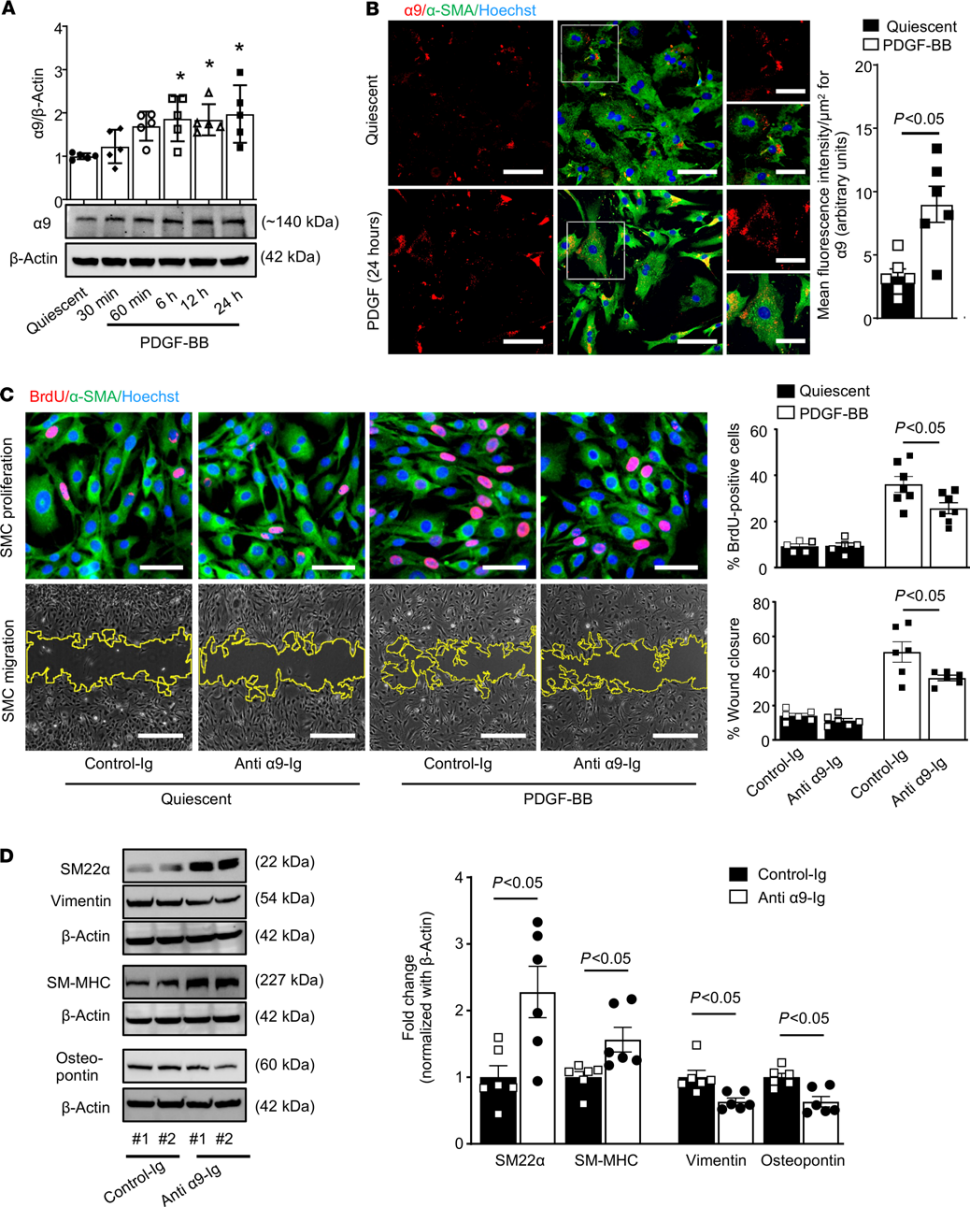
Integrin α9 is upregulated in stimulated human coronary SMCs, and treatment with anti–integrin α9 antibody suppresses synthetic phenotype and inhibits proliferation and migration. (**A**) Serum-starved human coronary SMCs were stimulated with or without PDGF-BB for indicated time points. Representative immunoblots and densitometric analysis of α9 and expression levels. β-Actin was used as a loading control (*n* = 5/group). (**B**) The left panels show representative double immunostaining for α9 (shown in red) and SMC actin (αSMA) (shown in green) in SMCs stimulated with or without PDGF-BB for 24 hours. Boxed regions are magnified (scale bars: 25 μm). Scale bars: 50 μm. The right panel shows the quantification of α9 fluorescence intensity (*n* = 6/group). (**C** and **D**) Quiescent human coronary SMCs were pretreated with anti-α9 blocking antibody (clone Y9A2, 10 μg/mL) for 60 minutes. (**C**) Human coronary SMCs were stimulated with PDGF-BB for 24 hours. The top panels show representative BrdU-positive cells (red) costained with αSMA (green) and Hoechst (blue). Scale bars: 50 μm. The bottom panels show representative phase-contrast images of SMC migration in the scratch assay. Scale bars: 500 μm. The right panel shows the quantification of BrdU-positive cells to the total number of cells (*n* *=* 6/group) and migrated area (*n* *=* 6/group). (**D**) Representative immunoblots and densitometric analysis of SM22α, SM-MHC, vimentin, and osteopontin (*n* = 6/ group) in human coronary SMCs stimulated with PDGF-BB for 24 hours. #1 and #2 represent 2 separate samples. Statistical analysis: (**A**) 1-way ANOVA with Bonferroni’s post hoc test; (**C**) 2-way ANOVA followed by uncorrected Fisher’s least significant differences (LSD) test (**B** and **D**) unpaired 2-tailed Student’s *t* test. *P < 0.05 vs. quiescent or vehicle-treated (control Ig) groups.

To evaluate the potential role of α9β1 in SMC biology, we first assessed the time-dependent effect of platelet-derived growth factor–BB (PDGF-BB), which is known to modulate the membrane mobility and trafficking of integrins (12), on α9 expression. Immunoblot analysis suggested an approximately 2-fold increase in integrin α9 expression levels in PDGF-BB–stimulated human coronary SMCs after 6–24 hours (*P* < 0.05, Figure 1A). These results were confirmed in parallel by immunofluorescence (*P* < 0.05, Figure 1B). Next, we determined the effect of human-specific anti–integrin α9 antibody (clone Y9A2) on SMC proliferation and migration. Serum-deprived human coronary SMCs were preincubated with Y9A2 (10 μg/mL) for 1 hour and then stimulated with PDGF-BB. SMC morphology was normal during and after treatment. A significant decrease in human coronary SMC proliferation was observed in the group treated with anti–integrin α9 Ig compared with the Ig-treated control (36.11 ± 3.4% vs. 25.8 ± 2.3 %; Figure 1C). In a scratch wound healing assay, we found that anti–integrin α9 Ig significantly decreased PDGF-BB–induced human coronary SMC migration (51.9 ± 14.2% vs. 37.2 ± 1.5%; Figure 1C). Anti–integrin α9 Ig treatment did not exert any significant effect on SMC proliferation or migration in quiescent SMCs. Upon activation, it is known that an SMC undergoes phenotypic modulation from a differentiated “contractile” to a dedifferentiated “synthetic” proliferative phenotype, a process that contributes to proliferation and migration. To determine the role of integrin α9 in SMC phenotypic switching, anti–integrin α9 Ig–treated or Ig-treated human coronary SMCs were stimulated with PDGF-BB and immunostained for contractile (smooth muscle myosin heavy chain 11, SM-MHC; and smooth muscle 22α, SM22α) and synthetic (vimentin and osteopontin) phenotype markers (13). We found that the protein levels of contractile markers were higher, whereas synthetic markers were lower in anti–integrin α9 Ig–treated SMCs than Ig-treated cells (*P* < 0.05, Figure 1D). Together, these results suggest that blocking integrin α9 signaling with anti–integrin α9 antibody inhibits SMC proliferation and migration and suppresses synthetic phenotype.

### Integrin α9 regulates SMC phenotypic switching, proliferation, and migration via focal adhesion kinase/steroid receptor coactivator signaling pathway.

**Figure 2:**
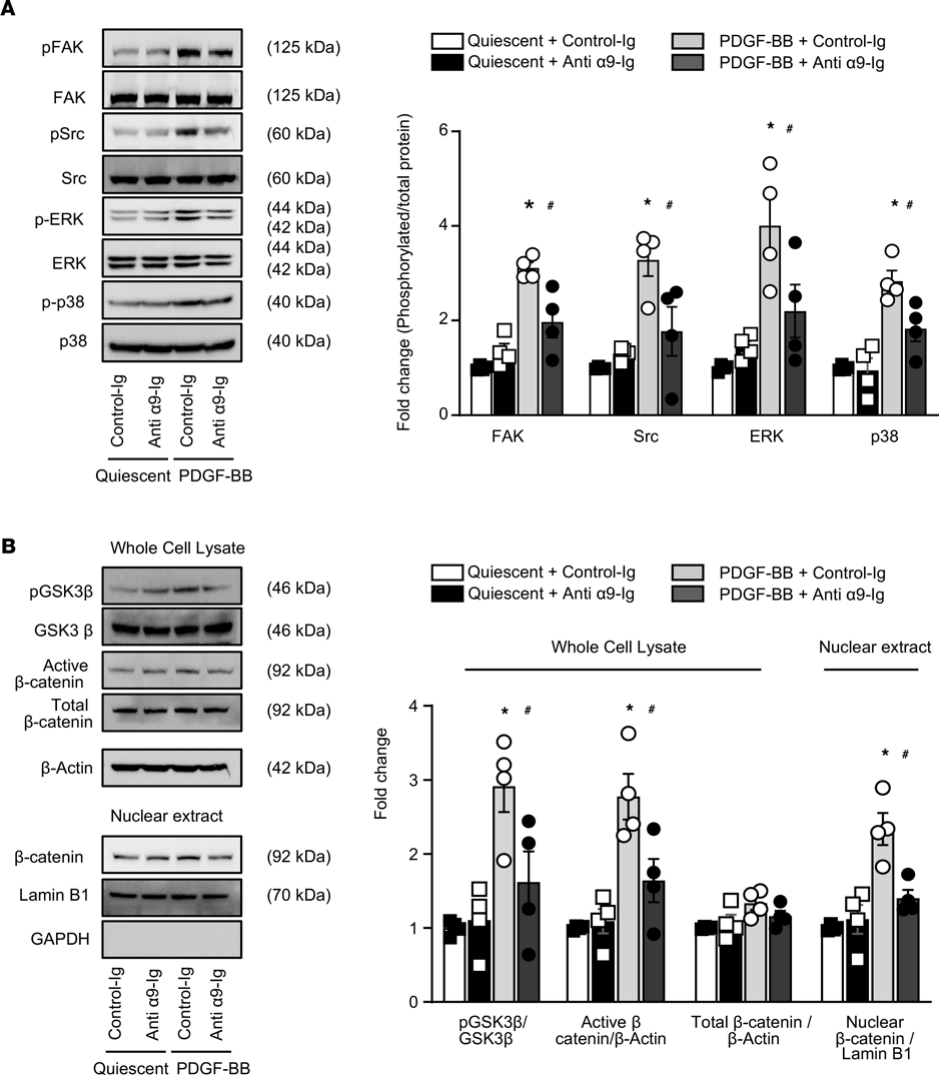
Anti–integrin α9 antibody suppresses FAK/Src, ERK, p38, and glycogen synthase kinase 3β/β-catenin pathway. Serum-starved human coronary SMCs were stimulated with or without PDGF-BB. (**A**) Representative Western blots and densitometric analysis of FAK, Src, ERK1/2, p38, and β-actin after 30 minutes of PDGF-BB stimulation (*n* = 4/group). (**B**) Representative Western blots and densitometric analysis of GSK3β, β-catenin, and β-actin after 30 minutes of PDGF-BB stimulation. Nuclear extracts were prepared after 6 hours of PDGF-BB stimulation. β-Catenin and Lamin B1 were detected by immunoblotting (*n* = 4/group). Statistical analysis: 2-way ANOVA followed by uncorrected Fisher’s LSD test. *P < 0.05 vs. quiescent + control Ig; ^#^*P* < 0.05 vs. PDGF-BB + control Ig–treated groups. p-ERK, phosphorylated ERK.

Integrins are known to activate signaling pathways, including the activation of focal adhesion kinase (FAK), steroid receptor coactivator (Src), ERK, and p38 MAPK (4, 14). We determined whether integrin α9 regulates the FAK/Src pathway, which is known to contribute to cell motility and is a critical signaling event associated with ligand binding to integrins. Quiescent human coronary SMCs pretreated with anti–integrin α9 Ig or control Ig were stimulated with PDGF-BB for 30 minutes. Using immunoblotting, we found that pretreatment with anti–integrin α9 Ig significantly reduced PDGF-BB–induced phosphorylation of FAK (~0.6-fold) and Src (~0.6-fold) when compared with control Ig–treated cells (Figure 2A). Furthermore, anti–integrin α9 Ig treatment suppressed PDGF-BB–induced activation of ERK1/2 (0.5-fold) and p38 MAPK pathway (0.5-fold) that are linked to cell proliferation and inflammation (Figure 2A). Following integrin engagement, glycogen synthase kinase 3β (GSK3β) phosphorylates β-catenin, the canonical Wnt pathway’s signaling molecule, making it more prone to proteasomal degradation (15). We evaluated whether blocking α9 signaling affects GSK3β and β-catenin expression. We found that anti–integrin α9 Ig attenuated GSK3β phosphorylation (0.6-fold) that was associated with reduced β-catenin expression (0.4-fold) when compared with control (Figure 2B). On the other hand, total β-catenin levels were comparable after 30 minutes of PDGF-BB stimulation. It is known that β-catenin translocates to the nucleus after 6 hours of PDGF-BB stimulation (16). We found that nuclear β-catenin expression, after 6 hours of PDGF-BB stimulation, was significantly reduced in SMCs treated with anti–integrin α9 Ig (0.6-fold) when compared with Ig-treated SMCs (Figure 2B). Anti–integrin α9 Ig treatment did not exert any significant effect on FAK/Src signaling and its downstream targets in quiescent SMCs. Together these results suggest that integrin α9 mechanistically regulates SMC proliferation, migration, and synthetic phenotype via FAK/Src signaling and its downstream targets, including p-ERK, p38 MAPK, and p-GSK3β/β-catenin signaling.

### SMC-derived integrin α9 mediates neointimal hyperplasia in mice.

**Figure 3:**
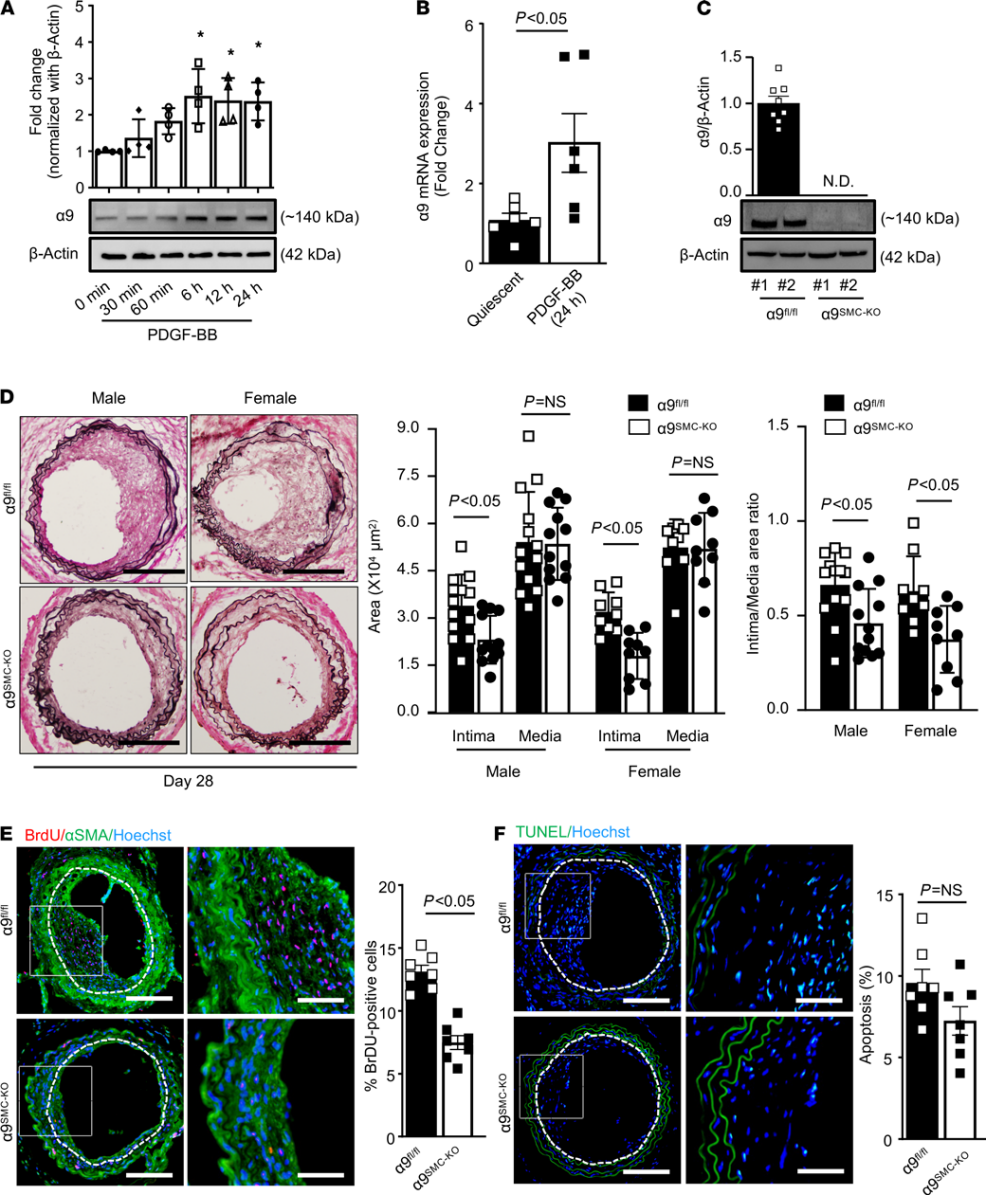
SMC-specific integrin α9 modulates neointimal hyperplasia in mice. All mice are on Apoe^–/–^ background. Serum-deprived SMCs were stimulated with or without PDGF-BB (20 ng/mL) for indicated time points. (**A**) Representative immunoblots and densitometric analysis of α9 expression levels. β-Actin was used as a loading control (*n* = 4). (**B**) Quantification of α9 mRNA expression by real-time PCR (*n* = 6). (**C**) Western blot analysis of α9 and β-actin in SMCs isolated from α9^SMC-KO^ and control α9^fl/fl^ mice. #1 and #2 represent samples from 2 individual mice. (**D**) The left panels show representative photomicrographs of Verhoeff’s van Gieson–stained carotid artery sections from male and female α9^SMC-KO^ and control α9^fl/fl^ mice after 28 days of injury (male *n* = 12–13; female *n* = 9/group). Scale bars: 200 μm. The right panels show quantification of intimal area, medial area, and a ratio of intimal to medial area. Each dot represents a single mouse. (**E**) The left panels show representative BrdU-positive cells (red) counterstained with αSMA (green). Nuclei are counterstained with Hoechst (blue). The right panel shows the quantification of percentage BrdU-positive cells (*n* = 7). Scale bars: 200 μm. (**F**) The left panels show representative TUNEL-positive cells (green) counterstained with Hoechst (blue). The right panel shows the quantification of TUNEL-positive cells (*n* = 7). Scale bars: 200 μm. Values are represented as mean ± SEM. Statistical analysis: (**A**) 1-way ANOVA followed by Bonferroni’s post hoc test; (**B**–**F**) unpaired 2-tailed Student’s *t* test. *P < 0.05 versus quiescent. N.D., not detected.

We first evaluated the time-dependent effect of PDGF-BB on α9 expression in murine aortic SMCs. Similar to human coronary SMCs, PDGF-BB stimulation led to a time-dependent increase in α9 expression in murine aortic SMCs (*P* < 0.05, Figure 3A). In line with these results, α9 mRNA expression levels were increased following PDGF-BB stimulation compared with control quiescent SMCs (*P* < 0.05, Figure 3B). In parallel, we found that α9 expression was higher in the injured carotid artery at 28 days when compared with the uninjured control artery (Supplemental Figure 1; supplemental material available online with this article; https://doi.org/10.1172/jci.insight.147134DS1). β1 is the only reported subunit of α9; therefore, genetic deletion of α9 should inhibit α9β1-mediated signaling only. To show a definitive role for α9β1 in SMC biology and neointimal hyperplasia in the comorbid condition of hyperlipidemia, we generated SMC-specific α9-deficient mice on apolipoprotein E–deficient (Apoe^–/–^) background (α9^fl/fl^ SM22αCre^+^ Apoe^–/–^; hereafter for simplicity referred to as α9^SMC-KO^; Supplemental Figure 2A). The SM22αCre^+^ transgenic mice expressed Cre recombinase under the control of mouse SMC protein 22α (or transgelin) promoter. Genomic PCR confirmed the presence of the SM22αCre gene in α9^fl/fl^ Apoe^–/–^ mice (Supplemental Figure 2B). Previously, it was shown that the global deletion of α9 in mice results in a defect in granulopoiesis (17). In contrast, we found that complete blood counts were comparable between α9^SMC-KO^ and control α9^fl/fl^ mice (Supplemental Table 1), suggesting that SMC-specific deletion of α9 does not result in impaired granulopoiesis and that α9^SMC-KO^ could be used as an appropriate model to evaluate the role of integrin α9 in SMC biology. Western blot analysis confirmed the lack of integrin α9 expression in SMCs isolated from the α9^SMC-KO^ mice (Figure 3C). Genetic deletion of integrin α9 in SMCs did not affect β1 subunit expression (Supplemental Figure 3). Susceptibility to neointimal hyperplasia was studied at 28 days following wire injury in the carotid artery of littermate α9^fl/fl^ and α9^SMC-KO^ mice. Male and female mice were used to determine sex-based differences. SMC-specific integrin α9 deletion led to a significant decrease in neointimal area and neointima/media ratio (*P* < 0.05 versus α9^fl/fl^; Figure 3D). The medial area was comparable between α9^fl/fl^ and α9^SMC-KO^ mice (Figure 3D). Immunostaining revealed that α9 expression was minimal and mainly restricted to the luminal area in α9^SMC-KO^ mice (Supplemental Figure 4). Next, we analyzed SMC proliferation and apoptosis in wire-injured carotid artery sections because of the differential role in neointima formation. We found that SMC proliferation was significantly reduced in α9^SMC-KO^ mice when compared with control α9^fl/fl^ mice (Figure 3E). Next, we analyzed BrdU incorporation in the neointima and the media. We found that percentage of BrdU-positive cells was significantly reduced in the neointima but not in the media of α9^SMC-KO^ mice when compared with control α9^fl/fl^ mice (Supplemental Figure 5). SMC-specific α9 deletion did not affect apoptosis (Figure 3F). Together these results suggest a mechanistic role for SMC-derived integrin α9 in vascular remodeling.

### SMC-specific integrin α9 regulates SMC phenotypic switching, proliferation, and migration via FAK/Src, ERK, p38, and GSK3β/β-catenin pathway.

**Figure 4:**
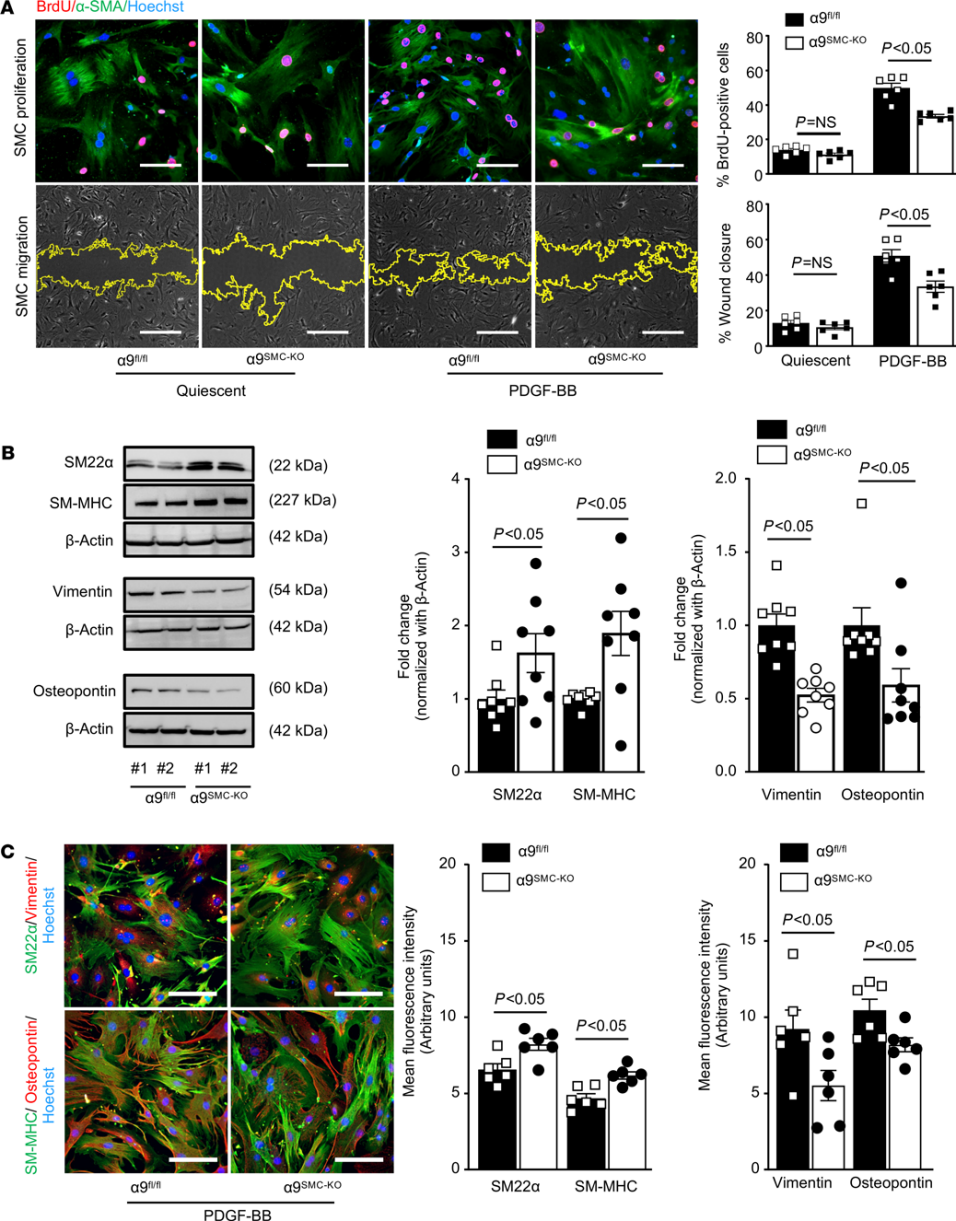
SMC-specific α9 deletion suppresses PDGF-BB–induced SMC proliferation, migration, and phenotypic switching. Aortic SMCs isolated from α9^SMC-KO^ and control α9^fl/fl^ mice were serum-starved and stimulated with PDGF-BB for 24 hours. (**A**) The left upper panels show representative BrdU-positive cells (red) costained with αSMA (green) and Hoechst (blue). Scale bars: 50 μm. The left lower panels show representative phase-contrast images of SMC migration in the scratch assay. Scale bars: 500 μm. The right panel shows the quantification of BrdU-positive cells to the total number of cells and quantification of the migrated area (*n* = 6). (**B**) Representative immunoblots and densitometric analysis of contractile proteins (SM22α and SM-MHC) and synthetic proteins (vimentin and osteopontin) (*n* = 8). (**C**) The left panels show representative immunostained images for contractile proteins (SM22α, green; and SM-MHC, green) and synthetic proteins (vimentin, red; and osteopontin, red). Scale bars: 50 μm. The right panels show quantification of the fluorescence intensity for SM22α, SM-MHC, vimentin, and osteopontin (*n* = 6 per group). Values are expressed as mean ± SEM. Statistical analysis: (**A**) 2-way ANOVA followed by uncorrected Fisher’s LSD test; (**B** and **C**) unpaired 2-tailed Student’s *t* test.

Cultured SMCs from α9^fl/fl^ and α9^SMC-KO^ mice were subjected to BrdU incorporation and scratch wound assays to obtain further mechanistic insights. We found that SMC-specific α9 deletion led to a significant decrease in PDGF-BB–induced SMC proliferation (49.8 % ± 2.8 % vs. 33.5% ± 1.0 %; Figure 4A) and SMC migration (50.8 % ± 3.5 % vs. 33.5 % ± 3.1 %; Figure 4A) when compared with control. To elucidate the role of integrin α9 on SMC phenotypic switching, PDGF-BB–stimulated SMCs from α9^fl/fl^ and α9^SMC-KO^ mice were subjected to Western blotting assay. We found that the expression levels of contractile markers (SM22α and SM-MHC) were higher than control. In contrast, synthetic markers (vimentin and osteopontin) were lower in SMCs isolated from α9^SMC-KO^ mice when compared with SMCs from α9^fl/fl^ mice (Figure 4B). These results were confirmed in parallel by immunofluorescence (Figure 4C). Together, these results suggest that genetic ablation of integrin α9 prevents SMC proliferation, migration, and phenotypic switching.

**Figure 5:**
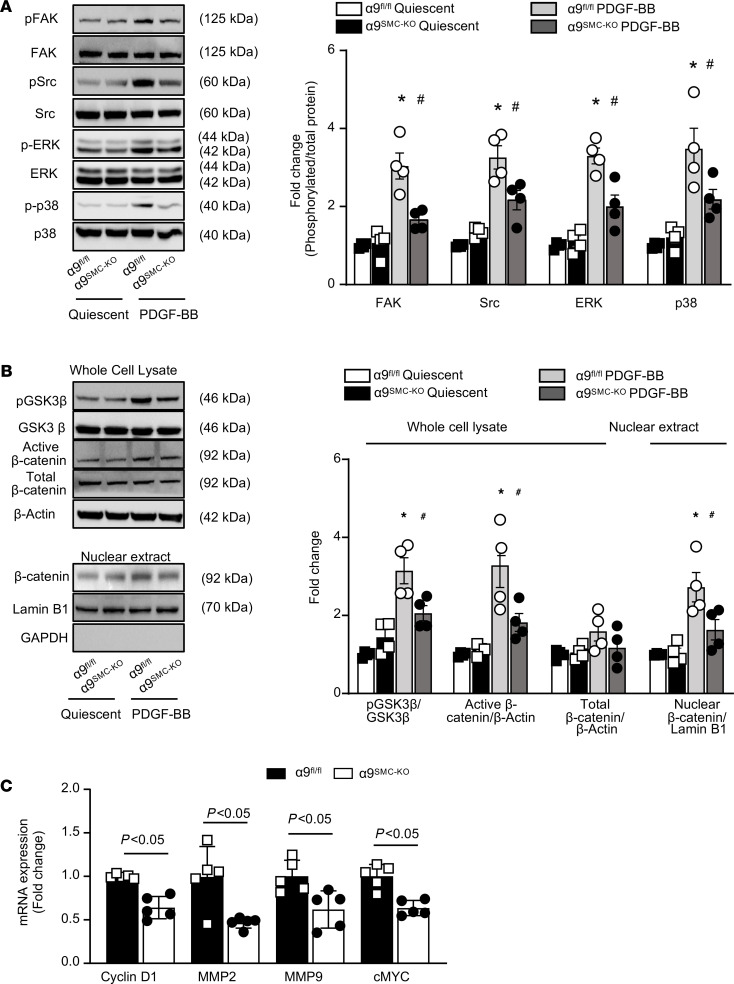
SMC-specific α9 deletion suppresses FAK, Src, ERK, p38, and GSK3β/β-catenin pathway. Aortic SMCs isolated from α9^SMC-KO^ and control α9^fl/fl^ mice were serum-starved for 48 hours. (**A**) Representative Western blots and densitometric analysis of FAK, Src, ERK1/2, p38, and β-actin after 30 minutes of PDGF-BB stimulation (*n* = 4). (**B**) Representative Western blots and densitometric analysis of GSK3β, β-catenin, and β-actin after 30 minutes of PDGF-BB stimulation (*n* = 4). Nuclear extracts were prepared after 6 hours of PDGF-BB stimulation. β-Catenin and Lamin B1 were detected by immunoblotting (*n* = 4). (**C**) Real-time quantitative PCR analysis of cyclin D1, MMP-2, MMP-9, and c-Myc (*n* = 5) in SMCs stimulated with PDGF-BB (20 ng/mL) for 24 hours. Values are expressed as mean ± SEM. Statistical analysis: (**A** and **B**) 2-way ANOVA followed by uncorrected Fisher’s LSD test. *P < 0.05 versus α9^fl/fl^ quiescent SMCs, ^#^*P* < 0.05 vs α9^fl/fl^ PDGF-BB–treated SMCs. (**C**) Unpaired 2-tailed Student’s *t* test.

We next determined whether SMC-specific deletion of integrin α9 limits PDGF-BB–induced activation of FAK, Src, ERK, p38, and GSK3β/β-catenin signaling, similar to that observed in human coronary SMCs treated with anti–integrin α9 Ig. Using immunoblotting, we found that after 30 minutes of PDGF-BB stimulation, aortic SMCs from α9^SMC-KO^ mice exhibited reduced p-FAK (~0.6 fold) and p-SRC (~0.4 fold) when compared with control α9^fl/fl^ mice (Figure 5A). Furthermore, deletion of α9 suppressed PDGF-BB–induced p-ERK1/2 (0.5-fold) and p-p38 expression (0.4-fold) (Figure 5A). We also found that deletion of α9 attenuated GSK3β phosphorylation (0.6-fold). Concomitantly a decrease in nonphosphorylated β-catenin expression (0.5-fold) was noted in α9-deficient SMCs compared with control (Figure 5B). No significant change in total β-catenin expression was observed in α9-deficient SMCs when compared to control. However, nuclear β-catenin expression was significantly reduced in SMCs from α9^SMC-KO^ mice compared with control (0.6-fold, Figure 5B). We further explored how α9 deletion downregulates β-catenin expression. SMCs were stimulated with PDGF-BB in the presence or absence of proteasome-specific inhibitor (PSI, 10 μM). Expression of β-catenin protein was monitored in the nuclear extract after 6 hours of PDGF-BB stimulation. We found that PSI treatment significantly recovered β-catenin protein level in α9-deficient SMCs, suggesting that integrin α9 promotes β-catenin degradation through proteasome pathway (Supplemental Figure 6). Several genes are regulated by the Wnt/β-catenin pathway, including matrix metalloproteinase 2 (MMP-2), MMP-9 (18), cyclin D1 (19), and c-Myc (20), which are known to contribute to SMC migration and proliferation. We found that SMC-specific integrin α9 deletion reduced PDGF-BB–induced transcription of MMP-2, MMP-9, cyclin D1, and c-Myc (Figure 5C).

### Extracellular matrix protein fibronectin containing extra domain A partially contributes to integrin α9–mediated SMC proliferation, migration, and phenotypic switching.

**Figure 6:**
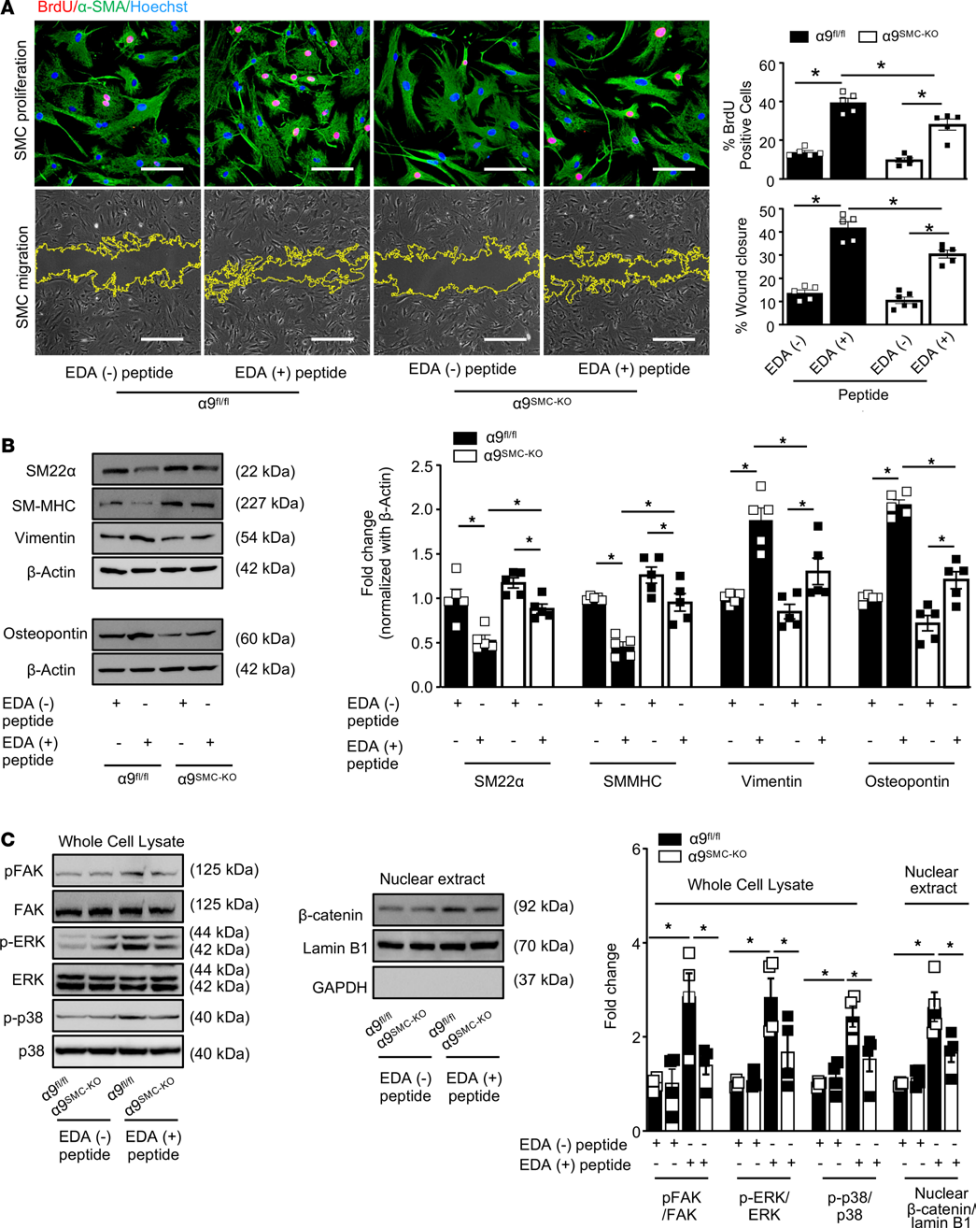
Fn-EDA partially contributes to α9-mediated SMC proliferation and migration. Quiescent SMCs were exposed to 10 μg/mL of recombinant peptides containing or lacking EDA. (**A**) The upper left panels show representative images of BrdU-positive cells (red) costained with αSMA (green) and Hoechst (blue) analyzed 24 hours after EDA peptide treatment. Scale bar: 50 μm. Lower left panels show representative phase-contrast images of SMC migration in the scratch assay analyzed 24 hours after EDA peptide treatment. Scale bar: 500 μm. The right panels show quantification of BrdU-positive cells (*n* *=* 5) and migrated area (*n* *=* 5). (**B**) Cells were processed for Western blotting after 24 hours of EDA peptide treatment. Representative immunoblots and densitometric analysis of SM22α, SM-MHC, vimentin, osteopontin, and β-actin (*n* *=* 5). (**C**) Representative immunoblots and densitometric analysis of FAK, Src, ERK1/2, p38, GSK3β, β-catenin, and β-actin after 30 minutes of PDGF-BB stimulation (*n* = 4/group). Nuclear extracts were prepared after 6 hours of PDGF-BB stimulation. β-Catenin and Lamin B1 were detected by immunoblotting (*n* = 4/group). Values are expressed as mean ± SEM. Statistical analysis: 2-way ANOVA followed by Fisher’s LSD test. *P < 0.05.

The extracellular matrix protein fibronectin containing extra domain A (Fn-EDA) is an endogenous ligand for integrin α9β1 (21). To determine whether SMC-specific integrin α9 mediates SMC proliferation and migration via Fn-EDA, we treated SMCs from α9^fl/fl^ and α9^SMC-KO^ mice with recombinant EDA-containing [EDA (+)] or EDA-lacking [EDA (-)] peptides (10 μg/mL). We found that EDA (+) peptide significantly increased proliferation and migration of SMCs isolated from α9^fl/fl^ mice (Figure 6A). Furthermore, EDA (+) peptide increased the expression levels of synthetic markers (vimentin and osteopontin) and decreased the expression of contractile markers (SM22α and SM-MHC) in SMCs of α9^fl/fl^ mice (Figure 6B). EDA (+) peptide also increased proliferation and migration of SMCs of α9^SMC-KO^ mice, suggesting a role of other EDA ligands in addition to α9β1 in SMC proliferation and migration. It is important to note that the extent of proliferation and migration was significantly reduced in SMCs of α9^SMC-KO^ mice treated with EDA (+) peptide compared with SMCs of α9^fl/fl^ mice treated with EDA (+) peptide (Figure 6A), suggesting Fn-EDA partially contributes to integrin α9–mediated SMC proliferation and migration. Similarly, EDA (+) peptide increased the expression of synthetic markers (vimentin and osteopontin) and decreased the expression of contractile markers (SM22α and SM-MHC) in SMCs of α9^SMC-KO^ mice. However, the fold change was significantly less than the SMCs of α9^fl/fl^ mice treated with EDA (+) peptide (Figure 6B). Previous reports suggest that fibronectin-EDA interaction with integrin α9 results in activation of FAK, ERK, p38, and β-catenin signaling pathways (22). We determined activation of these proteins in α9-deficient SMCs treated with recombinant EDA-containing or -lacking peptide. Using immunoblotting, we found that EDA (+) peptide significantly increased the expression of p-FAK, p-ERK1/2, and p-p38 in SMCs from control α9^fl/fl^ mice (Figure 6C). In line with these observations, EDA (+) peptide treatment significantly increased nuclear β-catenin expression in SMCs from α9^fl/fl^ mice. On the other hand, genetic deletion of α9 partially inhibited EDA (+) peptide–induced increased expression of p-FAK, p-ERK1/2, p-p38, and nuclear β-catenin expression (Figure 6C). Together these results suggest that Fn-EDA partially contributes to α9-mediated SMC proliferation and migration.

### Targeting integrin α9β1 signaling using a specific inhibitor reduces neointimal hyperplasia in wild-type mice by limiting SMC proliferation and migration.

**Figure 7:**
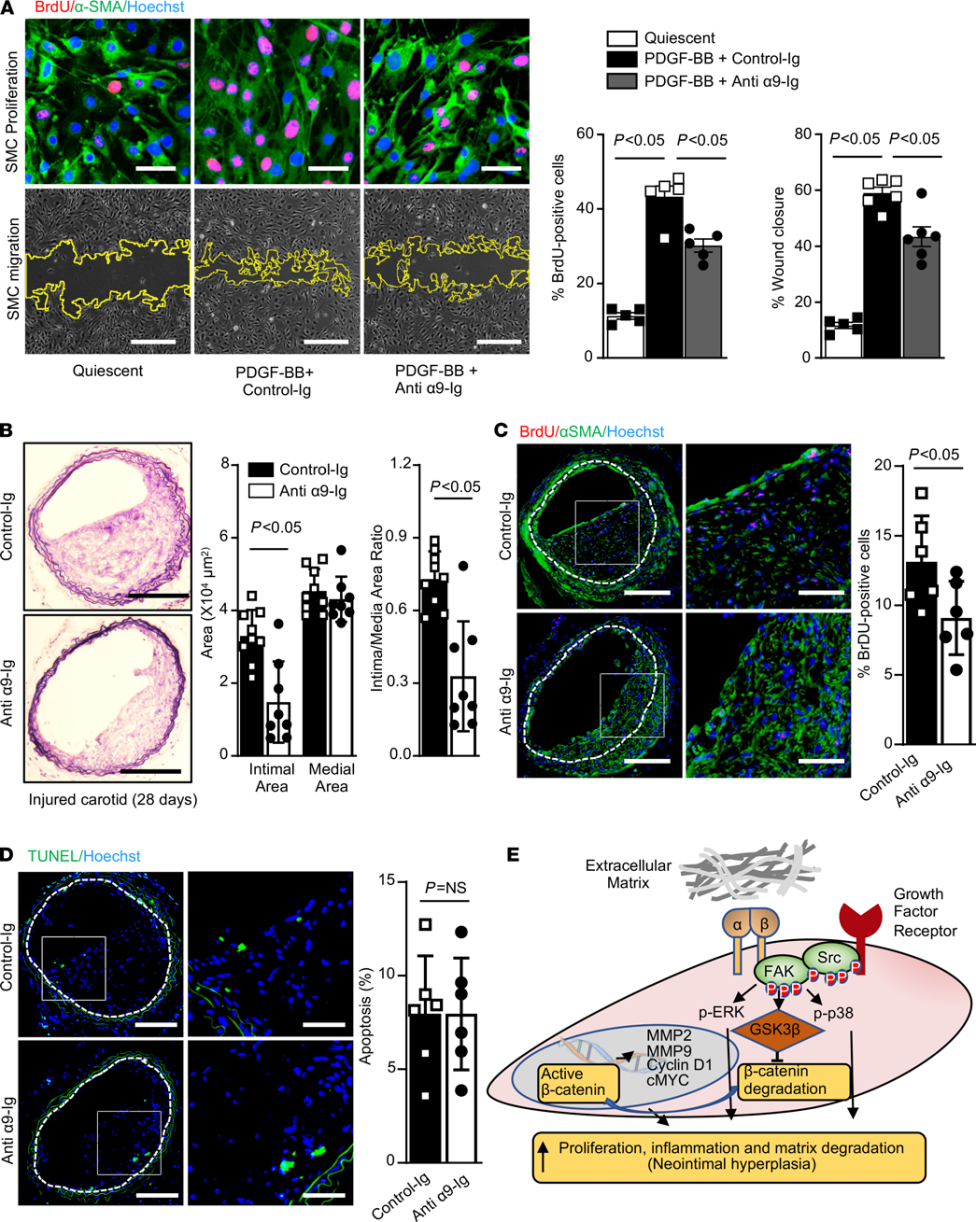
Infusion of anti–integrin α9 antibody reduces injury-induced neointimal hyperplasia in WT mice. Serum-starved SMCs from WT mice were pretreated with murine specific anti-α9 blocking antibody (clone 55A2C, 10 μg/mL) for 60 minutes and then stimulated with or without PDGF-BB for 24 hours. (**A**) The top panels show representative BrdU-positive (red) cells costained with αSMA (green) and Hoechst (blue). Scale bars: 50 μm. The bottom panels show representative phase-contrast images of SMC migration in the scratch assay. Scale bars: 500 μm. The right panel shows the quantification of BrdU-positive cells to the total number of cells (*n* *=* 5) and migrated area (*n* *=* 6). (**B**–**D**) Male WT mice were treated with 55A2C (200 μg/mouse) or control Ig. Wire injury was performed in the carotid artery after 60 minutes, and arteries were harvested after 28 days. (**B**) The left panels show representative photomicrographs of Verhoeff’s van Gieson–stained carotid artery sections. Scale bars: 200 μm. The right panels show quantification of intimal area, medial area, and a ratio of intimal to medial area. Each dot represents a single mouse (*n* = 8–9/group). (**C**) The left panels show representative BrdU-positive cells (red) counterstained with αSMA (green). Nuclei are counterstained with Hoechst (blue). The right panel shows the quantification of percentage BrdU-positive cells (*n* = 6). Scale bars: 200 μm. (**D**) The left panels show representative TUNEL-positive cells (green) counterstained with Hoechst (blue). The right panel shows the quantification of TUNEL-positive cells (*n* = 6). Scale bars: 200 μm. Values are represented as mean ± SEM. Statistical analysis: (**A**) 1-way ANOVA with Bonferroni’s post hoc test; (**B**–**D**) unpaired 2-tailed Student’s *t* test. *P < 0.05 vs. quiescent or vehicle-treated (control Ig) groups. (**E**) Schematic showing the mechanism by which SMC-specific integrin α9 mediates SMC proliferation, migration, and neointimal hyperplasia.

We next evaluated the therapeutic potential of targeting integrin α9 to reduce vascular injury–induced neointimal hyperplasia. Serum-deprived SMCs from wild-type (WT, C57BL/6J) mice were preincubated with murine anti–integrin α9 antibody (clone 55A2C, 10 μg/mL, refs. 23–25) for 1 hour and then stimulated with PDGF-BB. SMC morphology was normal during and after treatment. We found that SMC proliferation and migration were significantly attenuated in the group treated with anti–integrin α9 Ig when compared with the control Ig–treated group (Figure 7A). To determine any off-target effects of 55A2C (10 μg/mL), aortic SMCs derived from α9^SMC-KO^ mice were treated with 55A2C for 60 minutes prior to PDGF-BB stimulation, and SMC proliferation and migration were measured after 24 hours. No inhibitory effect of 55A2C was observed on SMC proliferation or migration in SMCs lacking α9 (Supplemental Figure 7), suggesting that 55A2C at a dose of 10 μg/mL does not exert any off-target effect. We also analyzed mRNA expression of genes regulated by α9 in SMCs from WT mice pretreated with α9 blocking antibody. We found that treatment with anti–integrin α9 Ig significantly inhibited PDGF-BB–induced upregulation of β-catenin–regulated genes including MMP-2, MMP-9, c-Myc, and cyclin D1 (Supplemental Figure 8). Male WT mice were randomly assigned to receive either anti–integrin α9 Ig (200 μg/mouse, intravenous bolus injection) or control Ig, 60 minutes prior to vascular (wire) injury. Susceptibility to neointimal hyperplasia was evaluated 4 weeks after vascular injury. Anti–integrin α9 Ig–treated mice exhibited a marked decrease in the neointimal area and neointimal/medial ratio (*P* < 0.05 vs. vehicle-treated control mice; Figure 7B). Next, we analyzed SMC proliferation and apoptosis in wire-injured carotid artery sections. We found that SMC proliferation was significantly reduced in mice treated with anti–integrin α9 antibody as compared with control Ig–treated mice (Figure 7C). Further analysis revealed that the percentage of BrdU-positive cells was significantly reduced in the neointima but not in the media of α9 antibody–treated mice as compared with control Ig–treated mice (Supplemental Figure 9). Immunostaining revealed an increase in contractile proteins (SM22α and SM-MHC) and a decrease in synthetic proteins (vimentin and osteopontin) in the neointima of anti–integrin α9 Ig–treated mice as compared with control Ig–treated mice (Supplemental Figure 10). Anti–integrin α9 antibody treatment did not affect apoptosis (Figure 7D).

## Discussion

The results of this study suggest that integrin α9β1 is a key factor that regulates several aspects of SMC biology and vascular remodeling. We believe that these findings are novel and may have clinical significance for the following reasons: first, stimulated human coronary SMCs upregulate integrin α9 expression, which may be a prerequisite to coronary SMC proliferation and neointimal hyperplasia. Second, utilizing anti–integrin α9 antibody or SMC-specific α9-deficient mice, we demonstrate integrin α9β1 regulates SMC phenotype switching, proliferation, and migration. SMC-specific α9-deficient mice exhibited reduced injury-induced neointimal hyperplasia. Finally, we provide evidence that deletion of SMC-specific integrin α9 was associated with reduced activation of FAK/Src, ERK, p38, and GSK3β/β-catenin pathway.

Several integrins, including α2β1, α5β1, α5β3, and α4β1, contribute to SMC migration and synthetic phenotype. Previously, it was shown that α9β1 is expressed by vascular SMCs (7). However, no prior studies have examined how lack of α9β1 affects SMC proliferation and neointimal hyperplasia. We demonstrated that cultured SMCs from the human coronary artery upregulated α9 following stimulation with PDGF-BB, which was associated with phenotypic switching from contractile to synthetic state. We speculate that phenotypic switching of SMCs from low to high α9 expression may reduce protective homeostasis in these cells, thus resulting in SMC proliferation and neointimal hyperplasia. Indeed, targeting α9 in stimulated human coronary SMCs, using anti-α9 antibody, suppressed synthetic phenotype and inhibited SMC proliferation and migration. The anti-α9 antibody may have off-target side effects. To provide definitive evidence, we generated α9-deficient mice. β1 is the only known subunit of α9; therefore, lack of α9 should completely inhibit α9β1 signaling. We did not target β1 in addition to α9 because β1 binds to several other subunits, including α4 and α5 that may confound our findings. In line with these observations, we found that genetic ablation of α9 in murine SMCs suppressed synthetic phenotype and reduced proliferation and migration in vitro and inhibited neointimal hyperplasia in vivo.

We also investigated the molecular mechanism by which integrin α9β1 promotes SMC proliferation, migration, and neointimal hyperplasia. We found that therapeutic targeting of α9 in human coronary artery SMCs or genetic deletion of α9 significantly reduced FAK, Src, and MAPK activation, which are known to regulate a wide range of cellular responses, including proliferation and migration. One of the signaling pathways downstream of integrin activation is the canonical Wnt/β-catenin pathway (26). We found that the lack of α9 in SMCs results in reduced level of nonphosphorylated β-catenin, which is considered the active form of β-catenin. GSK3β phosphorylates β-catenin and promotes its ubiquitylation and subsequent proteasomal degradation (15). Integrin and growth factor receptors can activate GSK3β, and the lack of integrin α9 in a cancer cell is associated with higher GSK3β activity (26). In line with these findings, we found reduced p-GSK3β in SMCs lacking α9. Nuclear localized β-catenin interacts with the TCF/LEF family of transcription factors and promotes gene expression. We found that stimulated SMCs lacking α9 had reduced nuclear β-catenin levels associated with decreased transcriptional activation of MMP-2, MMP-9, cyclin D1, and c-Myc. Together, these findings suggest that α9 may contribute to SMC proliferation and migration by upregulating the β-catenin pathway.

Integrins play a crucial role in SMC biology by binding to the extracellular matrix (ECM) with the SMC actin cytoskeleton. Integrin α9β1 binds to several components of ECM proteins, including osteopontin, tenascin-C, and cellular fibronectin-containing EDA (Fn-EDA). Additionally, α9β1 and its matrix protein ligands associate and synergize signaling from several growth factors, including PDGF-BB, to promote cell adhesion motility (4, 27, 28). Integrins such as αvβ3, α5β1, and α8β1 bind to ECM containing an RGD (Arg-Gly-Asp) motif including fibronectin and vitronectin. Unlike other integrins that recognize RGD sequence, α9β1 recognizes several non-RGD sequences, including SVVYGLR in osteopontin (29), AEIDGIEL in tenascin-C (30), and PEDGIHELFP in Fn-EDA (21). The first evidence that plasma fibronectin (which does not contain extra domain A) plays a significant role in phenotypic switching via integrins was demonstrated by Hedin and Thyberg (31). However, integrin α9β1 is a ligand for cellular Fn-EDA but not plasma fibronectin or cellular Fn-EDB. We have previously shown that PDGF-BB upregulates cellular Fn-EDA expression levels in stimulated SMCs and promotes phenotype switching, proliferation, and migration through integrins (not recognized by RGD sequence) and TLR4-dependent pathways (13). Notably, cellular Fn-EDA was more potent than plasma fibronectin in promoting phenotypic switching and the proliferation of SMCs. Integrin α5β1 is another ligand for fibronectin and is known to play a role in SMC proliferation; however, α5β1 recognizes RGD sequence in fibronectin. Because α9β1 recognizes non-RGD sequences and binds explicitly to Fn-EDA, we hypothesized that α9β1 might contribute to SMC phenotypic switching and proliferation via Fn-EDA. Indeed, we found that Fn-EDA partially contributes to integrin α9–mediated SMC phenotypic switching and proliferation. We speculate that other ECM proteins, including osteopontin and tenascin-C, may contribute to integrin α9–mediated SMC phenotypic switching and proliferation in addition to Fn-EDA. A summary of the proposed mechanism is provided in Figure 7E.

Antibody or oligonucleotide infusion or viral vectors have been widely used to inhibit the signaling pathways for the prevention of neointimal hyperplasia (32–36). Next, we evaluated whether injury-induced neointimal hyperplasia could be reduced by blocking α9β1 signaling. Herein, we demonstrate that targeting α9β1 signaling using anti–integrin α9 antibody (55A2C) reduced neointimal hyperplasia in WT mice by limiting SMC proliferation and migration. Anti–integrin α9 antibody is known to inhibit the binding of α9/NIH 3T3 cells to the synthetic peptides AEIDGIEL, the sequence similar to the EDGIHEL sequence present in the EDA of cellular fibronectin (21). Based on these observations, we speculate that the beneficial effects of the anti–integrin α9 antibody on neointimal hyperplasia might be partly due to the inhibition of integrin α9β1 binding to cellular Fn-EDA. Notably, other cells, including neutrophils, hepatocytes, endothelial cells, and epithelial cells, express α9, and the possibility that other cell types may contribute to neointimal hyperplasia cannot be ruled out. Further studies are required to determine the role of integrin α9 in other cell types in neointimal hyperplasia.

A particular strength of our study is that we generated SMC-specific deficient mice to provide conclusive evidence that integrin α9β1 regulates multiple aspects of SMC biology. In WT mice, we demonstrated that targeting α9 by infusing 55A2C antibody reduced neointimal hyperplasia at 28 days. Despite its strength, our study has limitations. We used a wire injury model that partially mimics balloon angioplasty or in-stent restenosis. Future studies are warranted to confirm the efficacy of targeting integrin α9 in in-stent models. We suggest that therapeutically targeting integrin α9 may be beneficial in patients to reduce intimal hyperplasia during both open and endovascular procedures.

## Methods

### Antibodies.

A detailed list of antibodies with catalog number and company is provided in Supplemental Table 2.

### Animals.

The α9^fl/fl^ mouse strain has been described previously (37) (provided by Matthew G. Vander Heiden, MIT, Boston, Massachusetts, USA). To generate SMC-specific α9-deficient mice (α9^fl/fl^ SM22αCre^+^ Apoe^–/–^), α9^fl/fl^ Apoe^–/–^ mice (generated in-house) were crossed with SM22αCre^+^ Apoe^–/–^ mice (provided by Isabella Grumbach, University of Iowa, Iowa City, Iowa, USA).

The SM22αCre^+^ transgenic mice expressed Cre recombinase under the control of mouse SMC protein 22α (or transgelin) promoter. Littermates (α9^fl/fl^ SM22αCre^–^ Apoe^–/–^) were used as controls. Fn-EDA^–/–^ Apoe^–/–^ mice (generated in-house) have been described previously (38) (Fn-EDA^–/–^ mice provided by Andrés F. Muro, ICGEB, Trieste, Italy). All the mouse strains are on a C57BL/6J background (backcrossed >15 times). Mice were genotyped by PCR according to protocols from The Jackson Laboratory and as described previously (38). C57BL/6J mice (7–8 weeks old, males) were procured from The Jackson Laboratory. Mice were kept in standard animal housing conditions with controlled temperature and humidity and had ad libitum access to standard chow diet and water. All the experiments involving animals were performed according to protocols approved by the University of Iowa Animal Care and Use Committee according to the current Animal Research: Reporting of *In Vivo* Experiment guidelines (https://www.nc3rs.org.uk/revision-arrive-guidelines).

### Mouse carotid artery injury model of neointimal hyperplasia.

Carotid artery wire injury was performed by using a guidewire (0.015-inch diameter, C-SF-15-15, Cook Medical) as described previously (39). Briefly, the left common carotid artery was exposed by blunted dissection. The external carotid artery was exposed, and an incision was made through which the guidewire was advanced 1 cm toward the aorta. The wire was moved back and forth 3 times to ensure endothelial denudation. The wire was retrieved, and the external carotid artery was ligated with a 7-0 vicryl suture. The wound was closed with a subcuticular 7-0 vicryl suture. Animals were allowed to recover before returning to the cages. At 28 days after injury, the animals were euthanized, and carotid arteries (~5.0 mm from injury site) were carefully harvested and processed for cryostat sectioning for subsequent analysis. Some animals received 2 subcutaneous doses of BrdU (Millipore Sigma, 100 mg/kg) at 12 hours and 1 hour prior to the mice being sacrificed (40).

### Injection of anti–integrin α9 antibody.

Mice were randomly assigned and infused with either anti-α9 antibody (55A2C, 200 μg/mouse, provided as a gift from Gene Techno Science Co Ltd) or control Ig isotype (200 μg/mouse, Rockland, catalog 610-4107-0500) intravenously (41, 42), 60 minutes prior to carotid artery wire injury.

### Histology and morphometric analysis.

To quantify intimal hyperplasia, serial 5 μm cross sections from injured and noninjured arterial segments were stained with Verhoeff’s van Gieson stain as described (43). Bright-field images were acquired using an Olympus BX51 microscope equipped with a ×20 objective. Circumferences of the external and internal elastic lamina (EEL, IEL) and the lumen were traced on the stained sections (44). The intimal area was determined by subtracting the lumen area from the area circumscribed by the IEL traced on stained sections, whereas medial area was defined as the area between an EEL and an IEL (45). Intima/media area ratio was calculated by dividing the intimal area by medial area (46). Calculations were made using the mean value of 4–6 sections (each approximately 100 μm apart) from each mouse artery. NIH ImageJ software was used for quantification.

### Human and mouse SMC culture and treatment.

Human primary coronary artery SMCs were obtained from Lonza (catalog number CC-2583) and cultured in smooth muscle basal medium (Lonza; catalog number CC-3181) containing recombinant human FGF basic (5 ng/mL), insulin (5 μg/mL), EGF (5 ng/mL), and FBS (5%). Primary murine SMCs were isolated from the thoracic aorta by enzymatic digestion technique as described previously (47). Briefly, the thoracic aorta was dissected and cleaned of adventitial tissue and incubated with 1.0 mg/mL collagenase in DMEM at 37°C for 90 minutes. The cell suspension was passed through a 70 μm restrainer, washed, and cultured in DMEM with 10% FBS. Cells were maintained in a 95% air and 5% CO_2_ humidified incubator at 37°C for 7 days, trypsinized with 0.25% trypsin-EDTA, and subcultured. All SMCs used for experiments were between the third and fifth passages.

### EDA-containing and EDA-lacking recombinant peptides.

Fibronectin sequences containing [EDA (+) peptide] or lacking [EDA (-) peptide] the EDA segment were generated from full-length cellular fibronectin (cFn) and full-length plasma fibronectin (pFn) cDNAs, respectively, as described before (48), and were used at a concentration of 10 μg/mL.

### Immunocytochemistry.

Immunostaining was performed in sections from mouse carotid arteries and isolated mouse and human SMCs. Mouse arterial sections or cells were fixed in 4% paraformaldehyde (PFA, in PBS) for 10 minutes and permeabilized with 0.2% Triton X-100 (in PBS). Nonspecific binding was blocked for 1 hour with 5% serum from the species in which the secondary antibody was raised. Samples were incubated overnight at 4°C with antibodies specific for integrin α9 (1:100, Abcam), vimentin (1:100, Abcam), osteopontin (1:100, Abcam), SM-MHC (1:100, Abcam), SM22α (1:100, Abcam), and αSMA (1:200, MilliporeSigma) overnight at 4°C. Isotype control Ig was used as a negative control. After overnight incubation, cells/slides were washed with PBS and labeled with appropriate secondary antibodies (Alexa Fluor 488 or Alexa Fluor 546 at 1:400, Abcam) for 1 hour at room temperature. Nuclei were stained with Hoechst (5 μg/mL) before mounting. Nikon Eclipse Ti-U inverted fluorescence microscope equipped with a ×20/0.8 Plan Apo lens, cooled charge-coupled device camera, and NIS-Elements imaging software (Nikon) was used for imaging of SMCs. For arterial sections, images were taken using an Olympus BX51 fluorescence microscope. Images were acquired under identical imaging conditions. Mean fluorescence intensity was quantified using the ImageJ software, as previously described (49). Measurements were obtained from 1–2 different fields per murine section and 4–6 different fields for cultured SMCs.

### BrdU incorporation assay.

Human and mouse SMCs were serum-starved for 48 hours to induce quiescence. Cells were washed and stimulated with PDGF-BB (20 ng/mL), cFn (20 μg/mL) or pFn (20 μg/mL), and recombinant EDA-containing or EDA-lacking peptides (10 μg/mL) for 24 hours. Cells were incubated with 10 μM BrdU for the last 12 hours of treatment (50). Carotid artery sections from BrdU-treated mice or cultured SMCs were fixed with 4% PFA (in PBS). DNA hydrolysis was performed by treating the cells/sections with 2 M HCl for 20 minutes. Samples were subjected to immunofluorescence staining with a mouse monoclonal anti-BrdU antibody (1:200, Abcam) together with αSMA (1:200, MilliporeSigma) for 3 hours at 37°C and labeled with appropriate Alexa Fluor 488 and Alexa Fluor 546 secondary antibodies (1:400, Abcam). Nuclei were stained with Hoechst (5 μg/mL) before mounting. Images of the sections were acquired using an Olympus BX51 fluorescence microscope, and images of cells were acquired using the Nikon Eclipse Ti-U fluorescence microscope. The number of BrdU-positive cells in the neointima or the media and the total number of nuclei were determined using ImageJ software. The percentage of BrdU-positive cells was calculated as (number of BrdU-positive nuclei in the neointima or media/total number of nuclei) × 100.

### Migration assay.

Cultured human and mouse aortic SMCs were seeded in 6-well culture plates and were grown in DMEM until confluence. Cells were serum-starved for 48 hours, and a manual scratch was created with a 200 μL pipette tip to create a cell-free zone. Cells were washed with PBS to remove nonadherent cells and incubated in a serum-free medium. Images of the scratched area were taken immediately using a Nikon Eclipse Ti-U phase-contrast microscope. Cells were then stimulated with PDGF-BB (20 ng/mL) and recombinant EDA-containing or EDA-lacking peptides (10 μg/mL) for 24 hours. The second set of images were taken after 24 hours to measure SMC migration over the scratched area. The cell-free area for each well was measured using the ImageJ software and the MRI wound healing tool plug-in (51). The percentage of wound closure was calculated as the difference of denuded area between 0 and 12 or 24 hours to the denuded area at 0 hours.

### Western blotting.

Cell or tissue proteins were extracted with RIPA lysis buffer containing protease inhibitor cocktail. For nuclear extraction experiments, nuclear and cytoplasmic fractions were isolated using the NE-PER Nuclear and Cytoplasmic Extraction kit (Invitrogen, Thermo Fisher Scientific), following the manufacturer’s instructions. Equal amounts of protein samples were resolved by SDS-PAGE and transferred to PVDF membranes by using a Bio-Rad Western blotting system. The membranes were blocked with 5% BSA or 5% nonfat dry milk in Tris-buffered saline containing 0.1% Tween 20 for 1 hour at room temperature. The membrane was then incubated overnight at 4°C with primary antibodies against integrin α9, p-FAK (Tyr397), FAK, p-Src family (Tyr416), Src, p-p44/42 MAPK (Erk1/2) (Thr202/Tyr204), p44/42 MAPK (Erk1/2), p-GSK3β, GSK3β, active β-catenin, total β-catenin, and β-actin. Following incubation with appropriate horseradish peroxidase–conjugated secondary antibodies, the blots were visualized and imaged using ECL Plus Kit (MilliporeSigma) and ChemiDoc Imaging System (Bio-Rad). Densitometric analysis of the gels was done using ImageJ software. The proteins were normalized with β-actin (MilliporeSigma) and respective total protein content. See complete unedited blots in the supplemental material.

### TUNEL staining.

Apoptotic cells within the carotid artery sections were visualized using In Situ Cell Death Detection Kit, Fluorescein (Roche, 11684809910), as per manufacturer’s instructions. Briefly, arterial sections were fixed, permeabilized with 0.2% Triton X-100, and subsequently incubated with TUNEL reaction mixture (Roche) for 60 minutes at 37°C in a humidified chamber. Nuclei were stained with Hoechst (5 μg/mL). Sections were examined under a fluorescence microscope (Olympus BX51). The percentage of apoptotic cells to the total number of cells was quantified using ImageJ software as described previously (52, 53).

### RNA isolation and quantitative real-time PCR analysis.

Total cellular RNA was isolated from murine SMCs using an RNeasy Kit (QIAGEN, 74104) as per the manufacturer’s instructions. cDNA was prepared using iScript Reverse Transcription Supermix (Bio-Rad, 1708840). PCR amplification of the cDNA (100 ng) was performed with the Applied Biosystems 7900HT Fast Real-Time PCR machine (total volume 20 μL, Thermo Fisher Scientific) at the University of Iowa’s Iowa Institute of Human Genetics Genomics Division. Levels of mRNA were normalized by GAPDH (as a loading control) and calculated according to the comparative threshold cycle (ΔCt value). Primers for quantitative real-time PCR for the indicated genes are included in Supplemental Table 3.

### Statistics.

Data are expressed as mean ± SEM. The number of animals in each group was based on power calculations for the primary parameter (neointimal area) with mean differences and SDs taken from pilot data at power 80% with an α of 0.05. GraphPad Prism (version 8.4.1) was used for statistical analysis. The Shapiro-Wilk test was used to check normality, and Bartlett’s test was used to check equal variance. The statistical significance of the difference between means was assessed using the unpaired 2-tailed Student’s *t* test (for the comparison of 2 groups), by 1-way ANOVA followed by Bonferroni’s multiple comparisons tests (parametric data of more than 2 groups), by Kruskal-Wallis test followed by uncorrected Dunn’s test (nonparametric data of more than 2 groups), or by 2-way ANOVA followed by uncorrected Fisher’s LSD test. *P* < 0.05 was considered significant.

### Study approval.

The University of Iowa Animal Care and Use Committee approved all procedures, and studies were performed according to the current Animal Research: Reporting of *In Vivo* Experiment guidelines (https://www.nc3rs.org.uk/revision-arrive-guidelines).

## Author contributions

MJ and AKC conceived the study; MJ, RD, PD, and ND analyzed data; MJ, RD, PD, and ND investigated; SK provided reagents; MJ and AKC wrote the original draft; and all authors reviewed and edited the draft.

## Supplementary Material

Supplemental data

## Version 1 05/24/2021

Electronic publication
