# Supplementary material for: Integrin α9 regulates smooth muscle cell phenotype switching and vascular remodeling
Source: JCI Insight. 2021 May 24;6(10):e147134. doi: 10.1172/jci.insight.147134 (PMC8262341; doi:10.1172/jci.insight.147134)

Data Supplement

Integrin α9 regulates smooth muscle cell phenotype switching and vascular remodeling

Running title: *Jain et al.: Targeting integrin α9 inhibits neointimal hyperplasia.*

Manish Jain*[1], Rishabh Dev[1], Prakash Doddapattar, Shigeyuki Kon[2], Nirav Dhanesha[1], Anil K. Chauhan*[1]

[1]Department of Internal Medicine, Division of Hematology/Oncology, University of Iowa, Iowa City, IA.

[2]Department of Molecular Immunology, Faculty of Pharmaceutical Sciences, Fukuyama University, Hiroshima, Japan.

Supplementary Tables

**Complete blood count**	$\alpha9^{fl/fl}$	$\alpha9^{SMC-KO}$
WBC ($10^3/\mu L$)	11 ± 2.9	10.5 ± 2.6
RBC ($10^6/\mu L$)	8.0 ± 2.0	8.6 ± 2.2
HGB (g/dL)	11.8 ± 2.9	12.2 ± 3.1
HCT (%)	38.2 ± 9.6	39.5 ± 9.9
Platelets ($10^3/\mu L$)	1008± 252	1034 ± 262
Neutrophil ($10^3/\mu L$)	0.2 ± 0.1	0.2 ± 0.1
Lymphocytes ($10^3/\mu L$)	7.1 ± 2.2	7.5 ± 2.1
Monocytes ($10^3/\mu L$)	1.8 ± 0.5	1.5 ± 0.4

Table S1: Complete blood counts from 8-10 weeks old mice were obtained using an automated veterinary hematology analyzer (ADVIA). Value are expressed as mean ± SEM. N= 5-6 mice/group. P= Non-significant versus control mice. WBC, White Blood cells; RBC, Red Blood cells; HGB, Hemoglobin; HCT, Hematocrit.

Target antigen	Vendor or Source	Cat#	Persistent ID / URL
Integrin α9	Abcam	ab140599	https://www.abcam.com/integrin-alpha-9-antibody-epr9722-ab140599.html
Integrin β1	Cell Signaling Technology	#4706	https://www.cellsignal.com/products/primary-antibodies/integrin-b1-antibody/4706
α-SMA	Sigma	#A5228	https://www.sigmaaldrich.com/catalog/product/sigma/a5228?lang=en&region=US
SM22 alpha	Abcam	ab14106	https://www.abcam.com/taglntransgelin-antibody-ab14106.html
Smooth Muscle Myosin Heavy Chain 11	Abcam	ab683 ab82541	https://www.abcam.com/smooth-muscle-myosin-heavy-chain-11-antibody-1g12-ab683.html https://www.abcam.com/smooth-muscle-myosin-heavy-chain-11-antibody-ab82541.html
Vimentin	Abcam	ab92547	https://www.abcam.com/vimentin-antibody-epr3776-cytoskeleton-marker-ab92547.html
Osteopontin	Abcam	ab8448	https://www.abcam.com/osteopontin-antibody-ab8448.html
BrdU	Abcam	ab6326	https://www.abcam.com/brdu-antibody-bu175-icr1-proliferation-marker-ab6326.html
Phospho-FAK (Tyr397)	Cell Signaling Technology	#8556	https://www.cellsignal.com/products/primary-antibodies/phospho-fak-tyr397-d20b1-rabbit-mab/8556?site-search-type=Products&N=4294956287&Ntt=8556p&fromPage=plp&_requestid=1979083
FAK	Cell Signaling Technology	#3285	https://www.cellsignal.com/products/primary-antibodies/fak-antibody/3285?site-search-type=Products&N=4294956287&Ntt=3285s&fromPage=plp&_requestid=1979244
Phospho-Src Family (Tyr416)	Cell Signaling Technology	#2101	https://www.cellsignal.com/products/primary-antibodies/phospho-src-family-tyr416-antibody/2101?site-search-type=Products&N=4294956287&Ntt=2101s&fromPage=plp&_requestid=1979427
Src	Cell Signaling Technology	#2109	https://www.cellsignal.com/products/primary-antibodies/src-36d10-rabbit-mab/2109?site-search-type=Products&N=4294956287&Ntt=2109s&fromPage=plp&_requestid=1979578
Phospho-p44/42	Cell Signaling Technology	#4370	https://www.cellsignal.com/products/primary-antibodies/phospho-p44-42-mapk-erk1-2-thr202-tyr204-d13-14-4e-xp-rabbit-mab/4370?site-search-

MAPK (Erk1/2)			type=Products&N=4294956287&Ntt=4370s&fromPage=plp&_requestid=1979840
p44/42 MAPK (Erk1/2)	Cell Signaling Technology	#4695	https://www.cellsignal.com/products/primary-antibodies/p44-42-mapk-erk1-2-137f5-rabbit-mab/4695?site-search-type=Products&N=4294956287&Ntt=4695s&fromPage=plp&_requestid=1984322
Phospho-p38 MAPK	Cell Signaling Technology	#9211	https://www.cellsignal.com/products/primary-antibodies/phospho-p38-mapk-thr180-tyr182-antibody/9211?site-search-type=Products&N=4294956287&Ntt=9211s&fromPage=plp&_requestid=1984437
p38 MAPK Antibody	Cell Signaling Technology	#9212	https://www.cellsignal.com/products/primary-antibodies/p38-mapk-antibody/9212
Phospho-GSK-3-beta (Ser9)	Cell Signaling Technology	#9322	https://www.cellsignal.com/products/primary-antibodies/phospho-gsk-3-beta-ser9-d3a4-rabbit-mab/9322
GSK-3β	Cell Signaling Technology	#9315	https://www.cellsignal.com/products/primary-antibodies/gsk-3b-27c10-rabbit-mab/9315
Non-phospho (Active) β-Catenin (Ser33/37/Thr41)	Cell Signaling Technology	#8814	https://www.cellsignal.com/products/primary-antibodies/non-phospho-active-b-catenin-ser33-37-thr41-antibody/4270?site-search-type=Products&N=4294956287&Ntt=4270s&fromPage=plp&_requestid=1984907
β-catenin	Cell Signaling Technology	#8480	https://www.cellsignal.com/products/primary-antibodies/b-catenin-d10a8-xp-rabbit-mab/8480
Lamin B1	Abcam	ab65986	https://www.abcam.com/lamin-b1-antibody-ab65986.html
GAPDH	Cell Signaling Technology	#2118	https://www.cellsignal.com/products/primary-antibodies/gapdh-14c10-rabbit-mab/2118
β-Actin	Abcam	ab8227	https://www.abcam.com/beta-actin-antibody-ab8227.html
goat anti-rat 488	Invitrogen	A11006	https://www.thermofisher.com/antibody/product/Goat-anti-Rat-IgG-H-L-Cross-Adsorbed-Secondary-Antibody-Polyclonal/A-11006

goat anti-rabbit 546	Invitrogen	A11035	https://www.thermofisher.com/antibody/product/Goat-anti-Rabbit-IgG-H-L-Highly-Cross-Adsorbed-Secondary-Antibody-Polyclonal/A-11035
goat anti-mouse 488	Invitrogen	A32723	https://www.thermofisher.com/antibody/product/Goat-anti-Mouse-IgG-H-L-Highly-Cross-Adsorbed-Secondary-Antibody-Polyclonal/A32723
goat anti-rat 546	Invitrogen	A11081	https://www.thermofisher.com/antibody/product/Goat-anti-Rat-IgG-H-L-Cross-Adsorbed-Secondary-Antibody-Polyclonal/A-11081
Goat-anti mouse	DAKO	P044701-02	https://www.agilent.com/en/product/immunohistochemistry/antibodies-controls/secondary-antibodies/goat-anti-mouse-immunoglobulins-hrp-(affinity-isolated)-153239
Goat-anti rabbit	DAKO	P048801-02	https://www.agilent.com/en/product/immunohistochemistry/antibodies-controls/secondary-antibodies/goat-anti-rabbit-immunoglobulins-hrp-(affinity-isolated)-153244

Table S2. List of Antibodies

Name	Sequence (from 5′ to 3′)
mouse MMP-2	Forward: GATGTCGCCCCTAAAACAGA<br>Reverse: TGGTGTTCTGGTCAAGGTCA
mouse MMP-9	Forward: AGACCTGAAAACCTCCAACCTCAC<br>Reverse: TGTTATGATGGTCCCACTTGAGGC
mouse c-Myc	Forward: AATCCTGTACCTCGTCCGATTCCA<br>Reverse: TTTGCCTCTTCTCCACAGACACCA
mouse Cyclin D1	Forward: ACCTGGGCAGCCCCAACAAC<br>Reverse: GGAGGCAGTCCGGGTCACACT
mouse GAPDH	Forward: ATGGTGAAGGTCGGTGTGAACG<br>Reverse: CGCTCCTGGAAGATGGTGATGG

Table S3. RT-PCR primer sequences

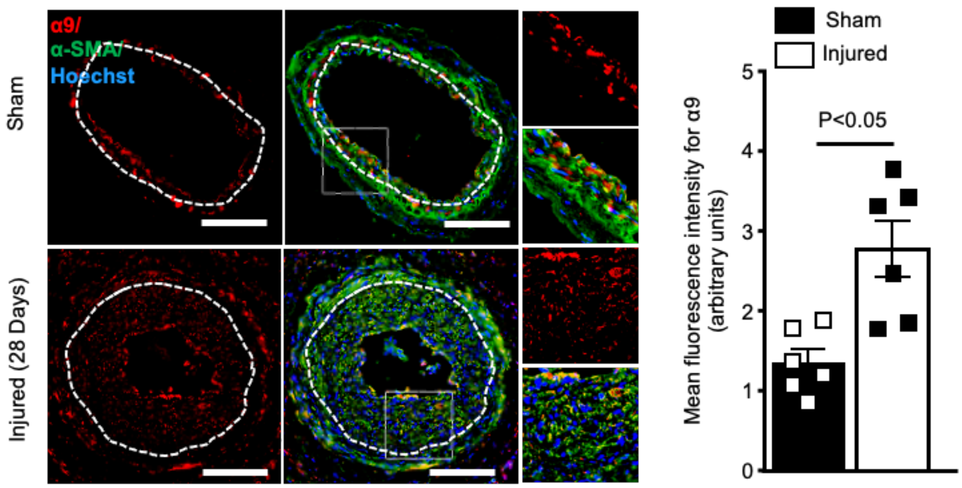

Figure S1. Integrin α9 expression at 28 days in a wire carotid injury model. The left panels show representative double immunostaining for α9 (red) and αSMA (green) in carotid artery sections of WT mice harvested after 28 days of wire injury. The right panel show quantification of α9 fluorescence intensity (n = 6). Scale bar: 200 µm. Boxed regions are magnified. Values are expressed as mean ± SEM. Statistical analysis: unpaired student t-test.

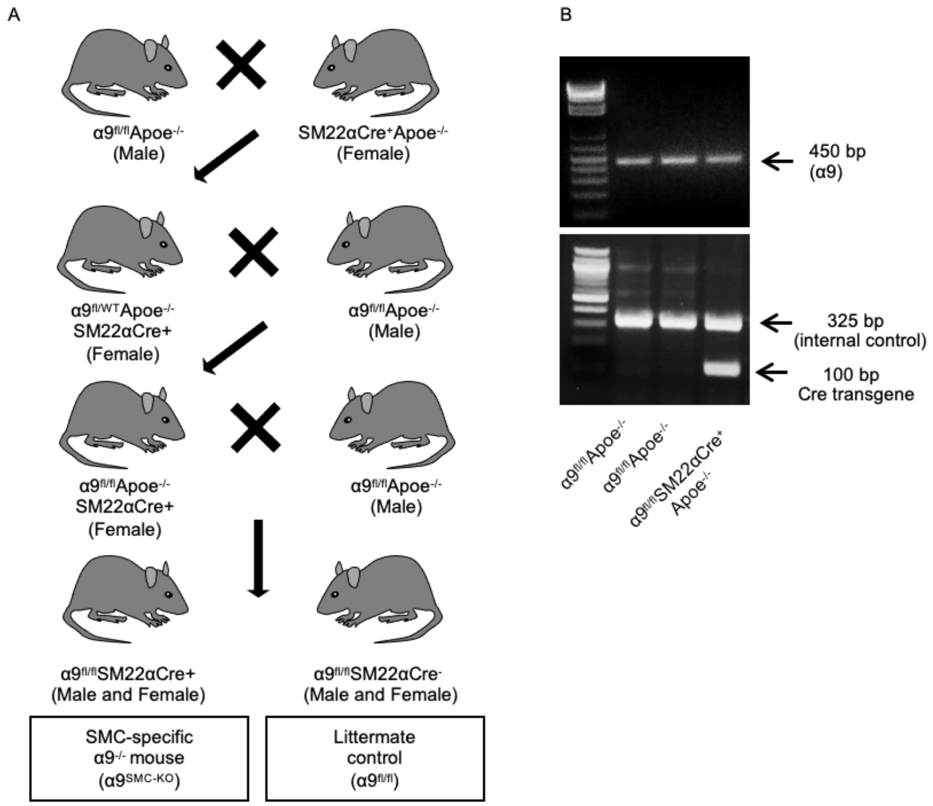

Figure S2. Characterization of SMC-specific α9-deficient mice. (A) Schematic showing the strategy to generate SMC-specific $\alpha 9^{-/-}$ mice. (**B)** Genomic PCR showing the presence of Cre gene in $\alpha 9^{fl/fl}$SM22αCre$^{+/-}$ mice.

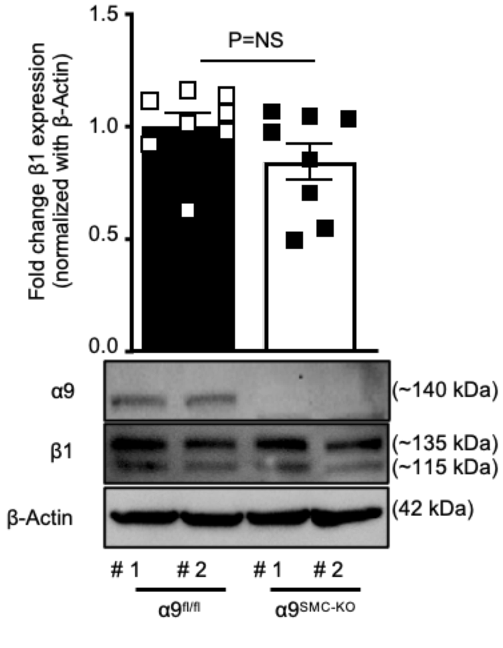

Figure S3. Genetic deletion of integrin α9 in smooth muscle cell does not change integrin β1 expression. Western blot analysis of α9, β1 and β-actin in SMCs isolated from α9^{SMC-KO} and control α9$^{fl/fl}$ mice (*n* = 8). #1 and #2 represents samples from two individual mice. Values are expressed as mean ± SEM. Statistical analysis: unpaired student t-test.

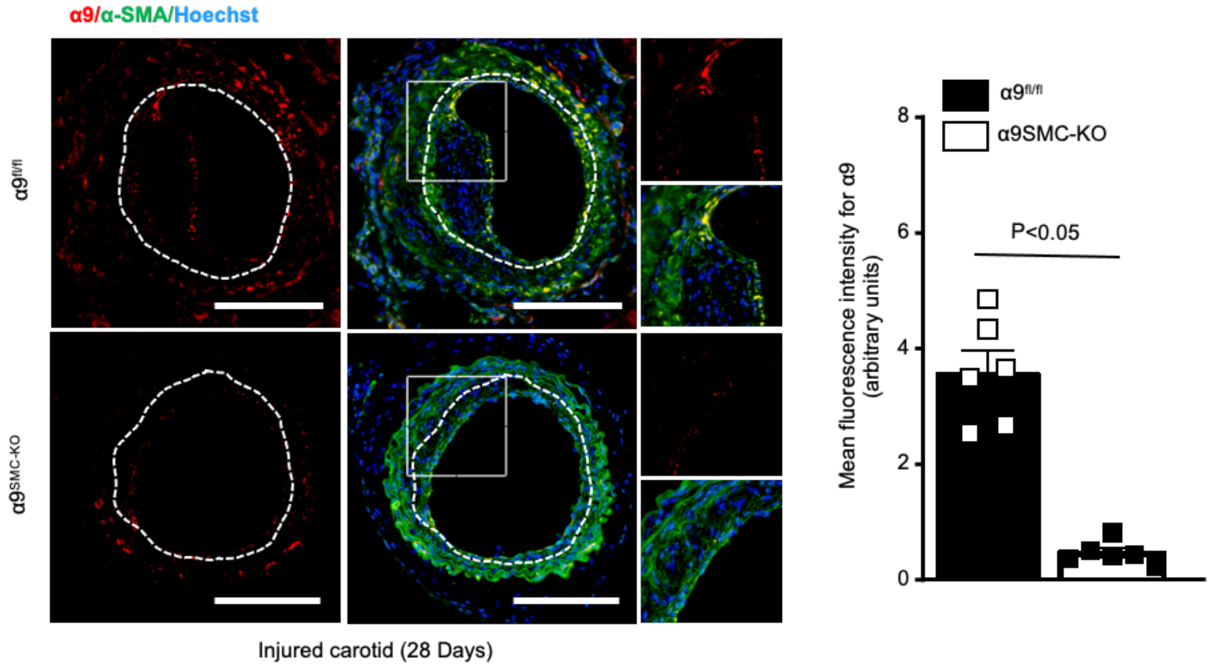

Figure S4. Integrin α9 expression at 28 days. The left panels show representative double immunostaining for α9 (red) and αSMA (green) in carotid artery sections of $\alpha9^{fl/fl}$ and $\alpha9^{SMC\text{-}KO}$ mice harvested after 28 days of wire injury. The right panel show quantification of α9 fluorescence intensity (n = 6). Scale bar: 200 µm. Boxed regions are magnified. Values are expressed as mean ± SEM. Statistical analysis: unpaired student t-test.

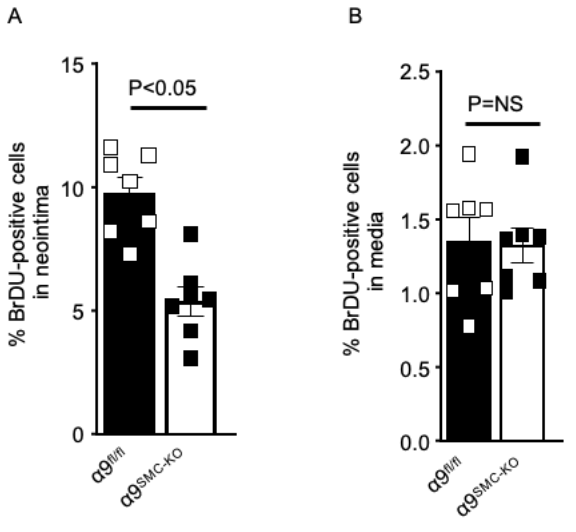

Figure S5. SMC-specific α9 deletion suppresses SMC proliferation *in vivo*. Bar graph representing quantification of percentage BrdU-positive cells in the **(A)** neointima and **(B)** media (n = 7). Values are expressed as mean ± SEM. Statistical analysis: unpaired Student's t-test.

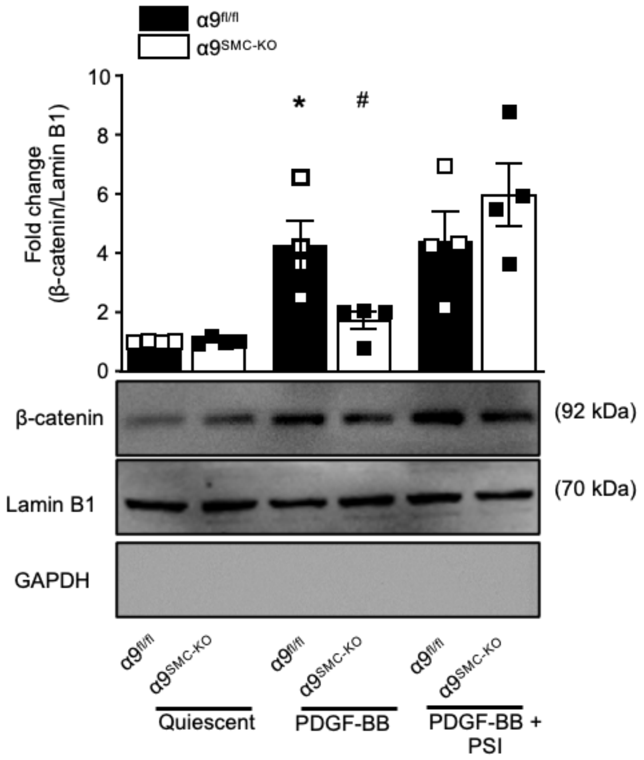

Figure S6. SMC-specific α9 promotes β-catenin degradation through proteasome pathway. Aortic SMCs isolated from α9^{SMC-KO} and control α9$^{fl/fl}$ mice were serum-starved and stimulated with PDGF-BB for 6 h in the presence or absence of proteasome-specific inhibitor (PSI, 10 μM). β-catenin and Lamin B1 were detected by immunoblotting in the nuclear extract (n = 4). Values are expressed as mean ± SEM. Statistical analysis: Two-way ANOVA followed by Fischer LSD test. *P < 0.05 vs. α9$^{fl/fl}$ quiescent SMCs; $^{\#}$P<0.05 vs α9$^{fl/fl}$ PDGF-BB treated SMCs.

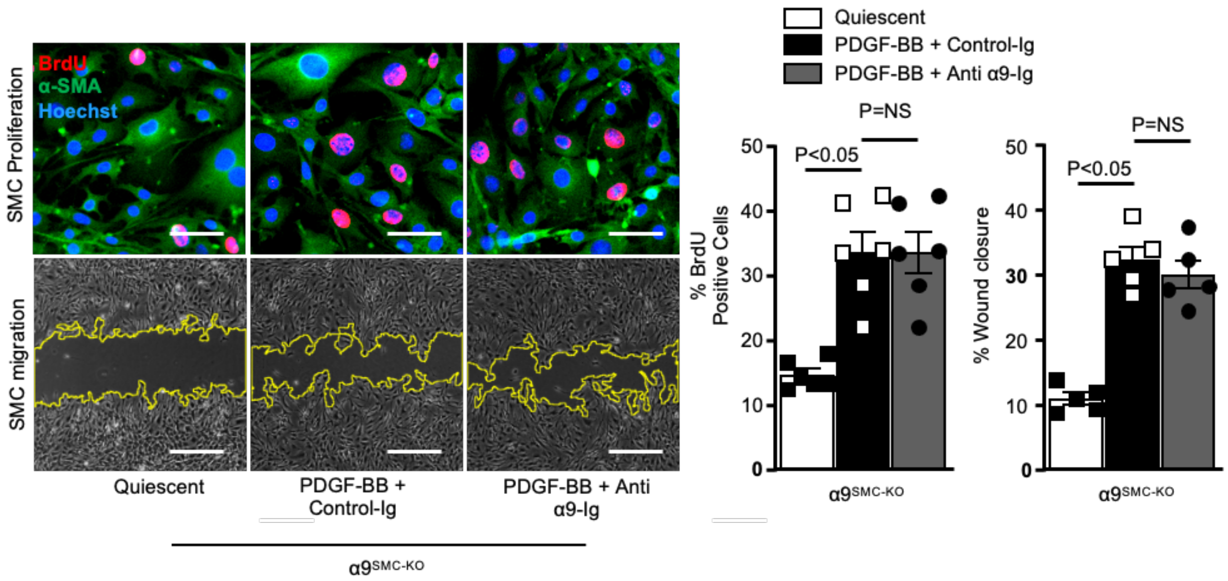

Figure S7. Anti-integrin α9 antibody does not have any off-target effect. Serum-starved SMCs from α9$^{SMC\text{-}KO}$ were pretreated with murine specific anti-α9 blocking antibody (clone 55A2C, 10 µg/ml) for 60 minutes and then stimulated with or without PDGF-BB for 24 h. The top panels show representative BrdU-positive cells co-stained with αSMA (green) and Hoechst (blue). Scale bars: 50 µm. The bottom panels show representative phase-contrast images of SMC migration in the scratch assay. Scale bars: 500 µm. The right panel shows the quantification of BrdU-positive cells to the total number of cells (n = 6) and migrated area (n = 5). Values are represented as mean ± SEM. Statistical analysis: One-way ANOVA with Bonferroni's post hoc test.

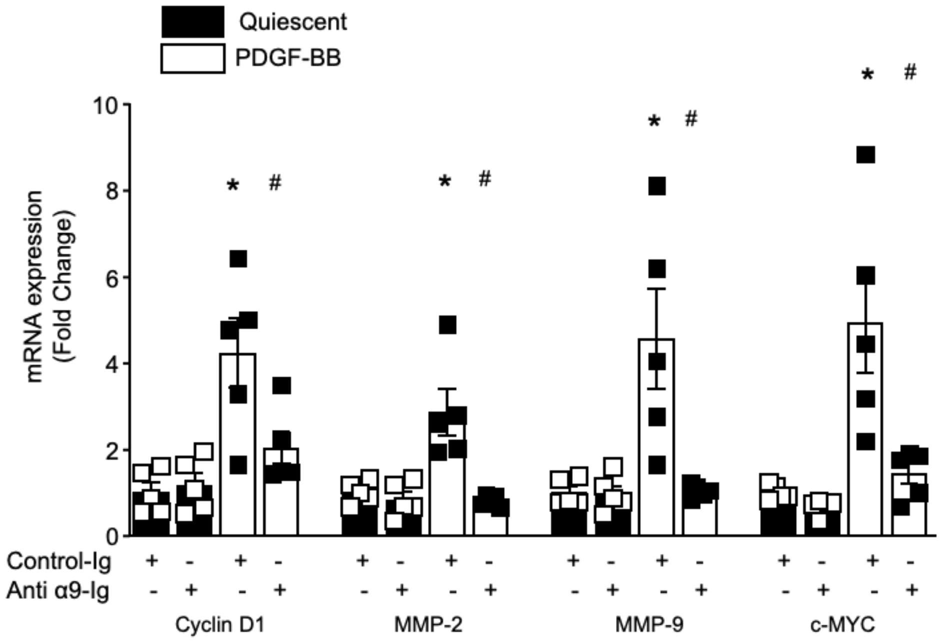

Figure S8. Treatment with anti-integrin α9 antibody suppresses transcription of β-catenin regulated genes. Serum-starved SMCs from WT mice were pretreated with murine specific anti-α9 blocking antibody (clone 55A2C, 10 µg/ml) for 60 minutes and then stimulated with or without PDGF-BB for 24 h. Real-time quantitative PCR analysis of Cyclin D1, MMP-2, MMP-9 and cMYC (n = 5). Values are expressed as mean ± SEM. Statistical analysis: Two-way ANOVA followed by Fischer LSD test. One-way ANOVA with Bonferroni's post hoc test. $^{*}P < 0.05$ vs. quiescent SMCs treated with control-Ig. $^{\#}P < 0.05$ vs. PDGF-BB stimulated SMCs treated with control-Ig.

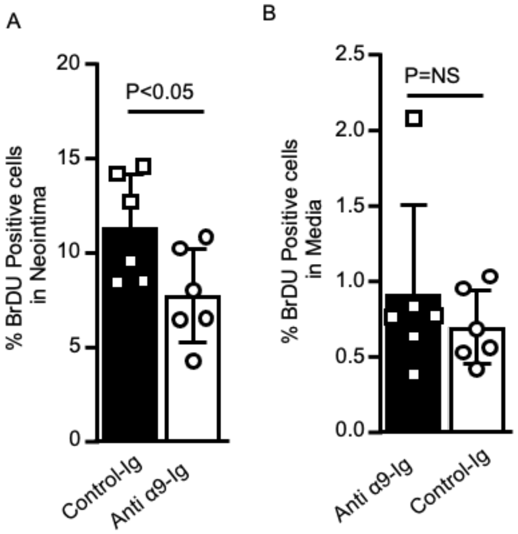

Figure S9. Infusion of anti-integrin α9 antibody reduces SMC proliferation *in vivo*. Bar graph representing quantification of percentage BrdU-positive cells in the **(A)** neointima and **(B)** media (n = 6) of WT mice treated with anti-integrin α9 antibody. Statistical analysis: Mann Whitney test.

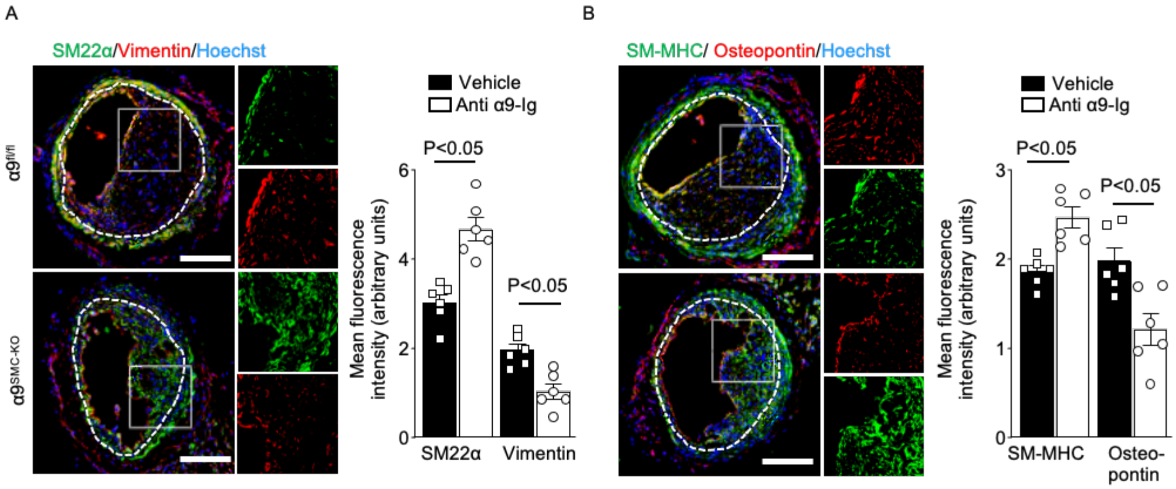

Figure S10. Infusion of anti-integrin α9 antibody modulates SMC phenotypic switching *in vivo*. Male WT mice were treated with 55A2C (200 µg/mouse) or control Ig. Wire injury was performed in the carotid artery after 60 minutes, and arteries were harvested after 28 days. **(A)** The left panels show representative immuno-stained carotid artery sections for SM22α (green) and vimentin (red). The right panels show quantification of fluorescence intensity (n = 6). **(B)** The left panels show representative immuno-stained carotid artery sections for SM-MHC (green) and osteopontin (red). The right panels show quantification of fluorescence intensity (n = 6). Boxed regions are magnified. Scale bars: 200 µm. Statistical analysis: unpaired Student's t-test.

Full unedited gel for Figure 1A- α9

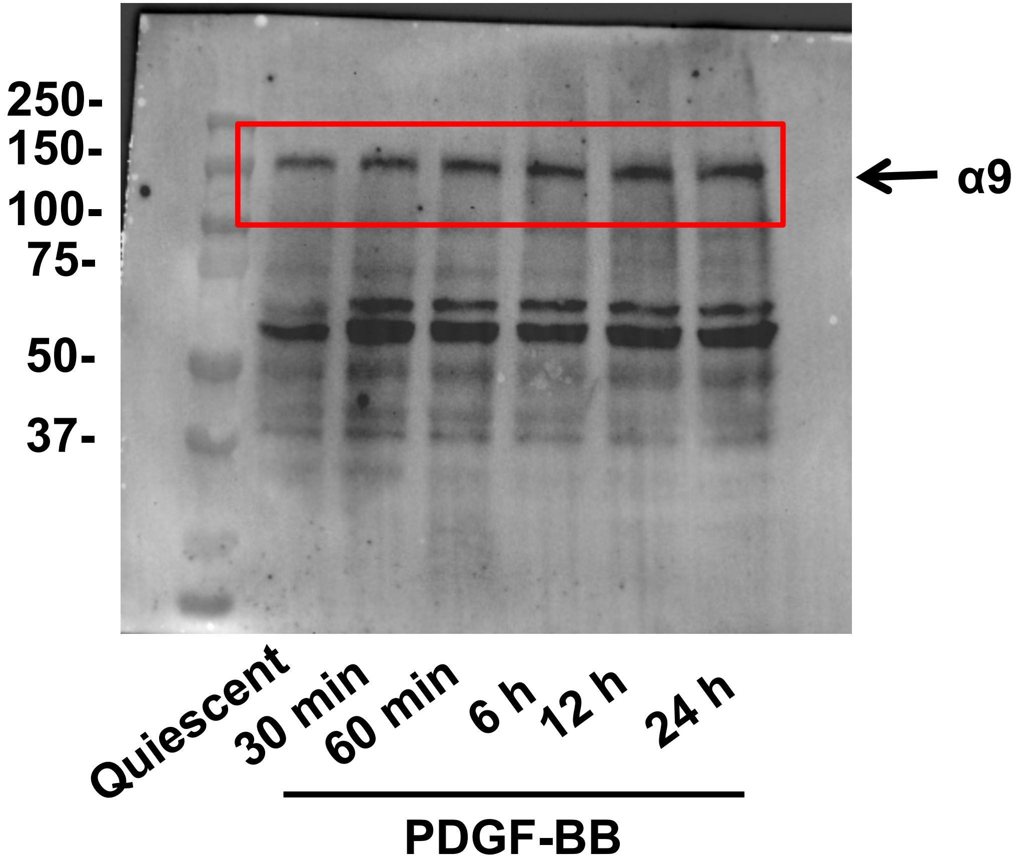

Full unedited gel for Figure 1A- β-Actin

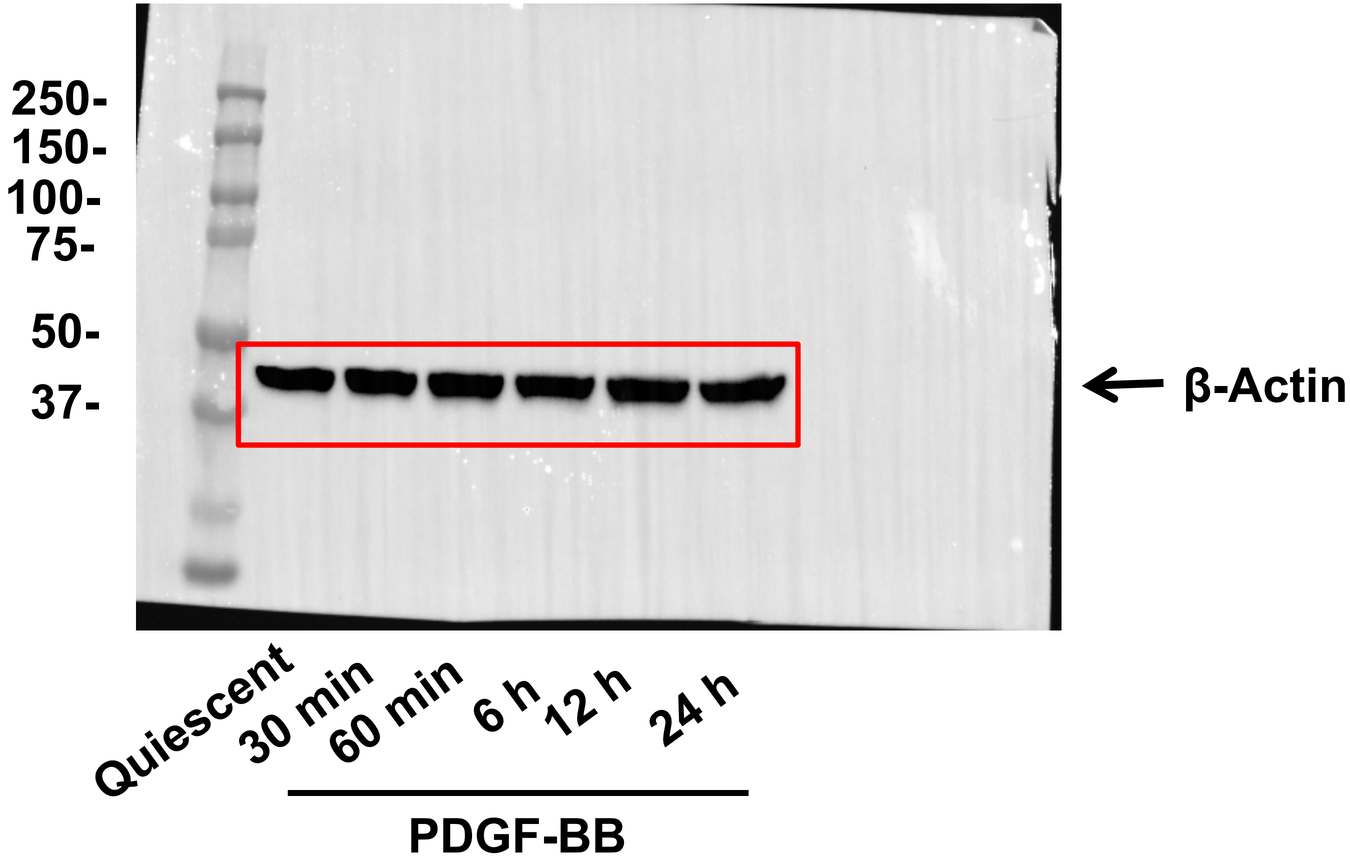

Full unedited gel for Figure 1D-SM22α

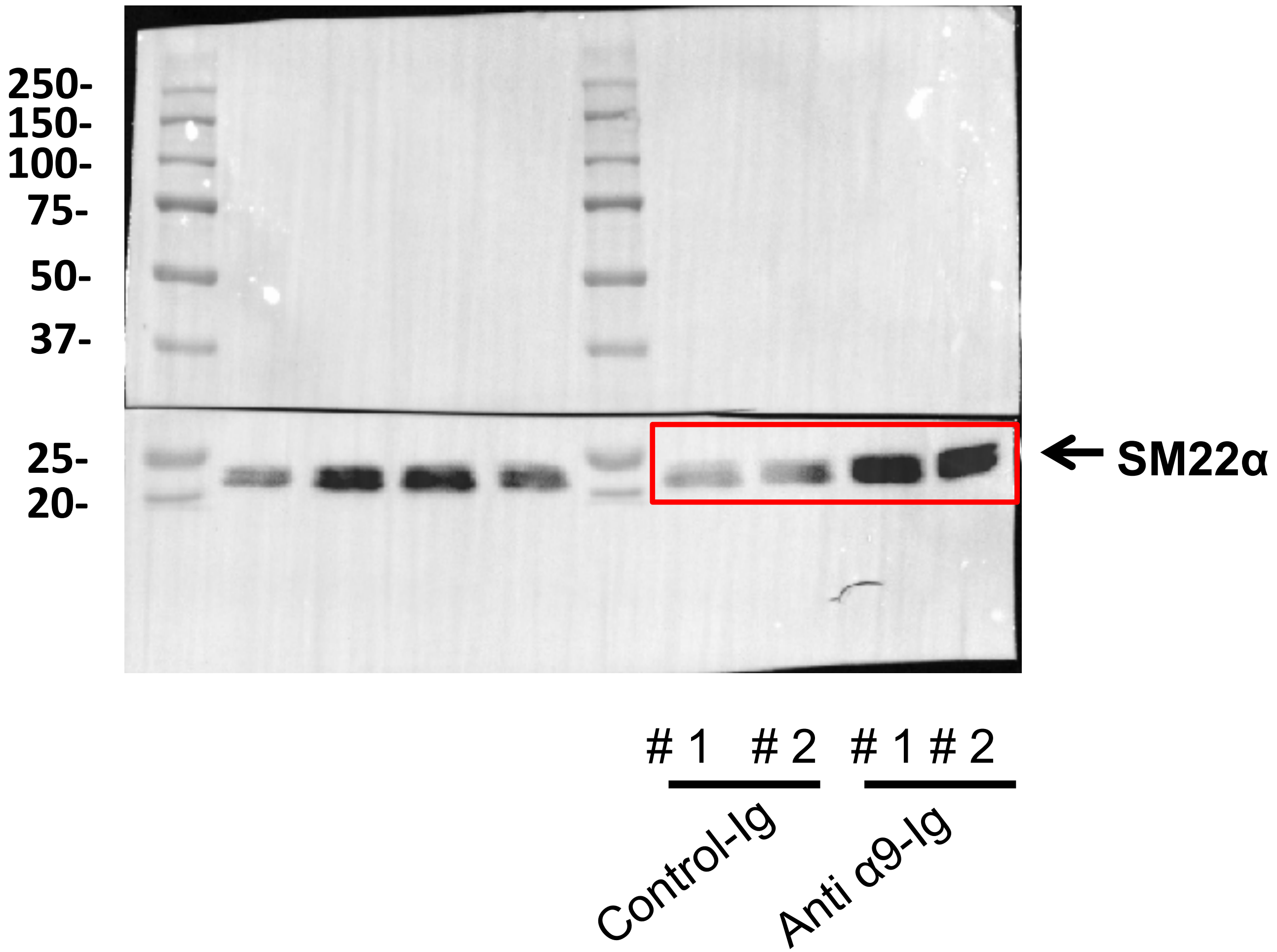

Full unedited gel for Figure 1D-Vimentin

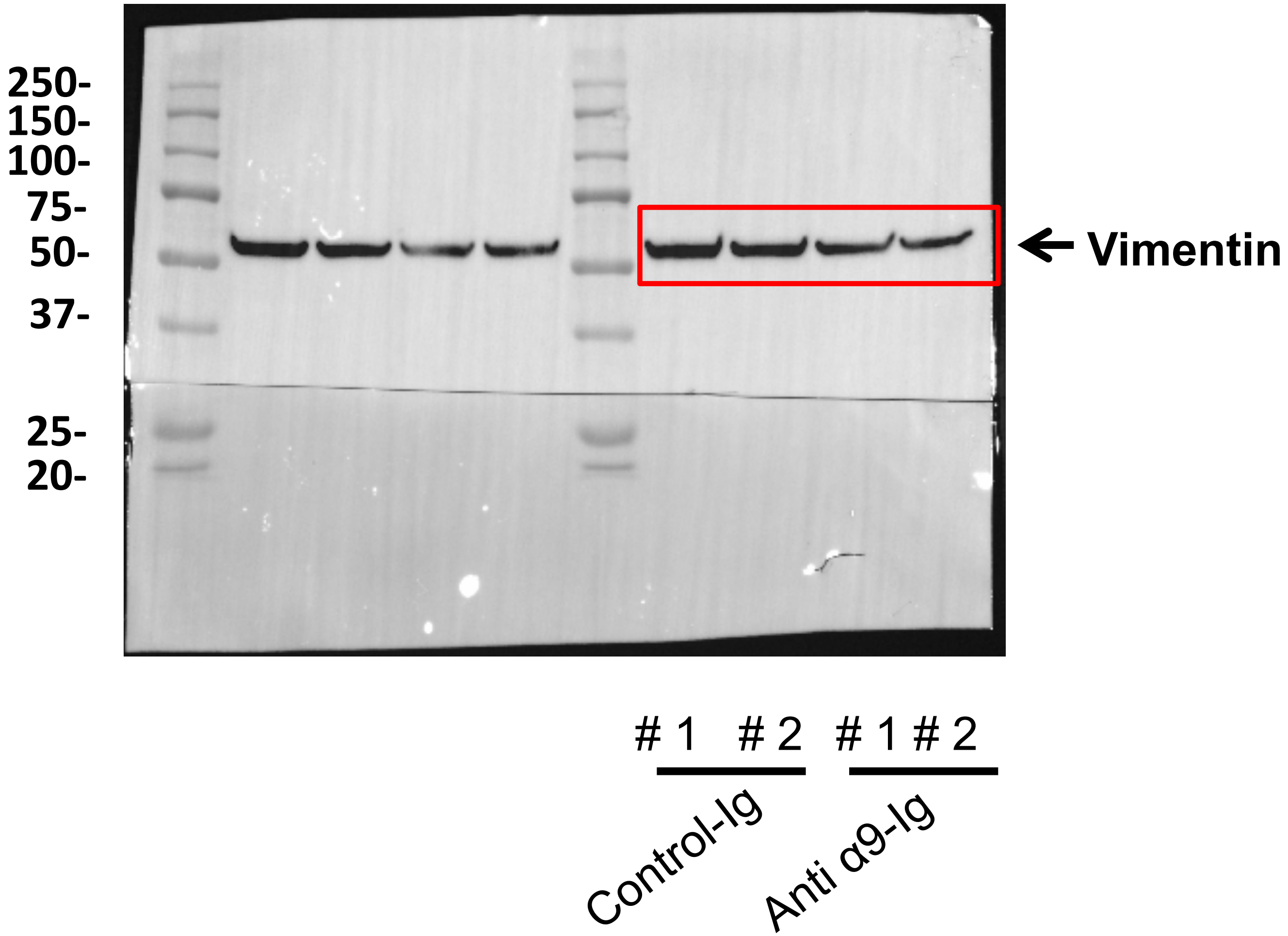

Full unedited gel for Figure 1D-SM-MHC

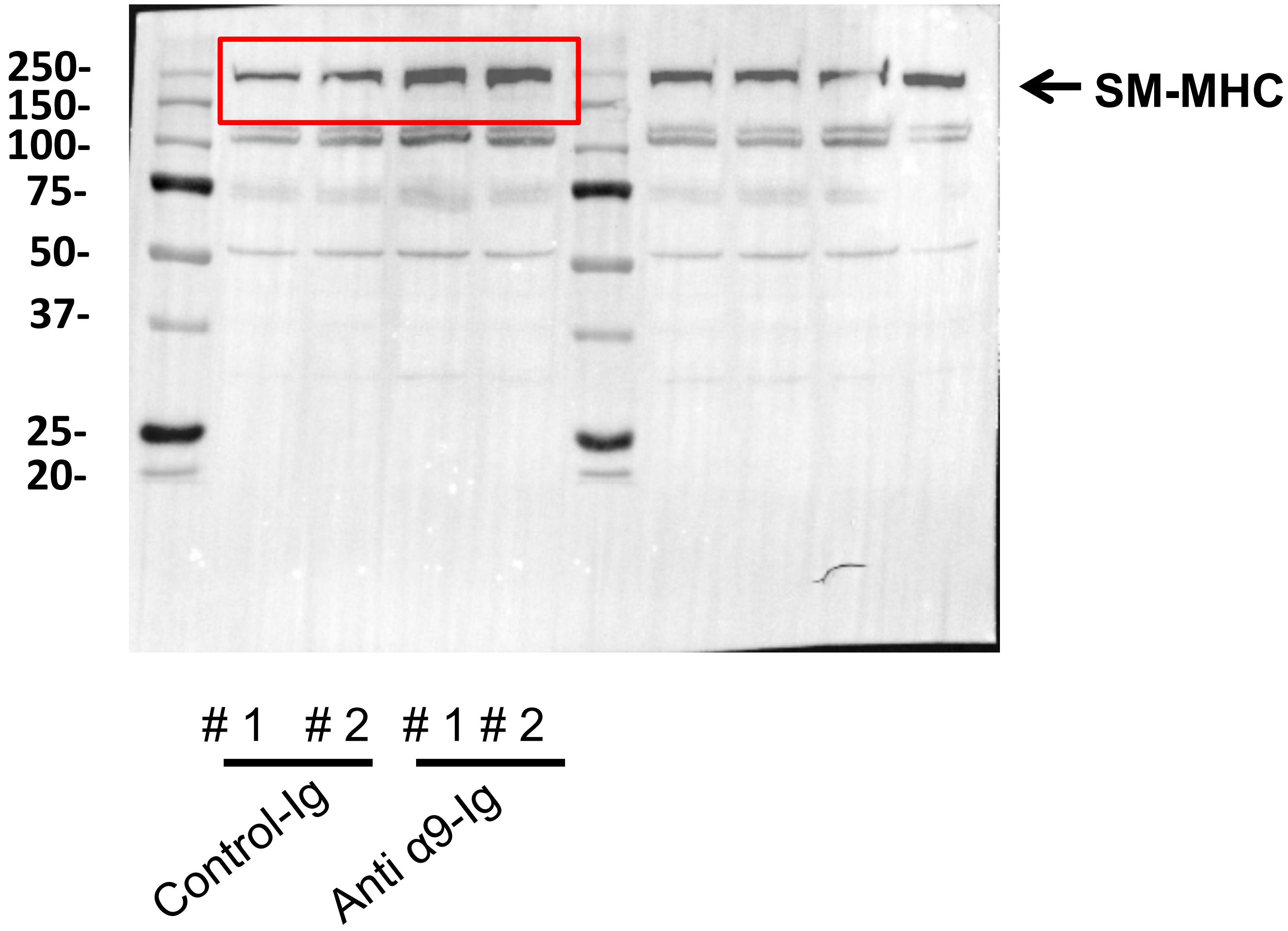

Full unedited gel for Figure 1D-β-Actin

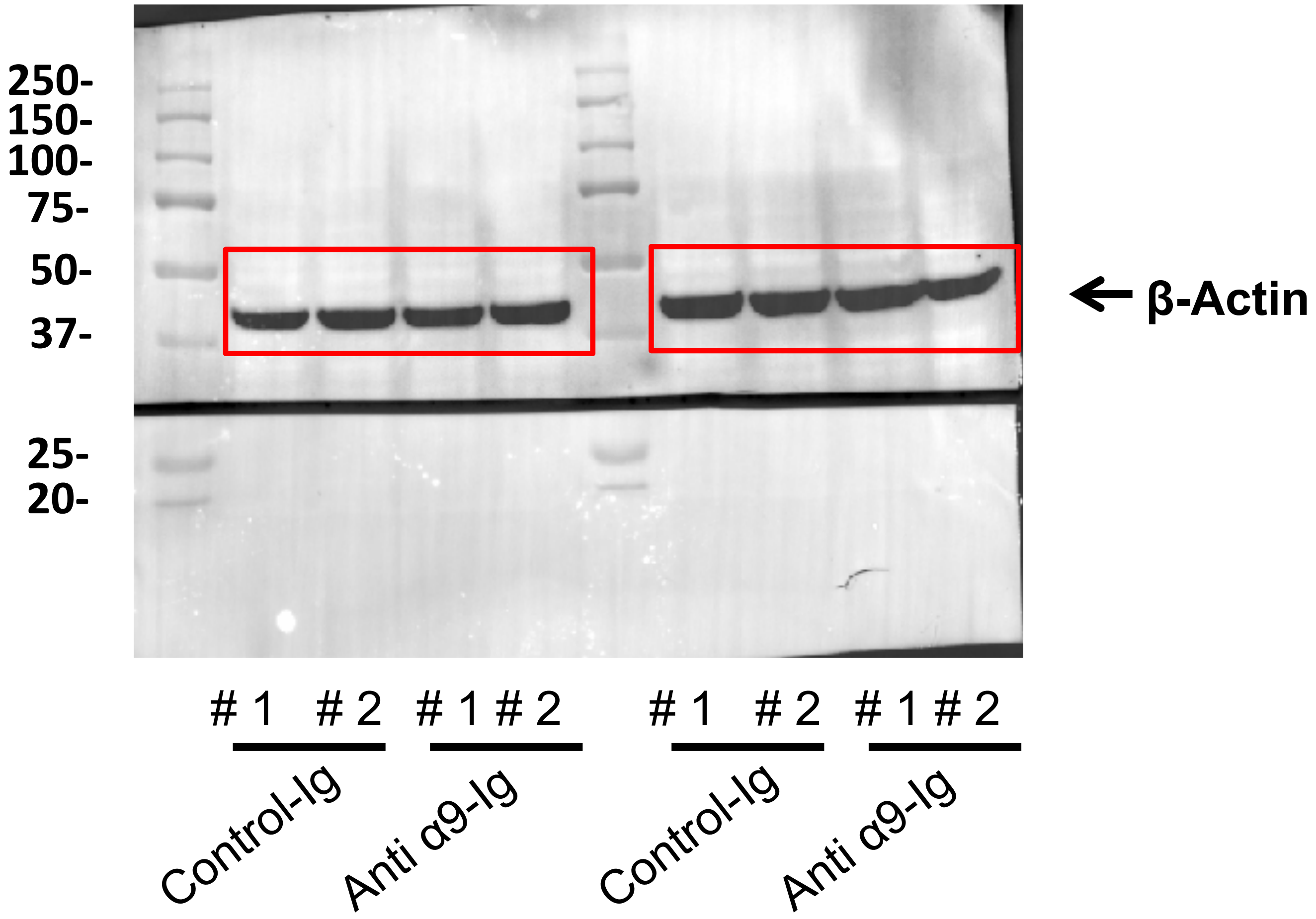

Full unedited gel for Figure 1D-Osteopontin

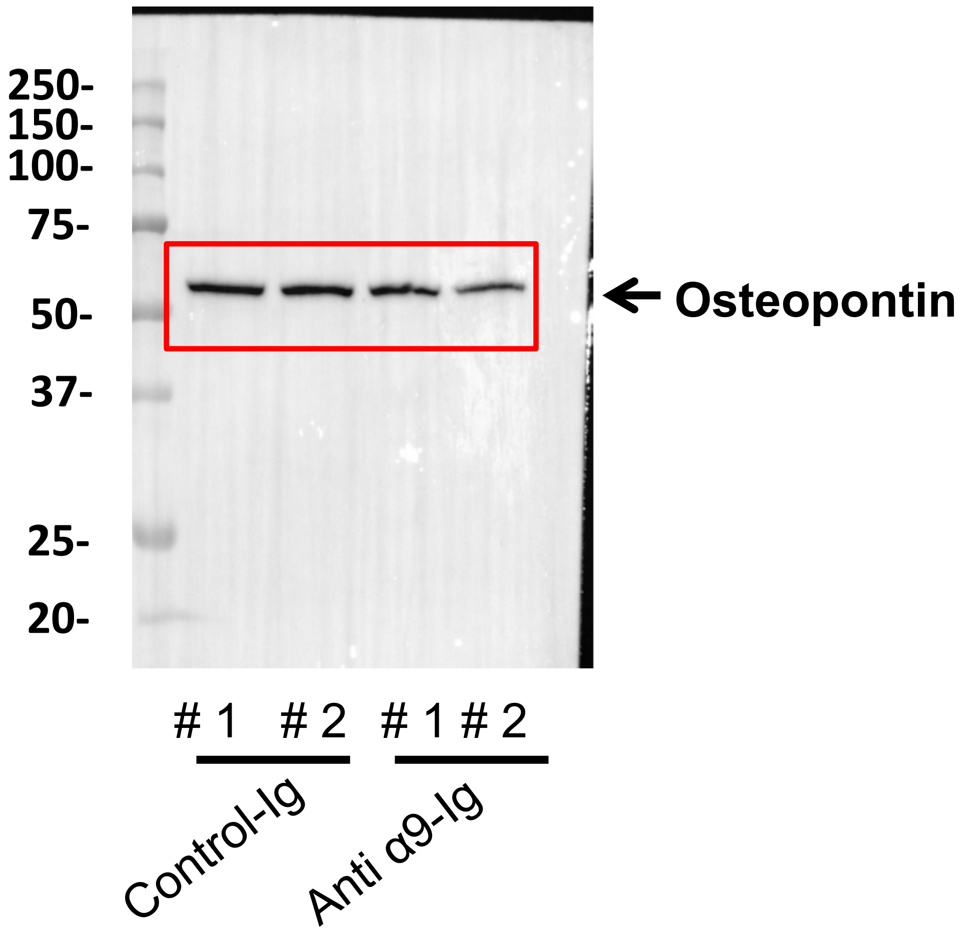

Full unedited gel for Figure 1D-β-Actin

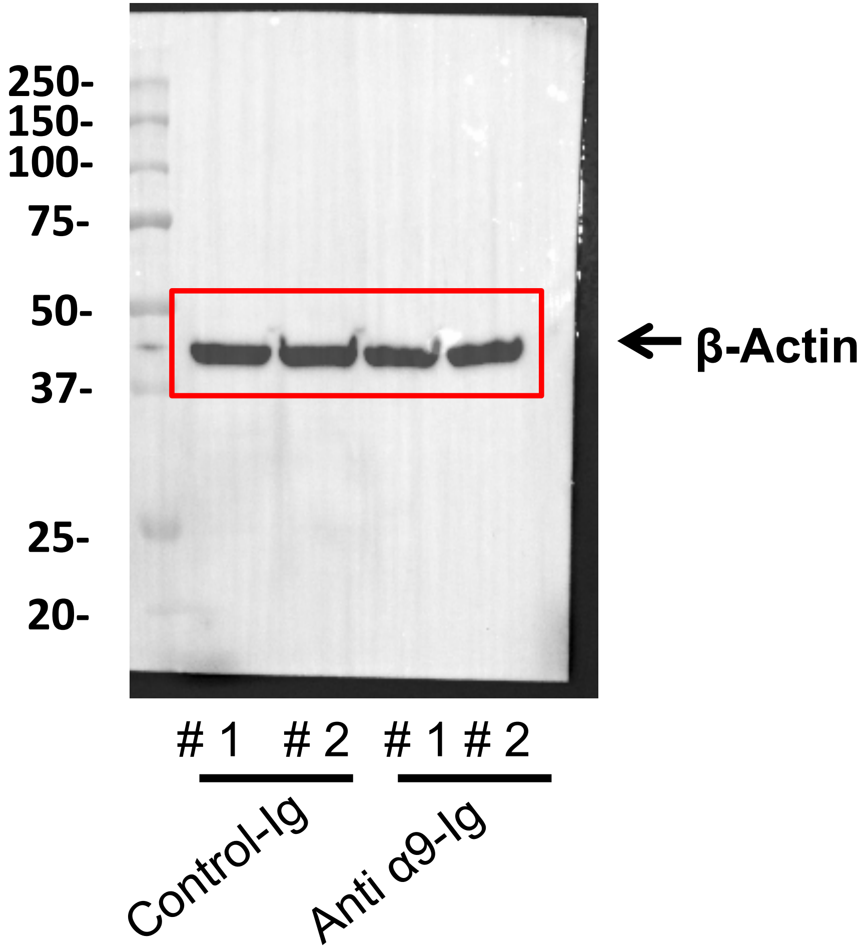

Full unedited gel for Figure 2A-p-FAK

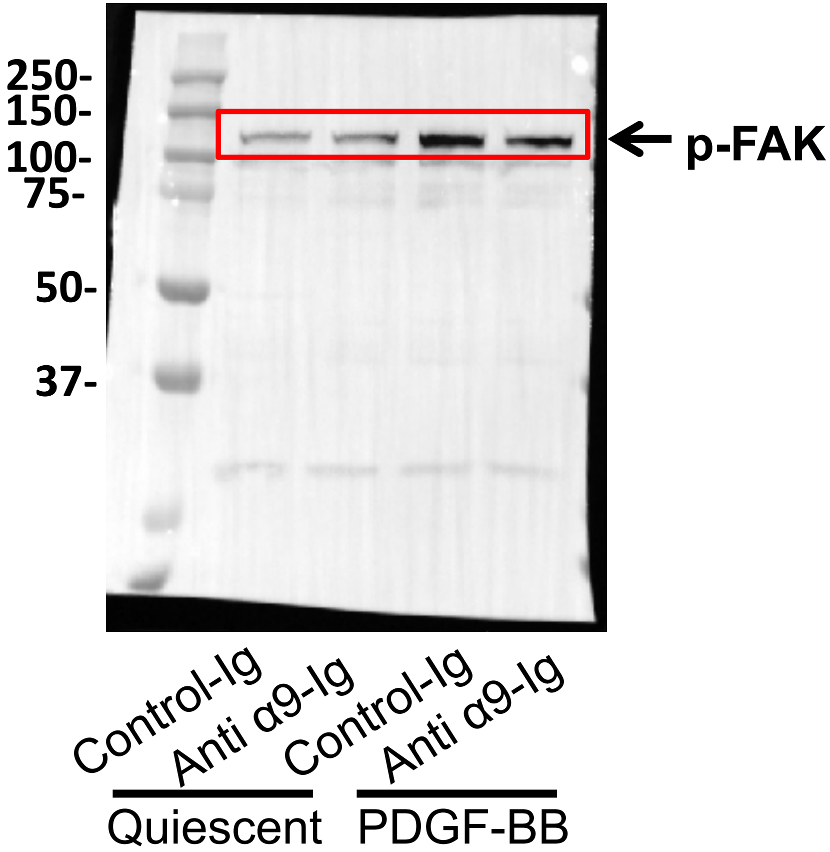

Full unedited gel for Figure 2A-FAK

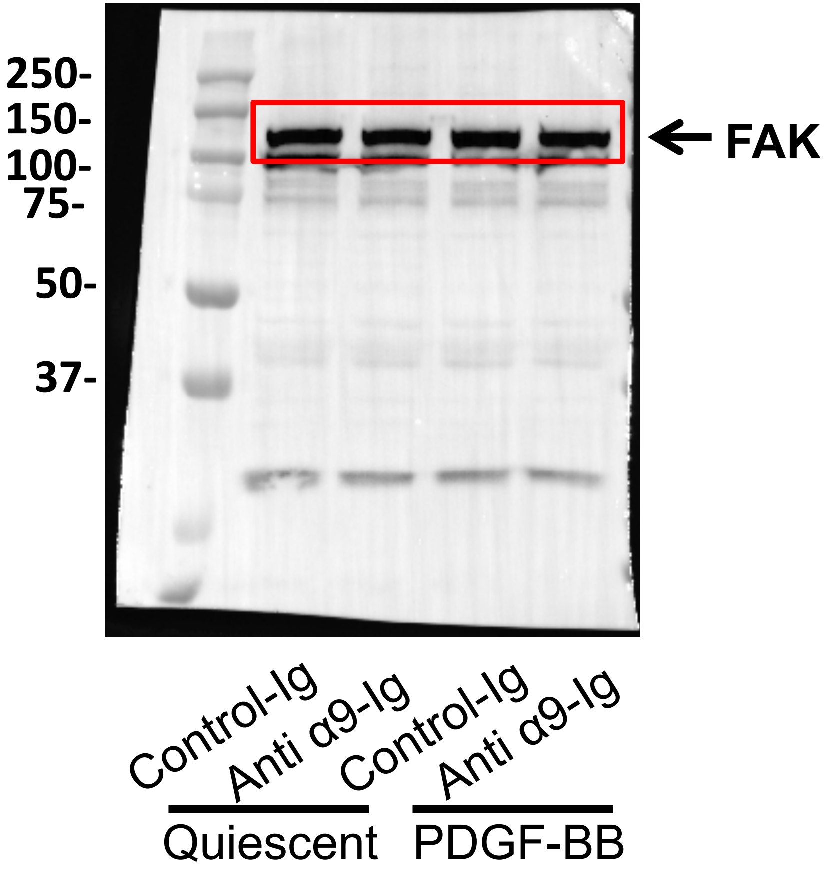

Full unedited gel for Figure 2A-p-Src

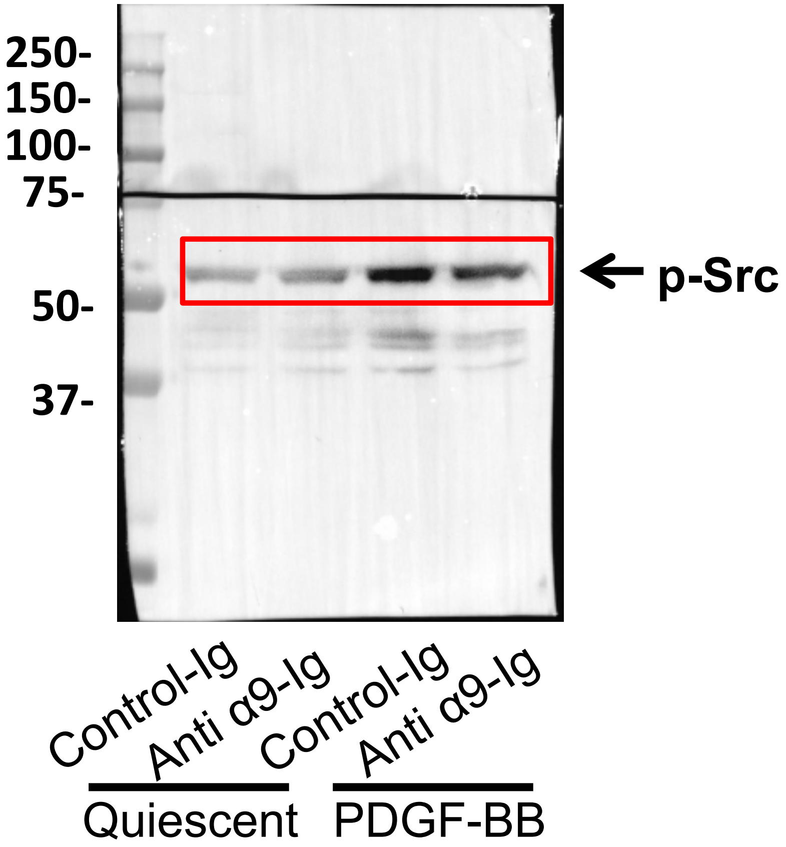

Full unedited gel for Figure 2A-Src

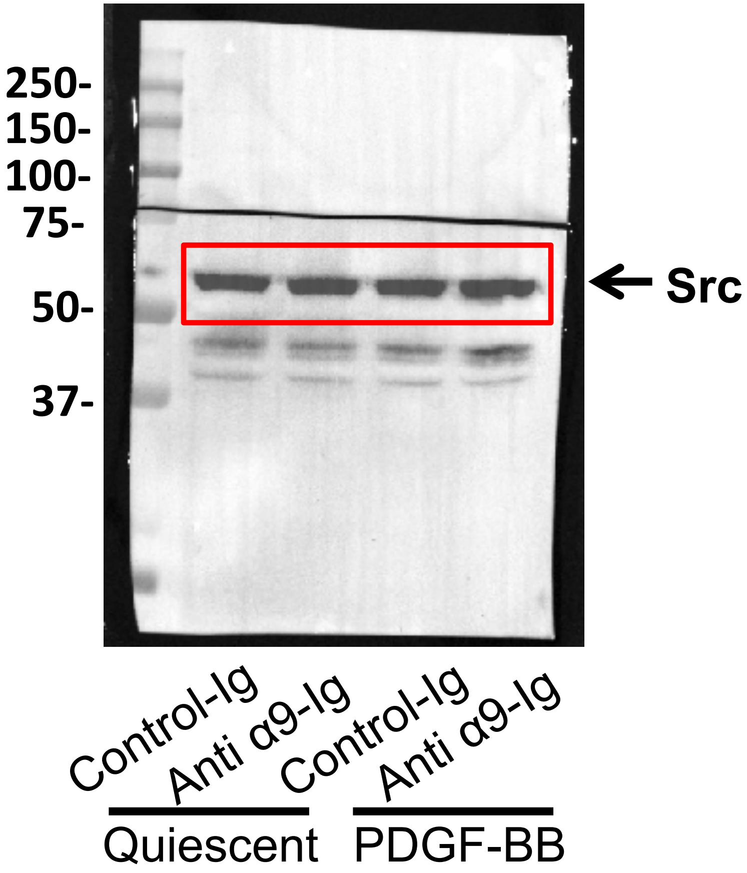

Full unedited gel for Figure 2A-pERK1/2

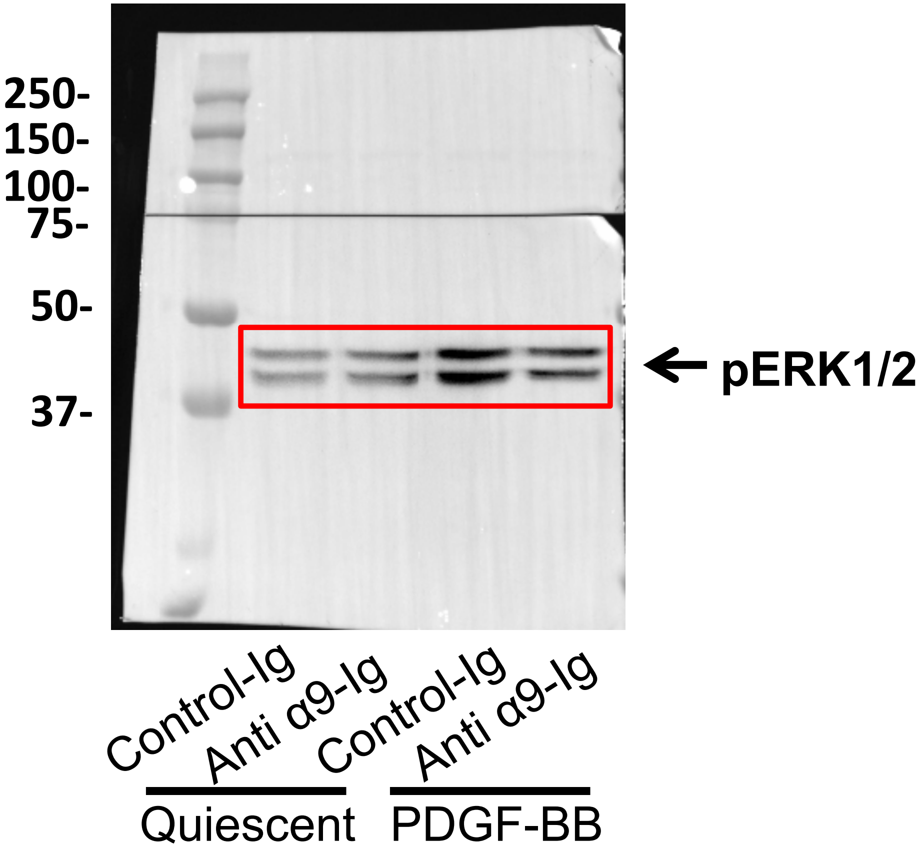

Full unedited gel for Figure 2A-ERK1/2

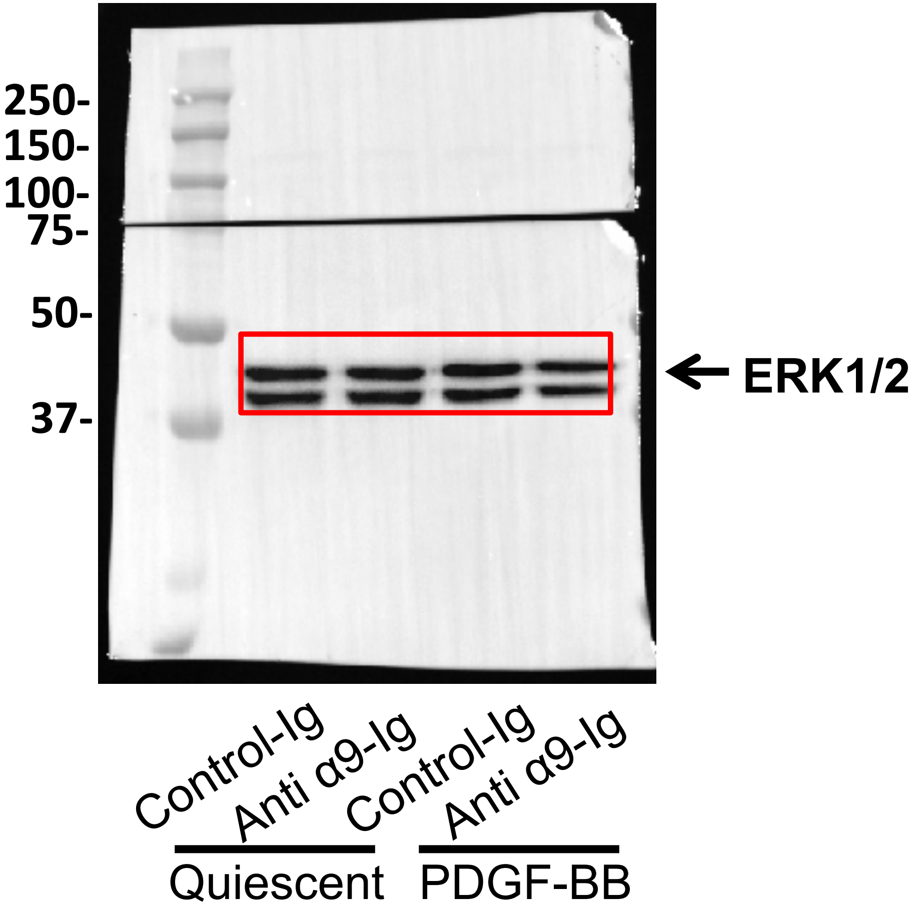

Full unedited gel for Figure 2A-p-p38

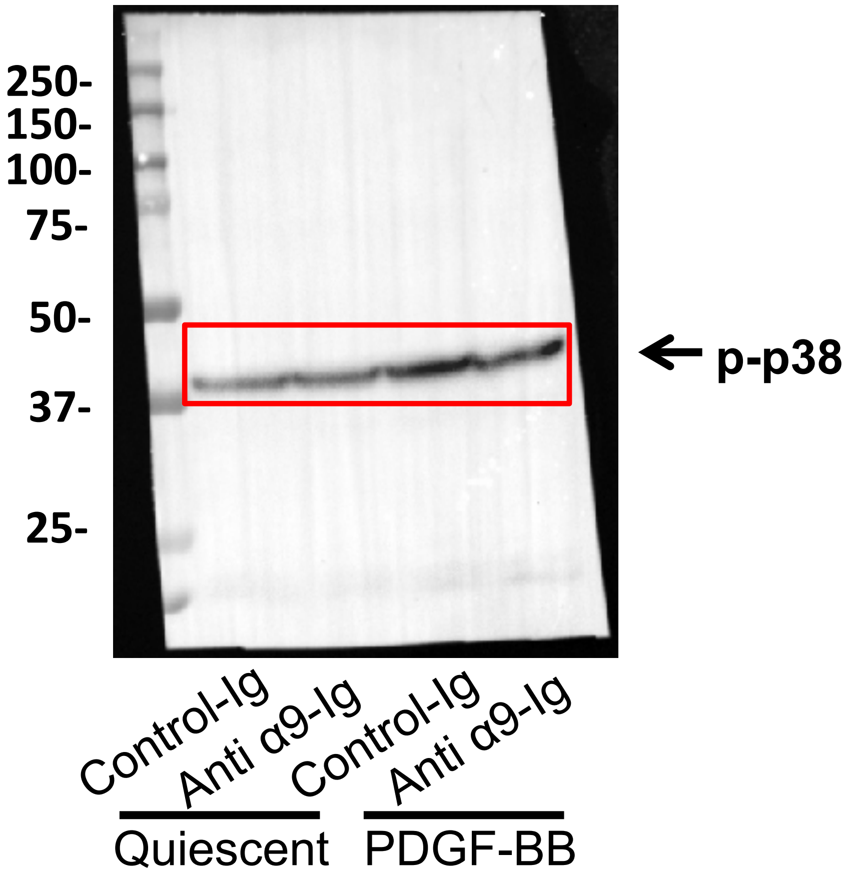

Full unedited gel for Figure 2A-p38

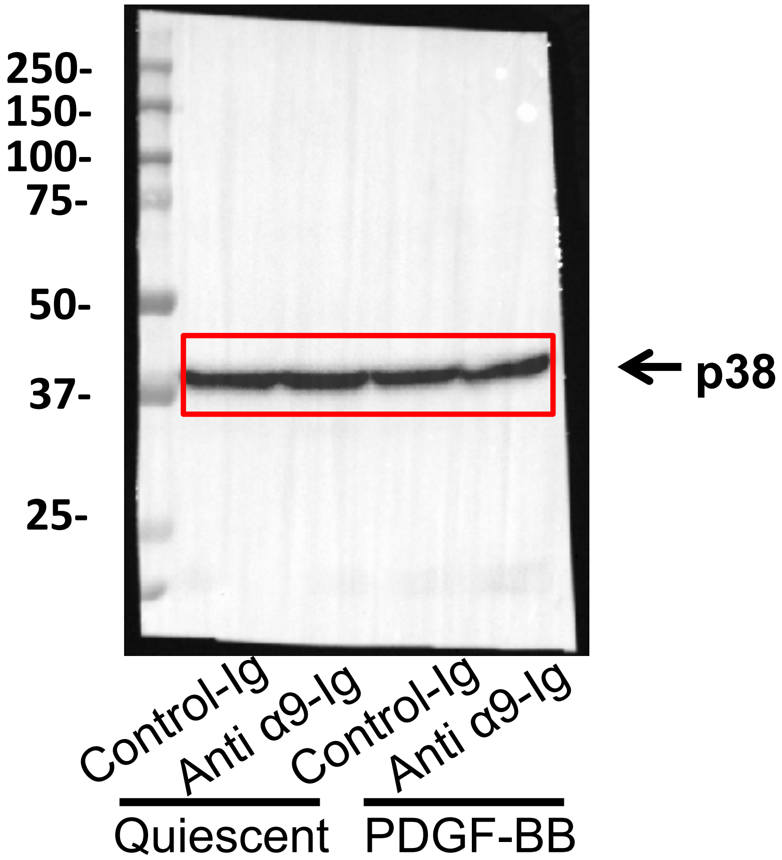

Full unedited gel for Figure 2B-pGSK3β

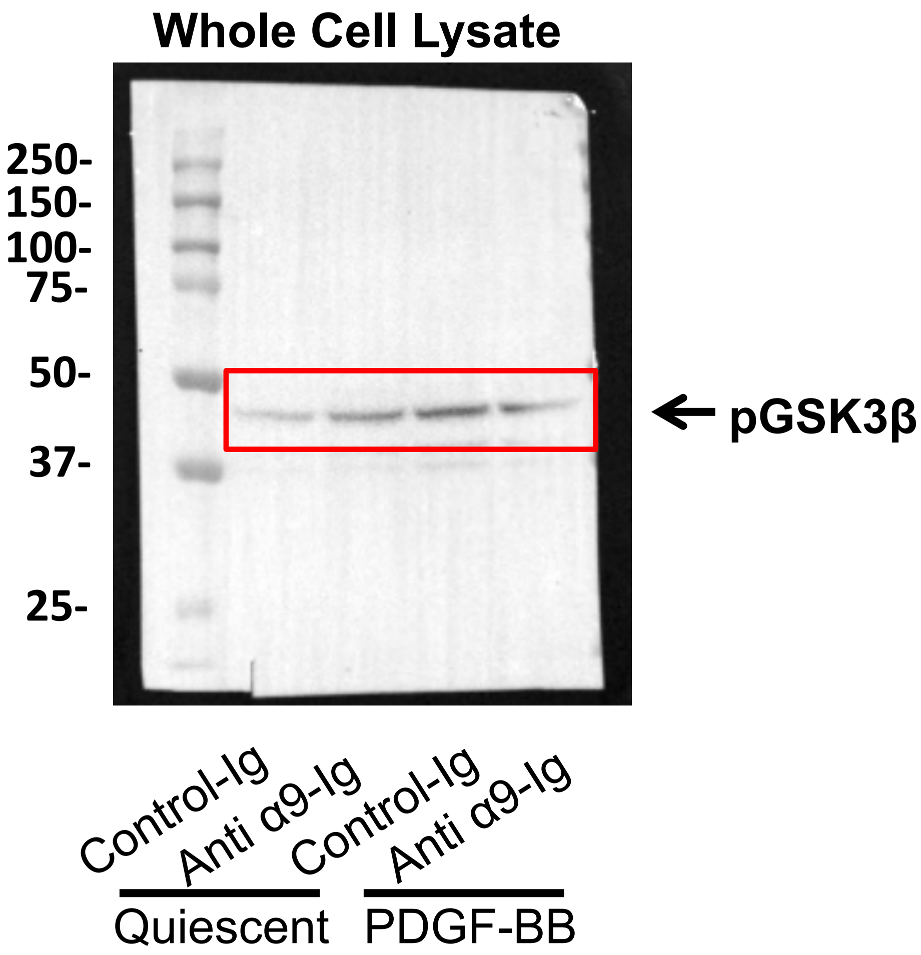

Full unedited gel for Figure 2B-GSK3β

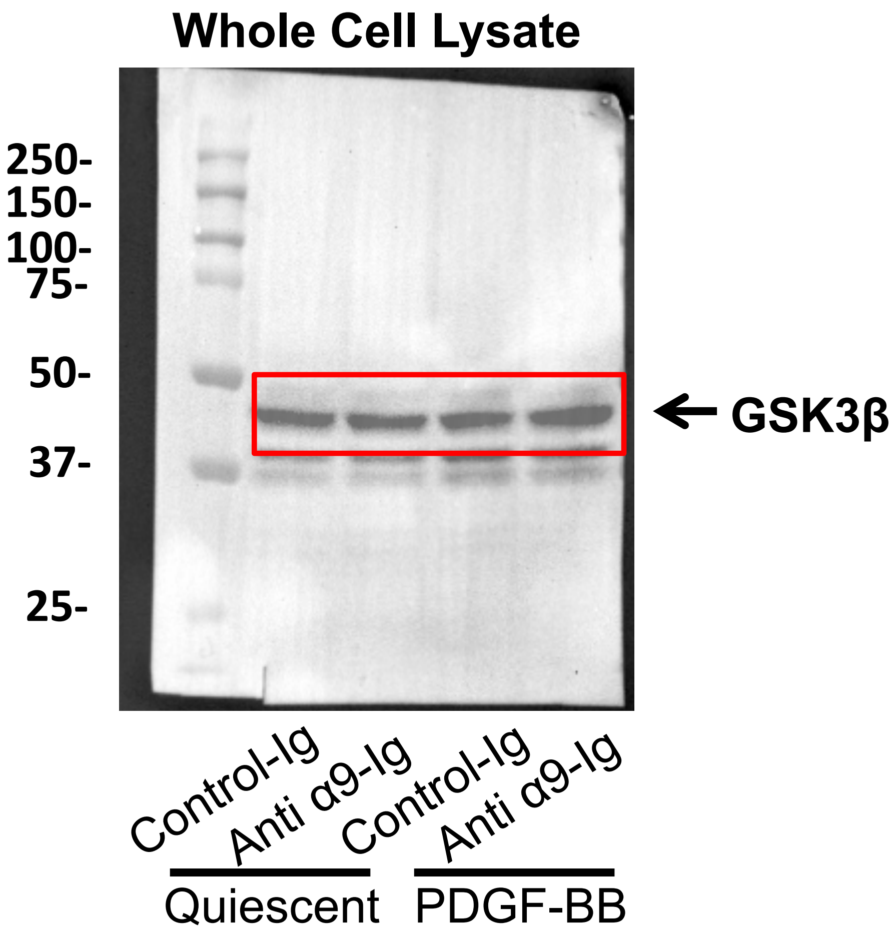

Full unedited gel for Figure 2B-Active β-catenin

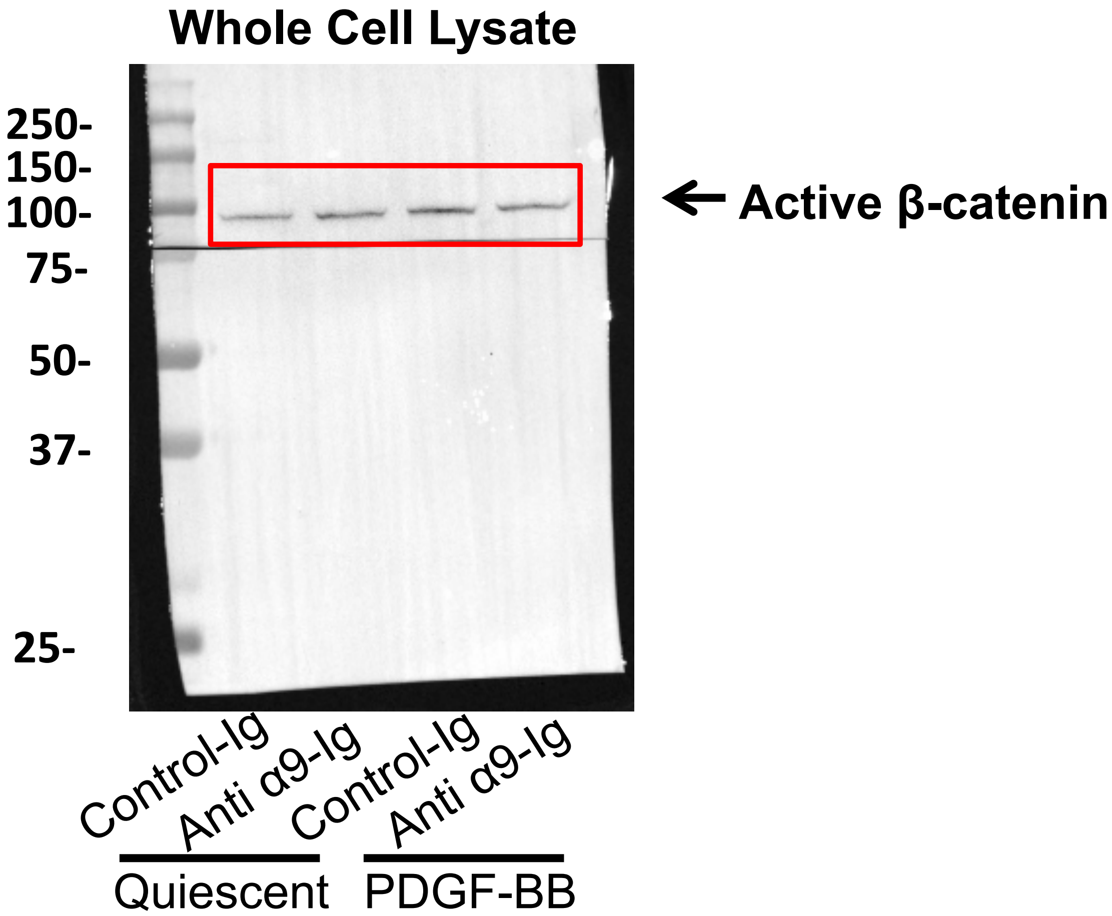

Full unedited gel for Figure 2B- Total β-catenin

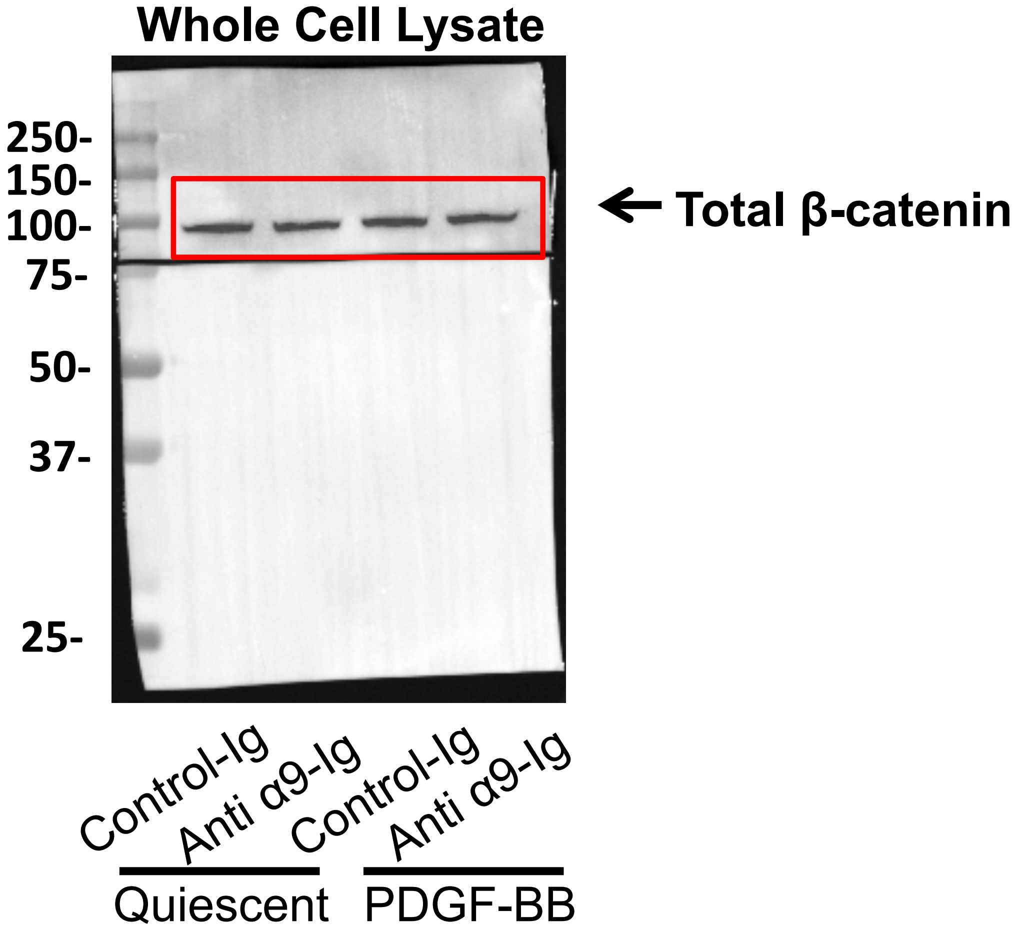

Full unedited gel for Figure 2B- β-Actin

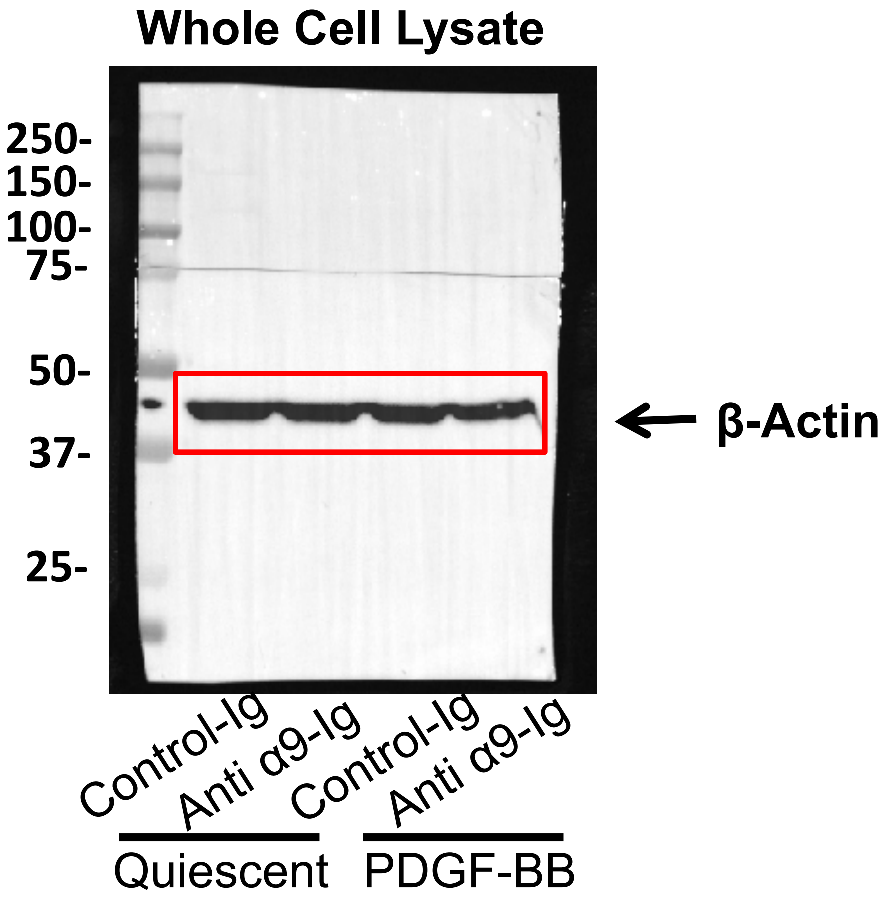

Full unedited gel for Figure 2B- β-catenin

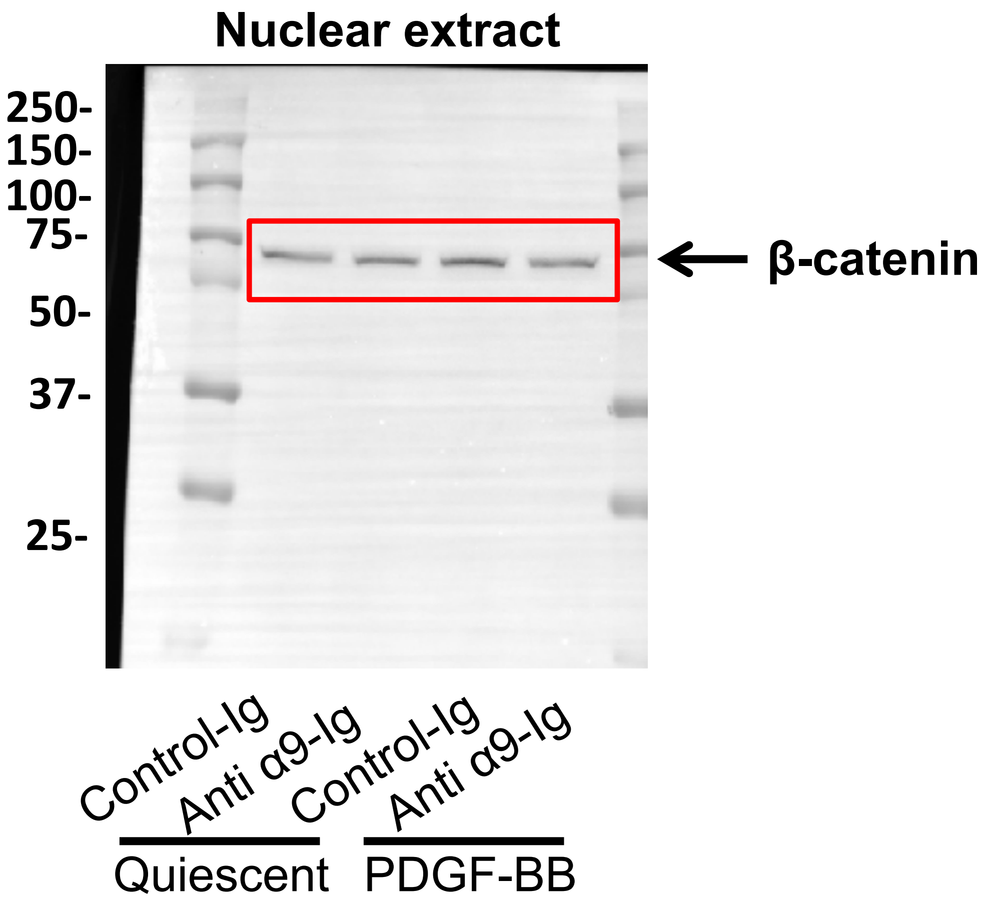

Full unedited gel for Figure 2B- Lamin B1

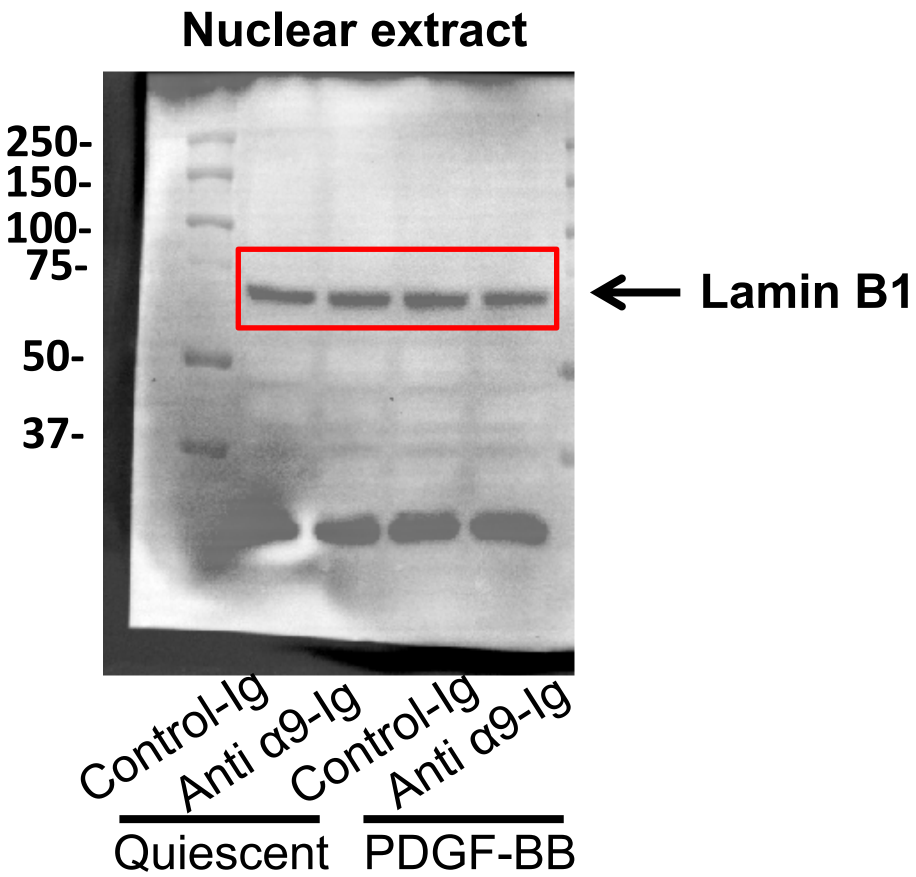

Full unedited gel for Figure 2B- GAPDH

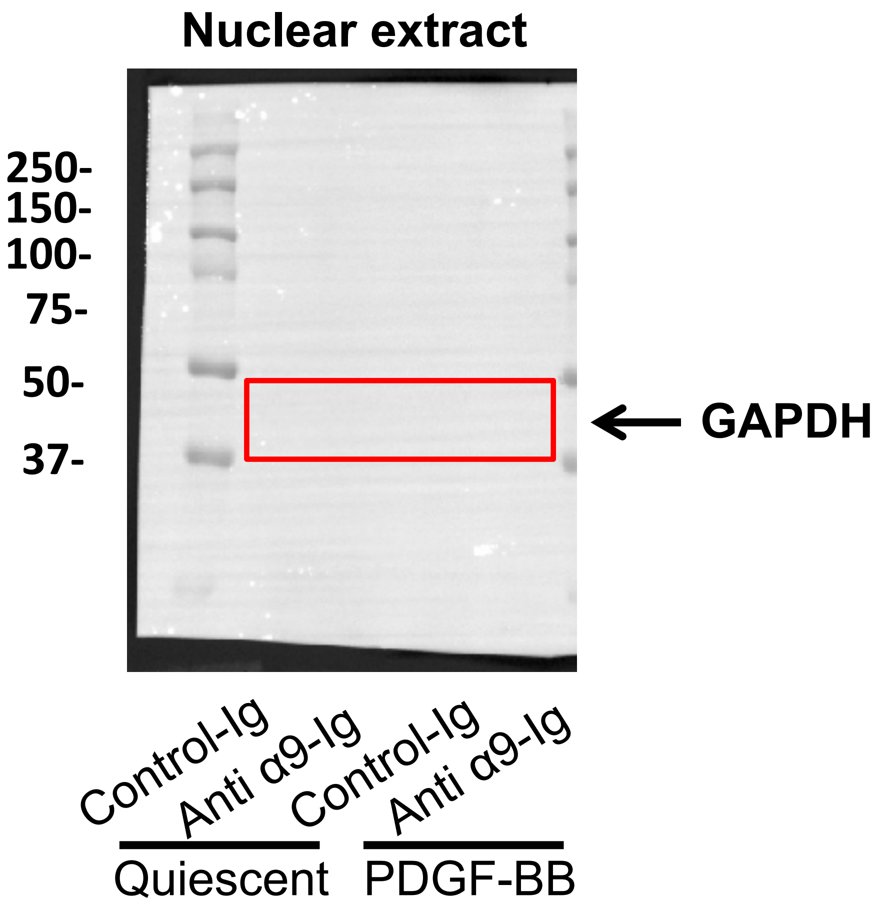

Full unedited gel for Figure 3A- Integrin α9

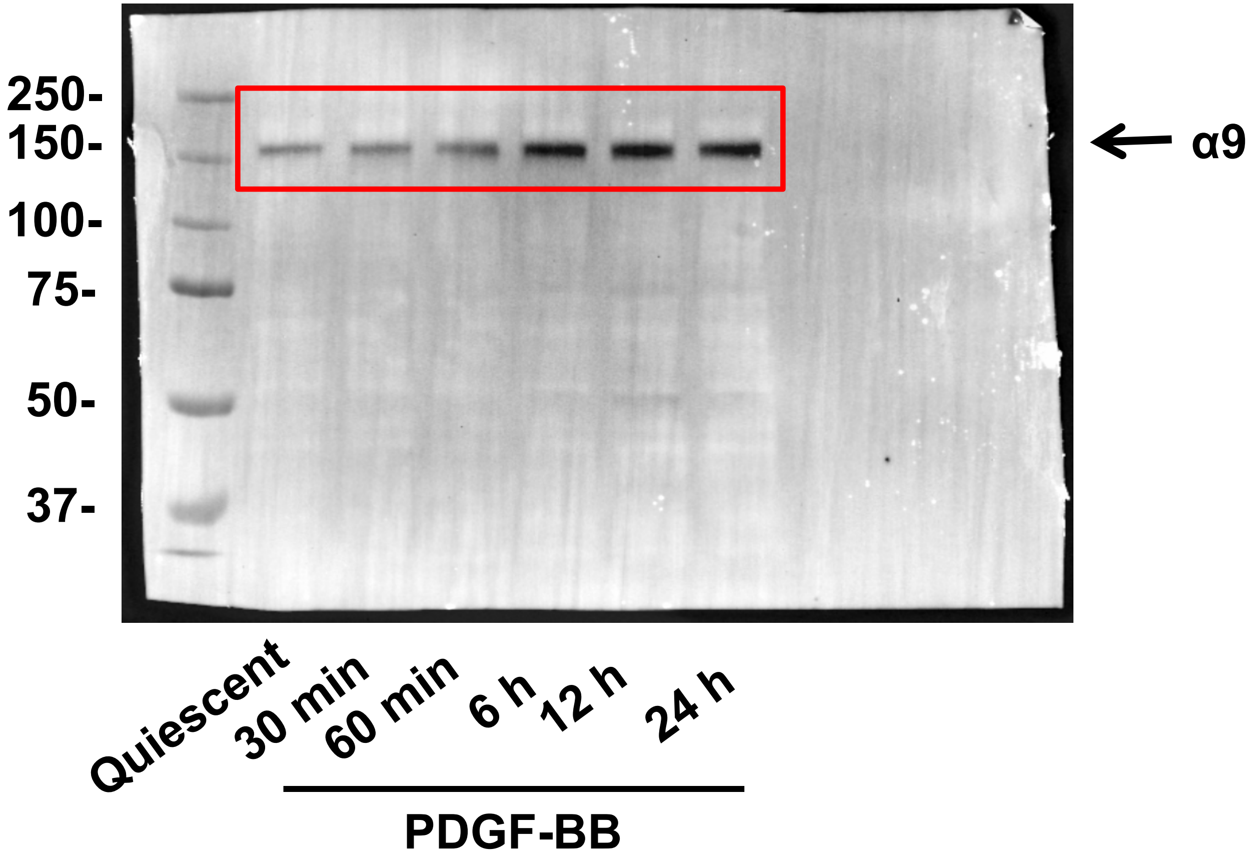

Full unedited gel for Figure 3A- β-Actin

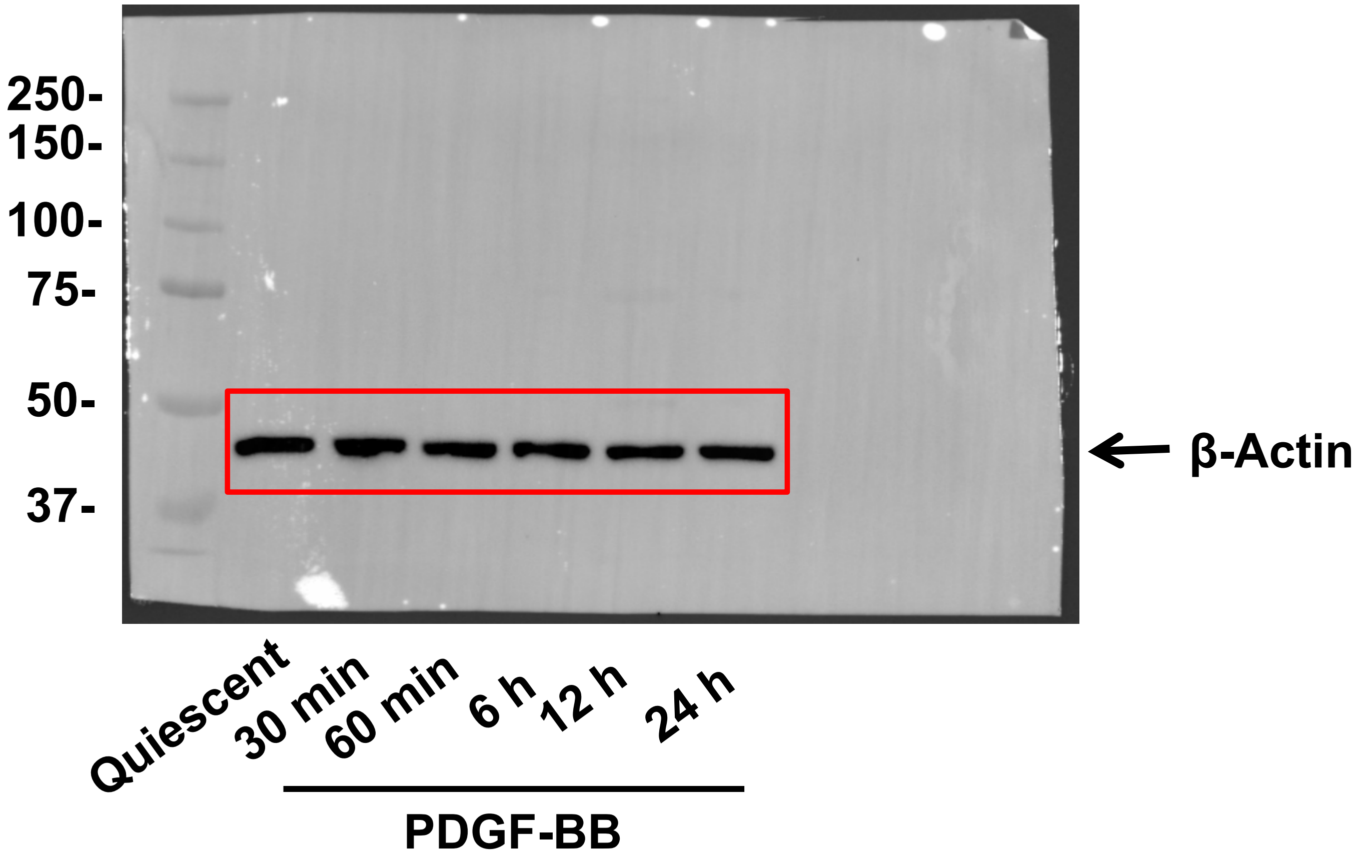

Full unedited gel for Figure 3C- Integrin α9

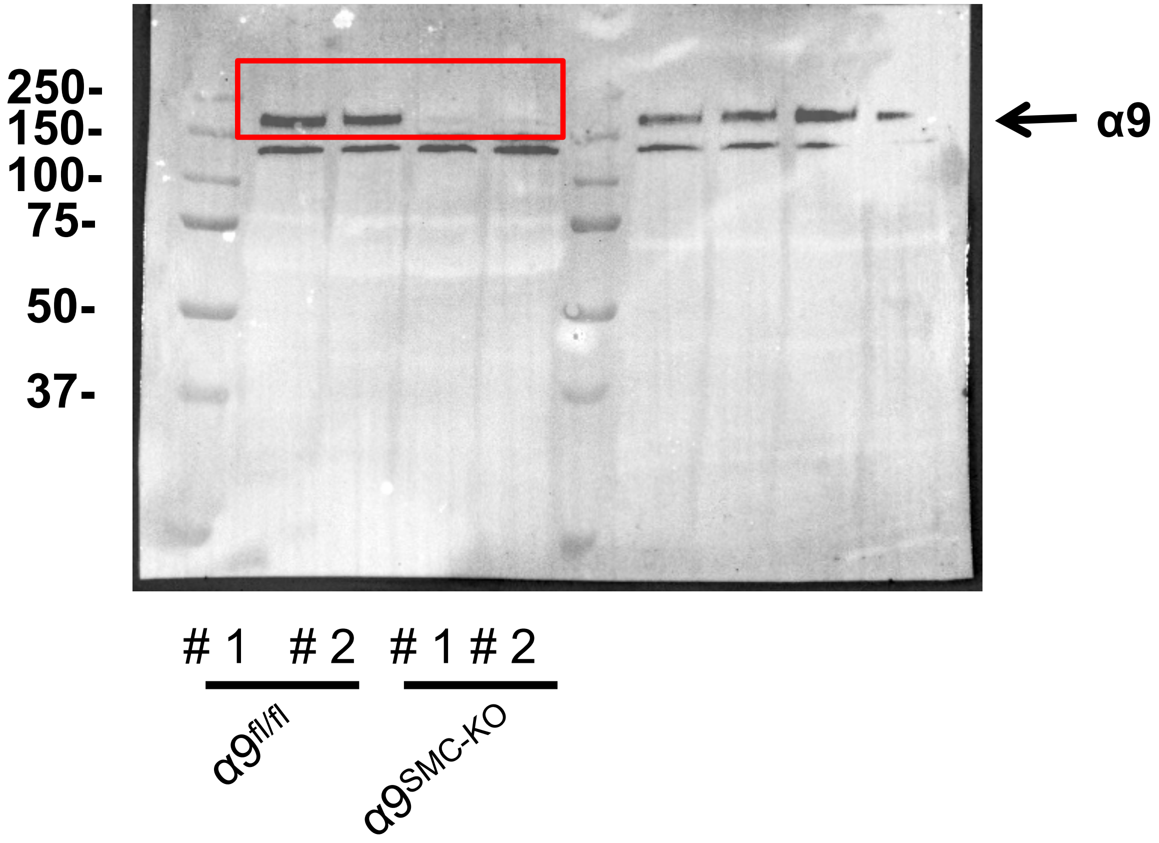

Full unedited gel for Figure 3C- β-Actin

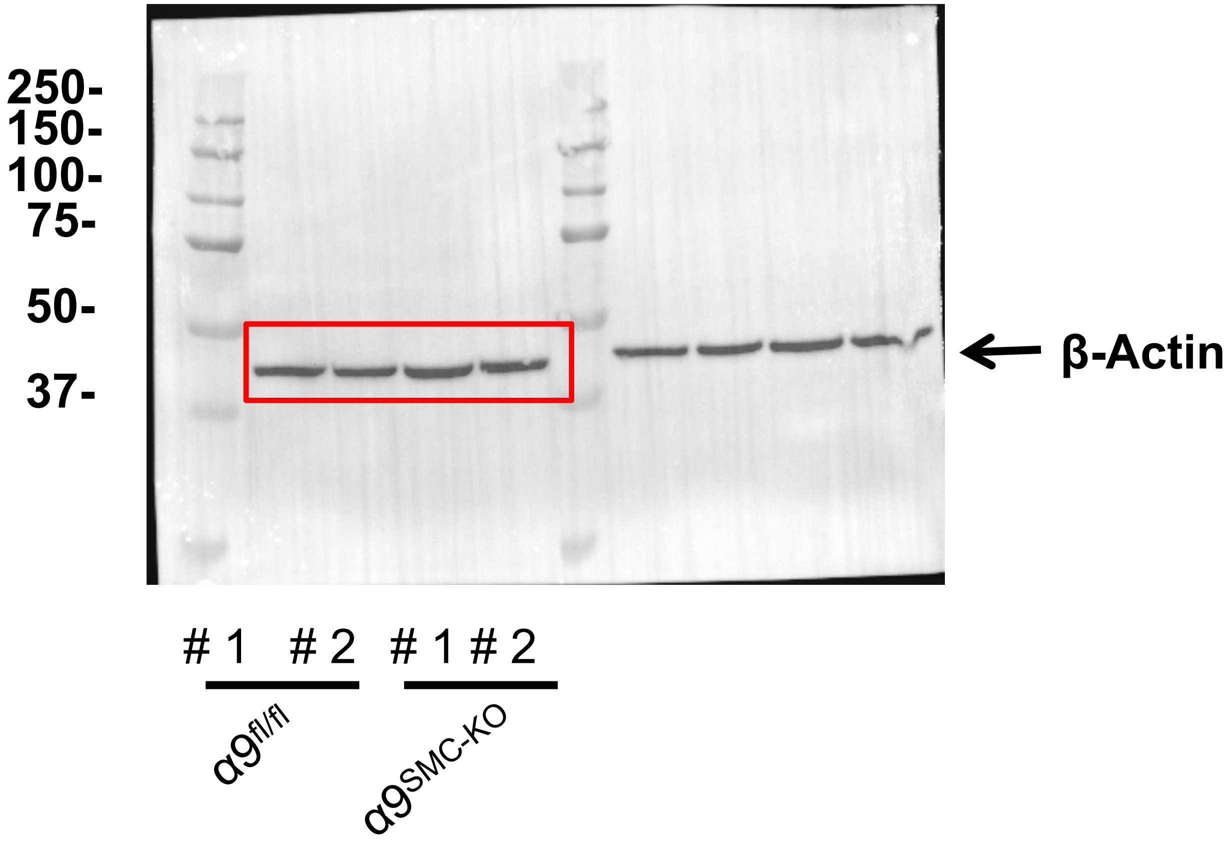

Full unedited gel for Figure 4B-SM22α

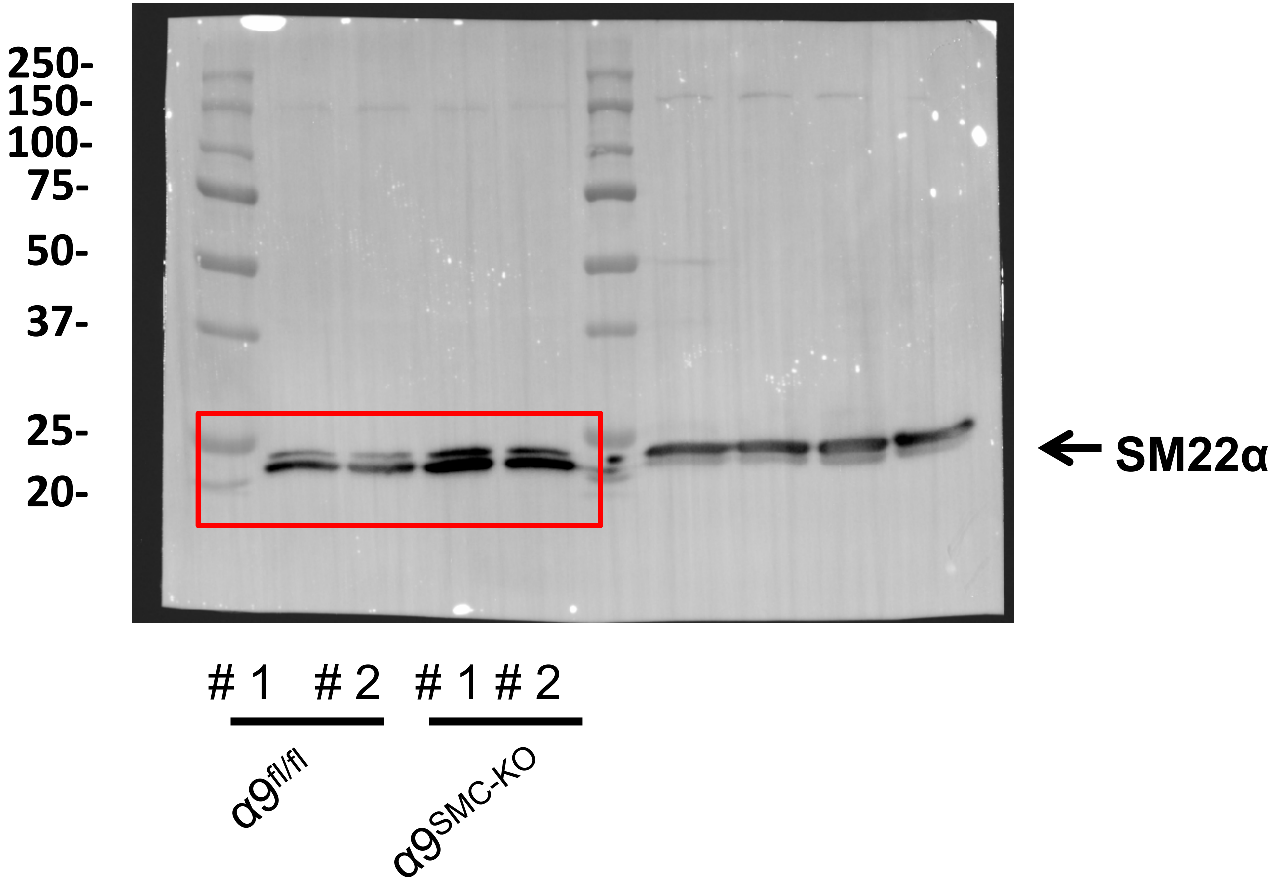

Full unedited gel for Figure 4B-SM-MHC

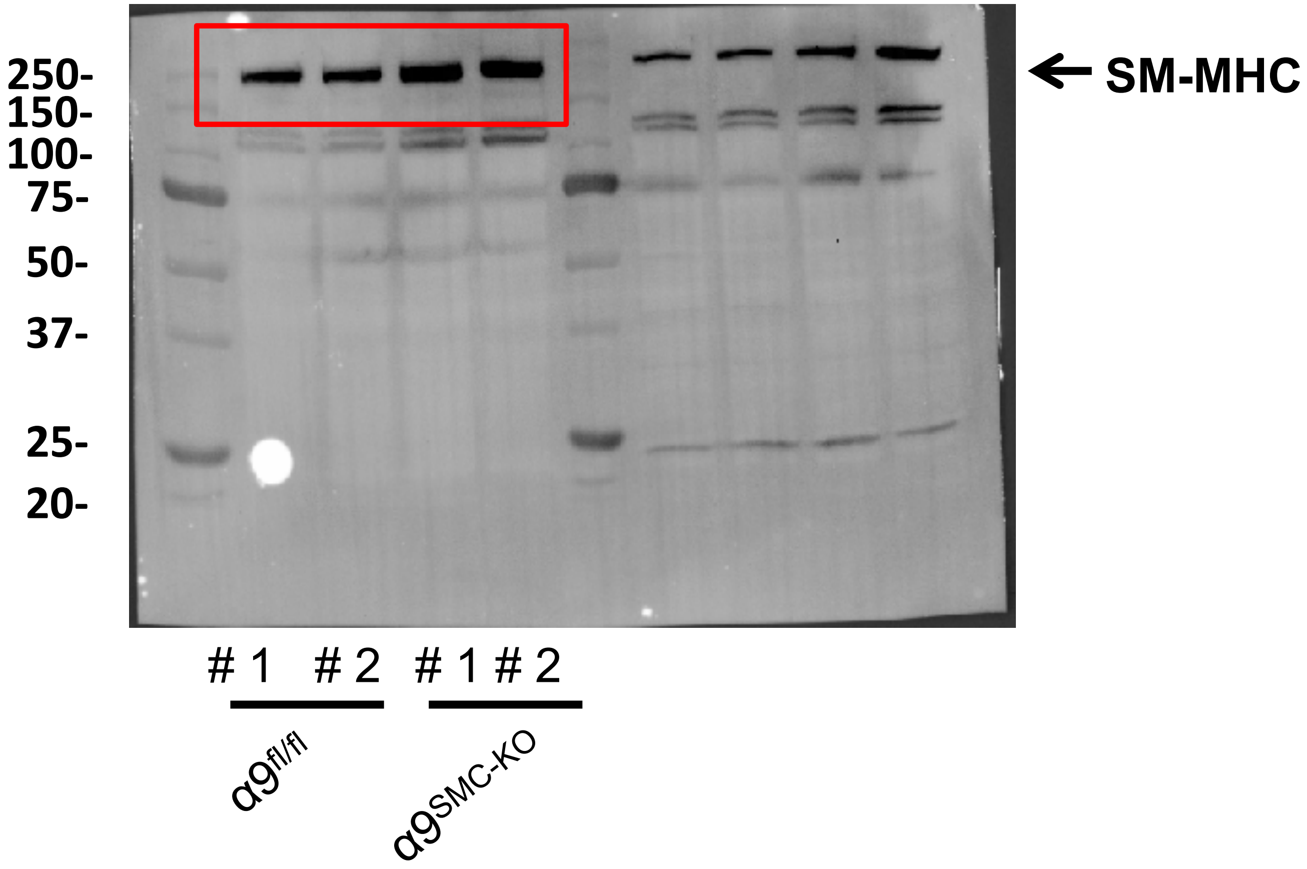

Full unedited gel for Figure 4B-Vimentin

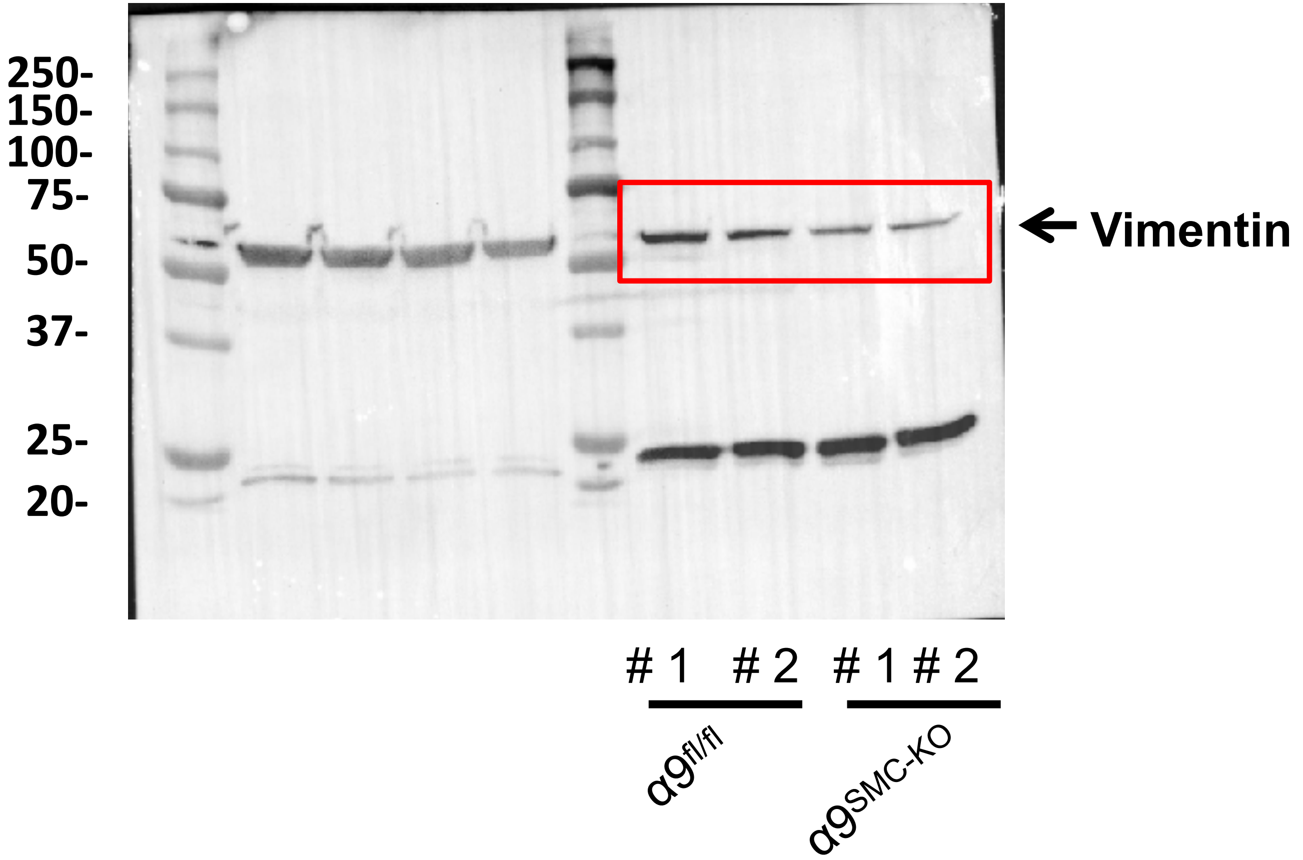

Full unedited gel for Figure 4B-β-Actin

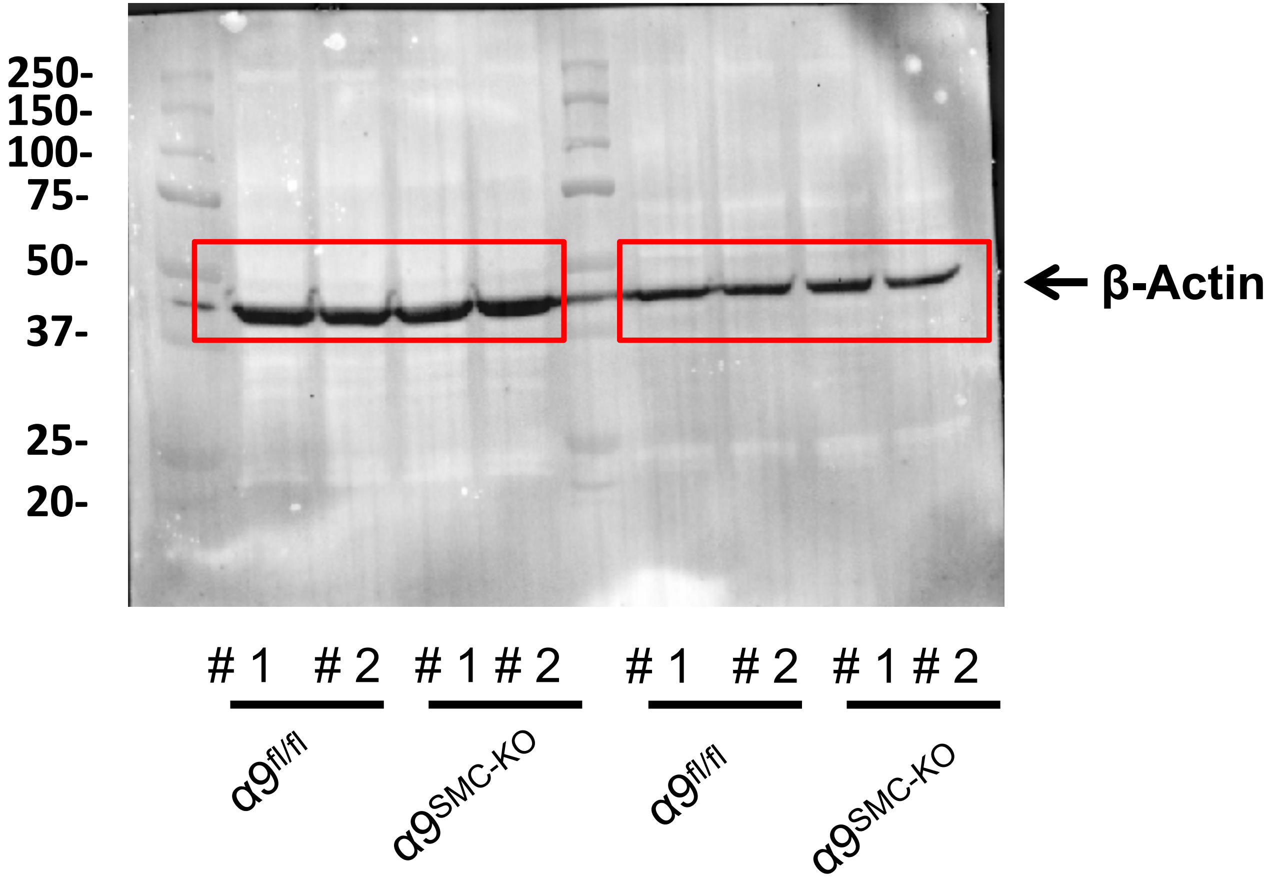

Full unedited gel for Figure 4B-Osteopontin

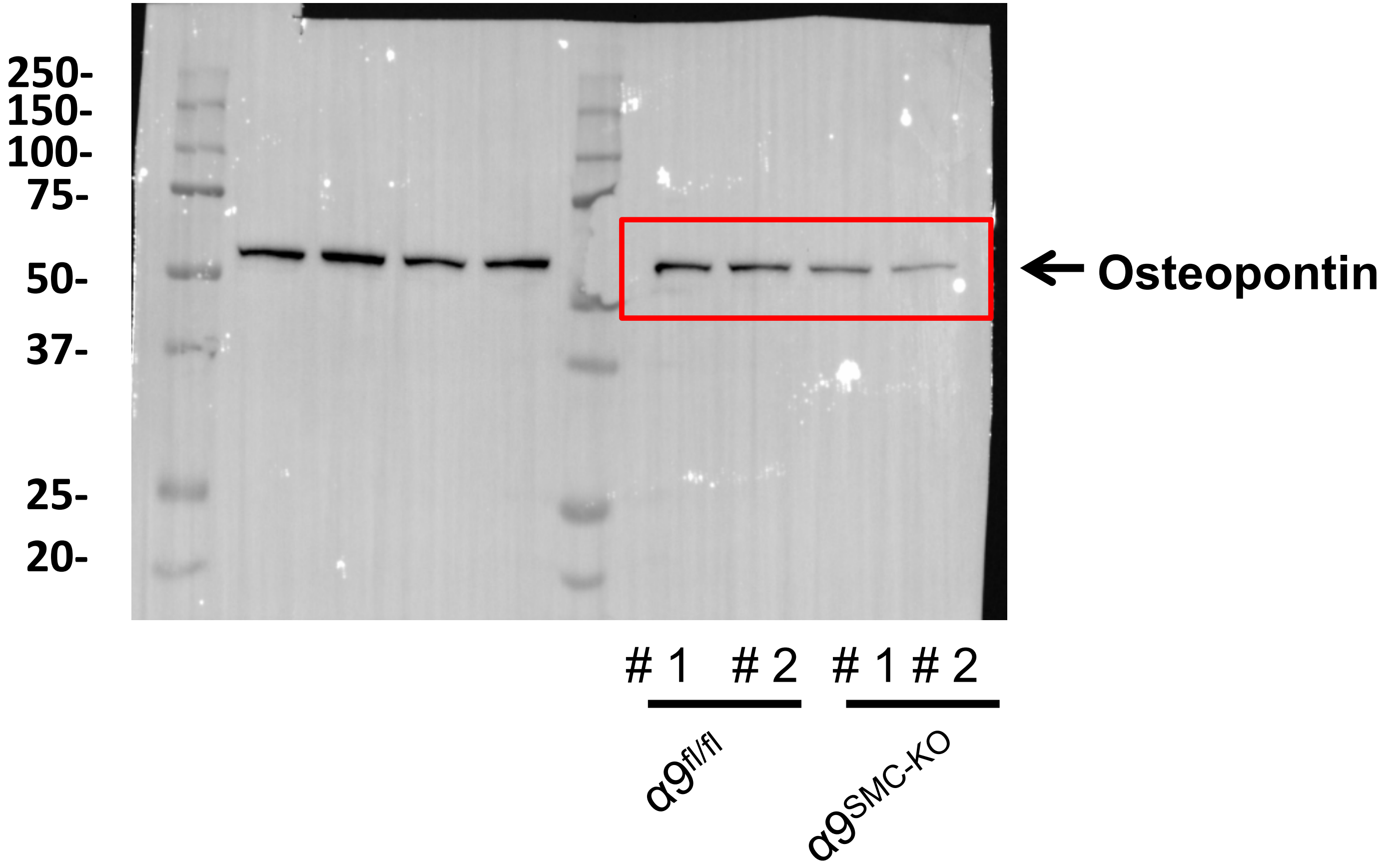

Full unedited gel for Figure 4B-β-Actin

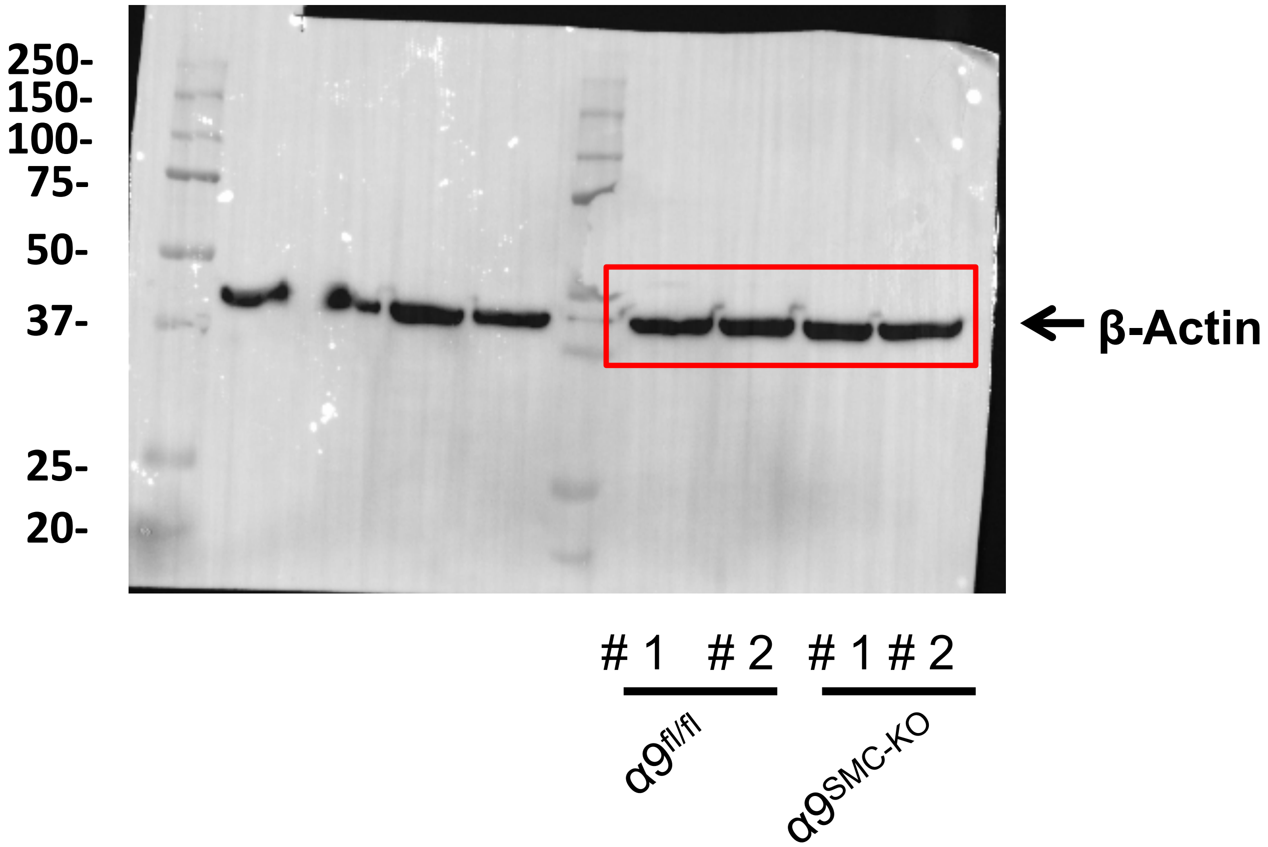

Full unedited gel for Figure 5A-p-FAK

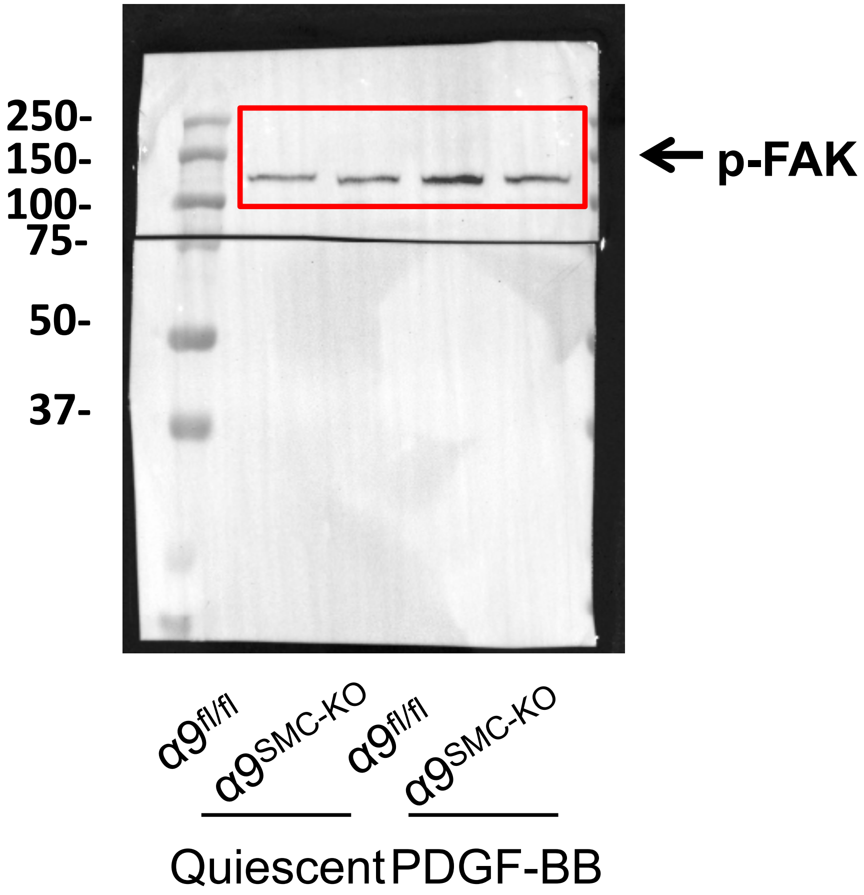

Full unedited gel for Figure 5A-FAK

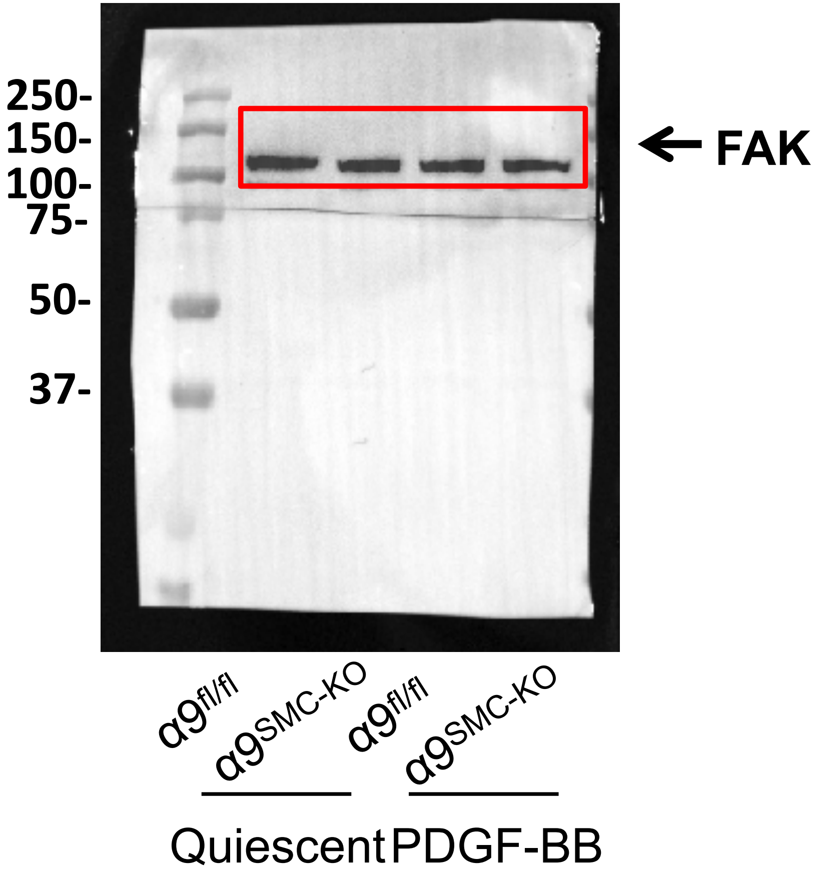

Full unedited gel for Figure 5A-p-Src

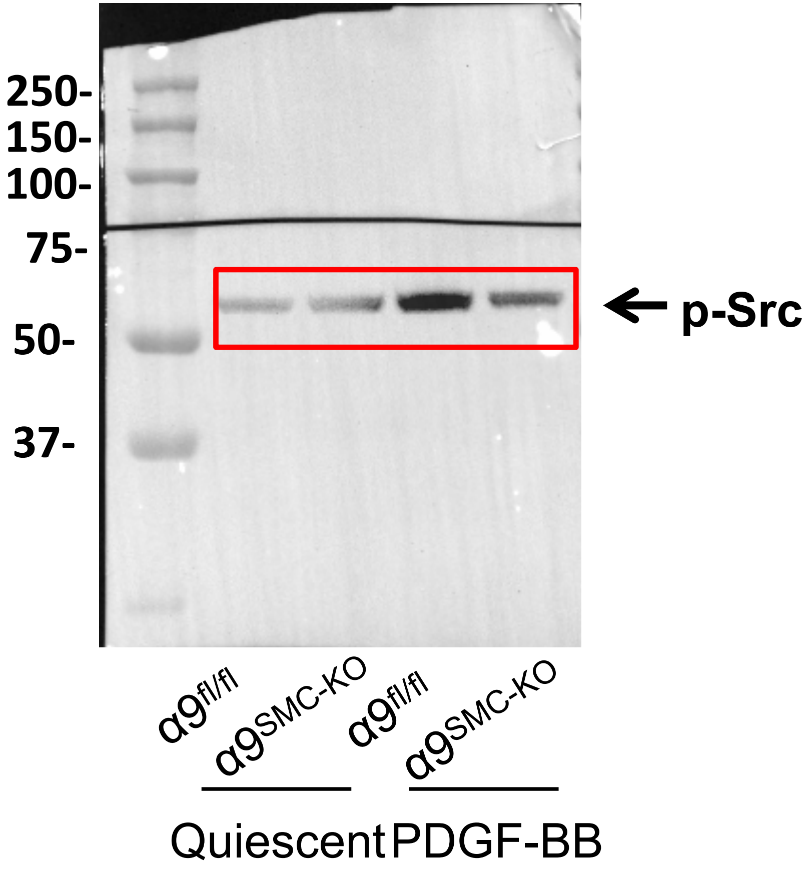

Full unedited gel for Figure 5A-Src

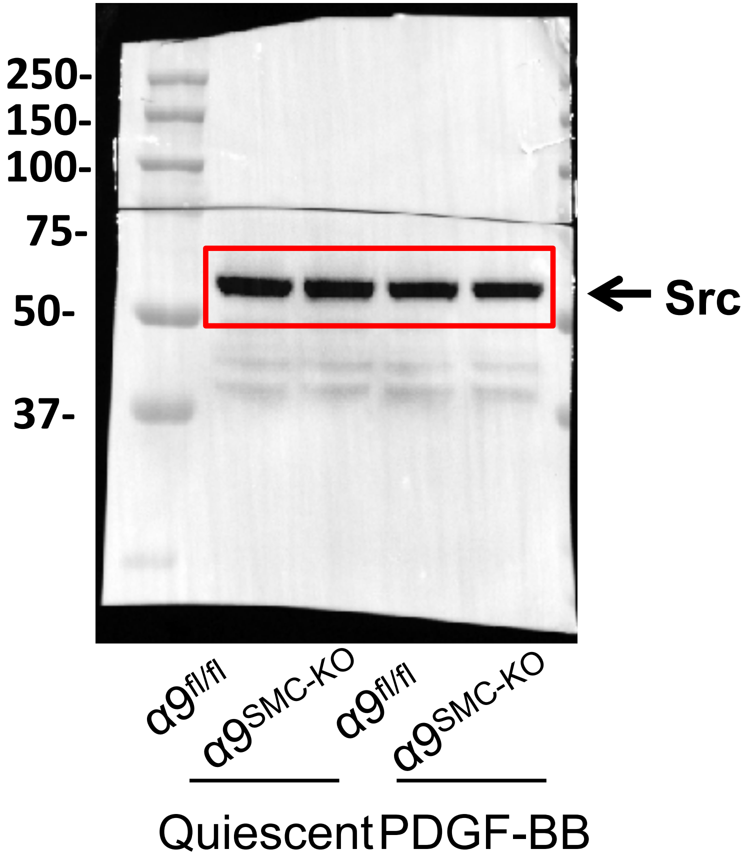

Full unedited gel for Figure 5A-pERK1/2

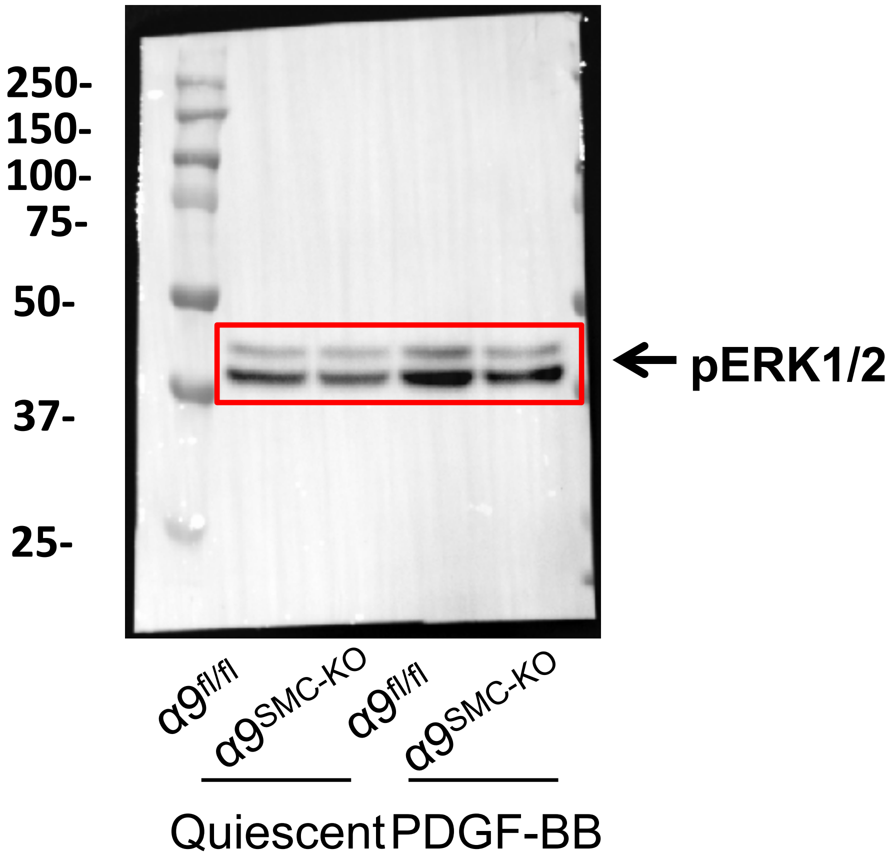

Full unedited gel for Figure 5A-ERK1/2

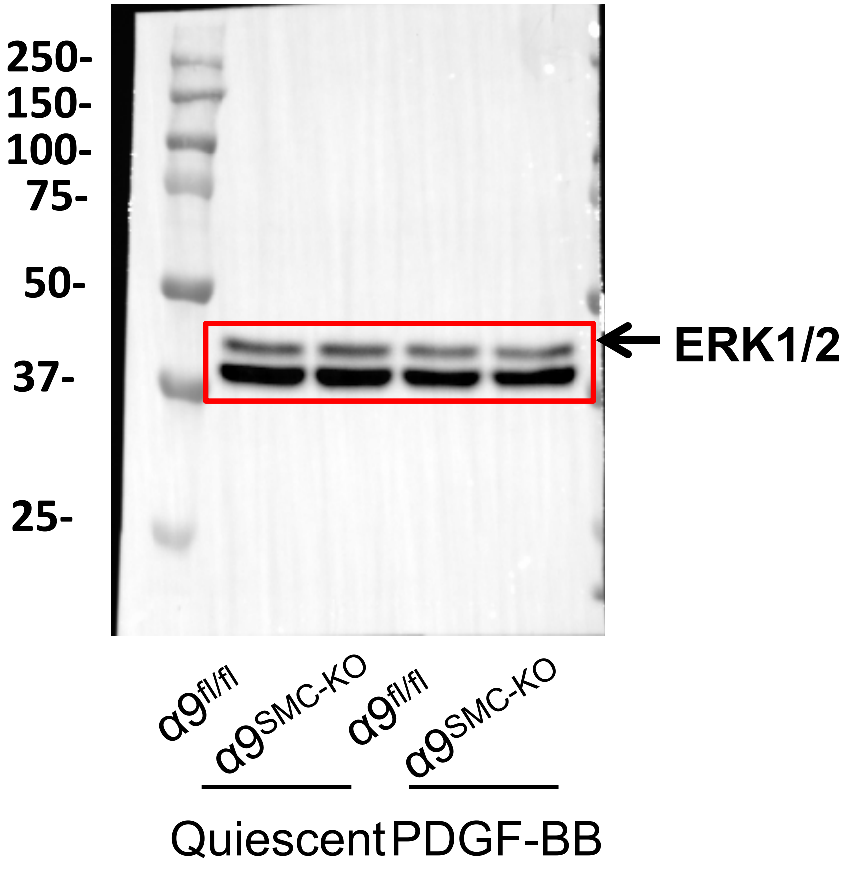

Full unedited gel for Figure 5A-p-p38

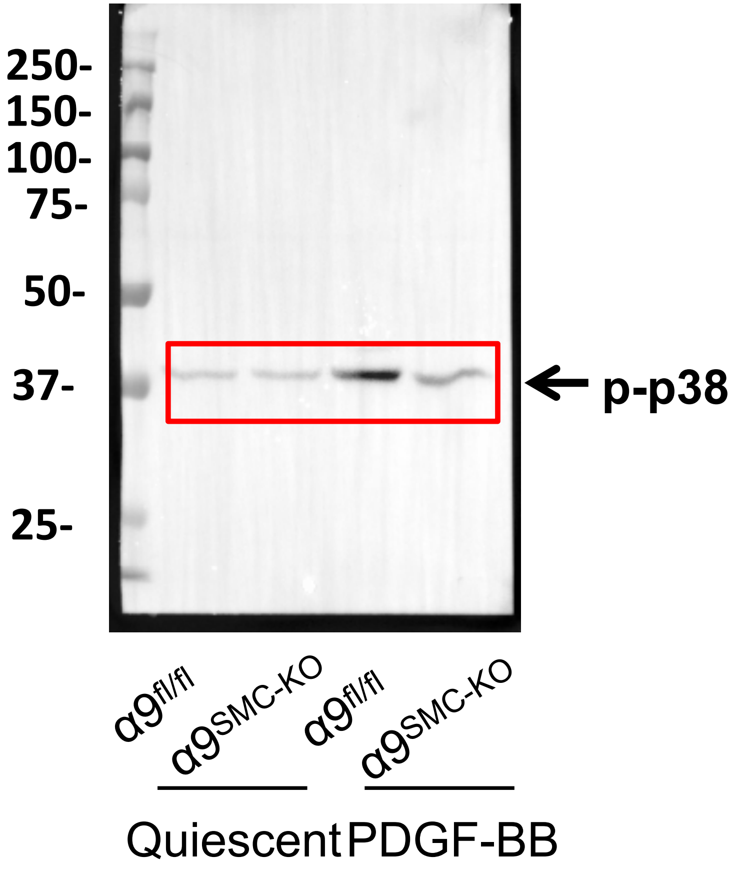

Full unedited gel for Figure 5A-p38

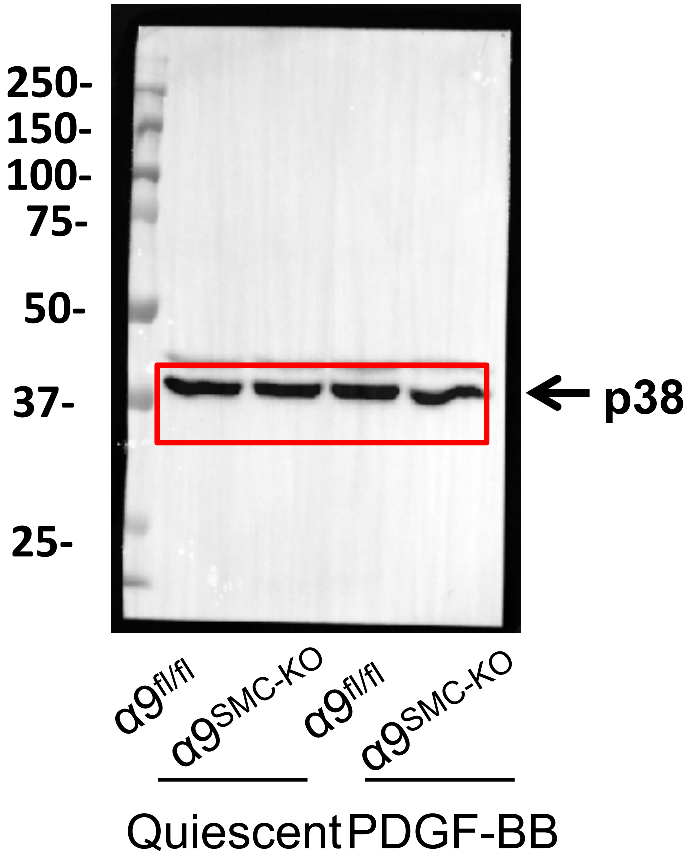

Full unedited gel for Figure 5B-pGSK3β

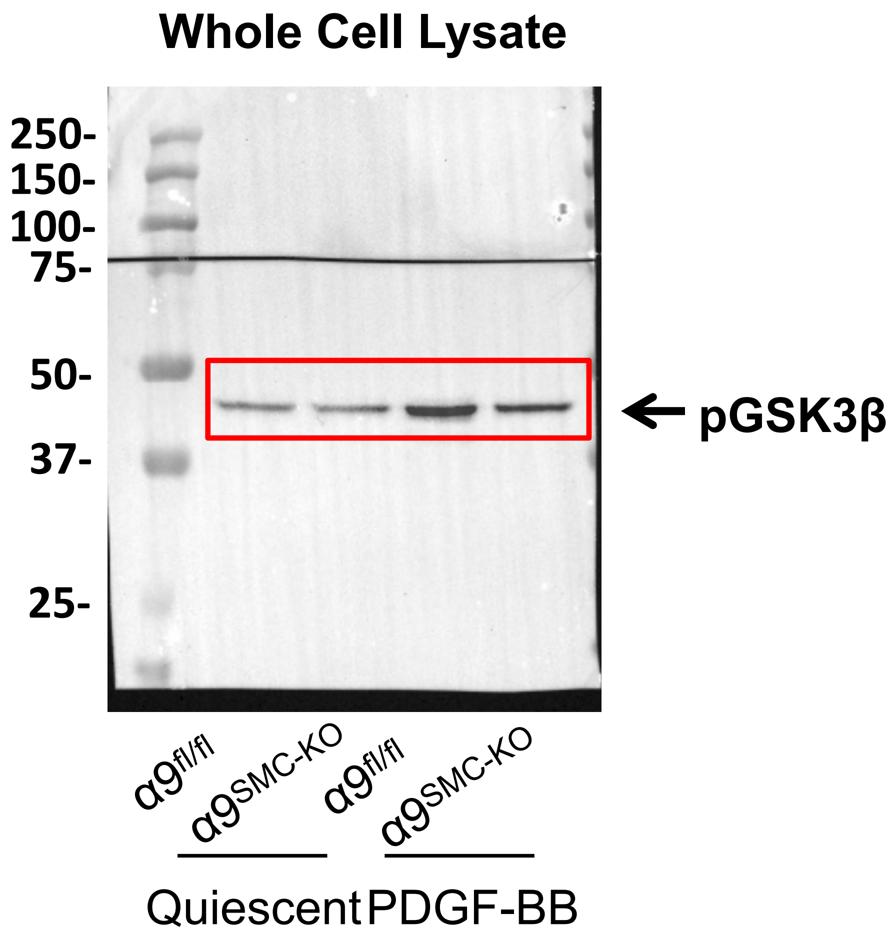

Full unedited gel for Figure 5B-GSK3β

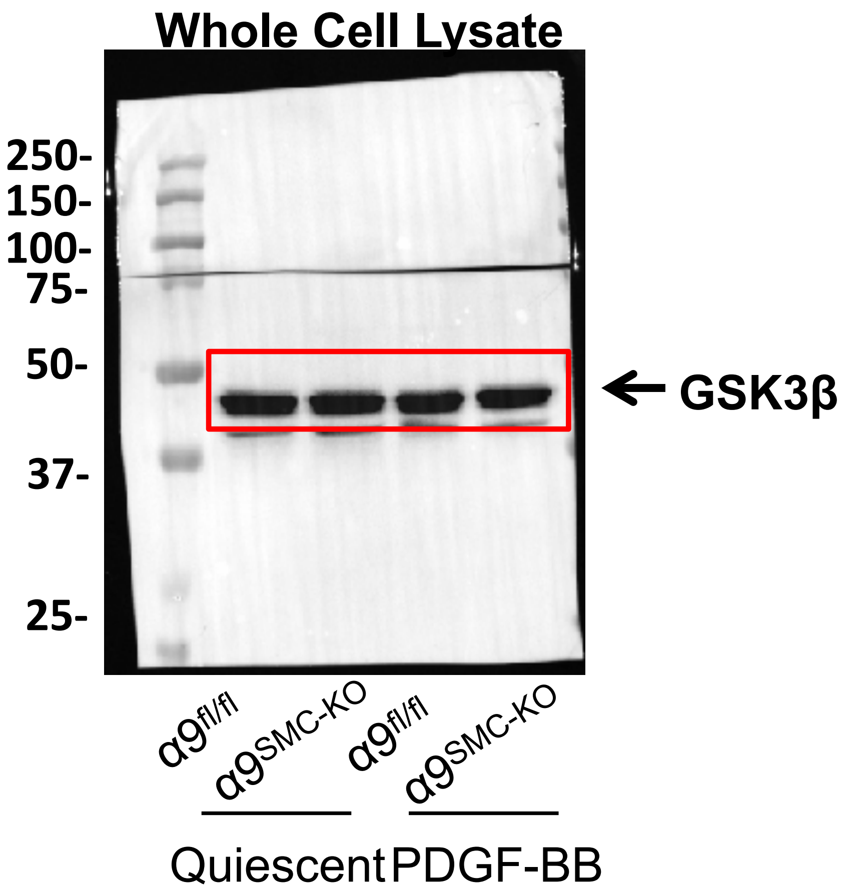

Full unedited gel for Figure 5B-Active β-catenin

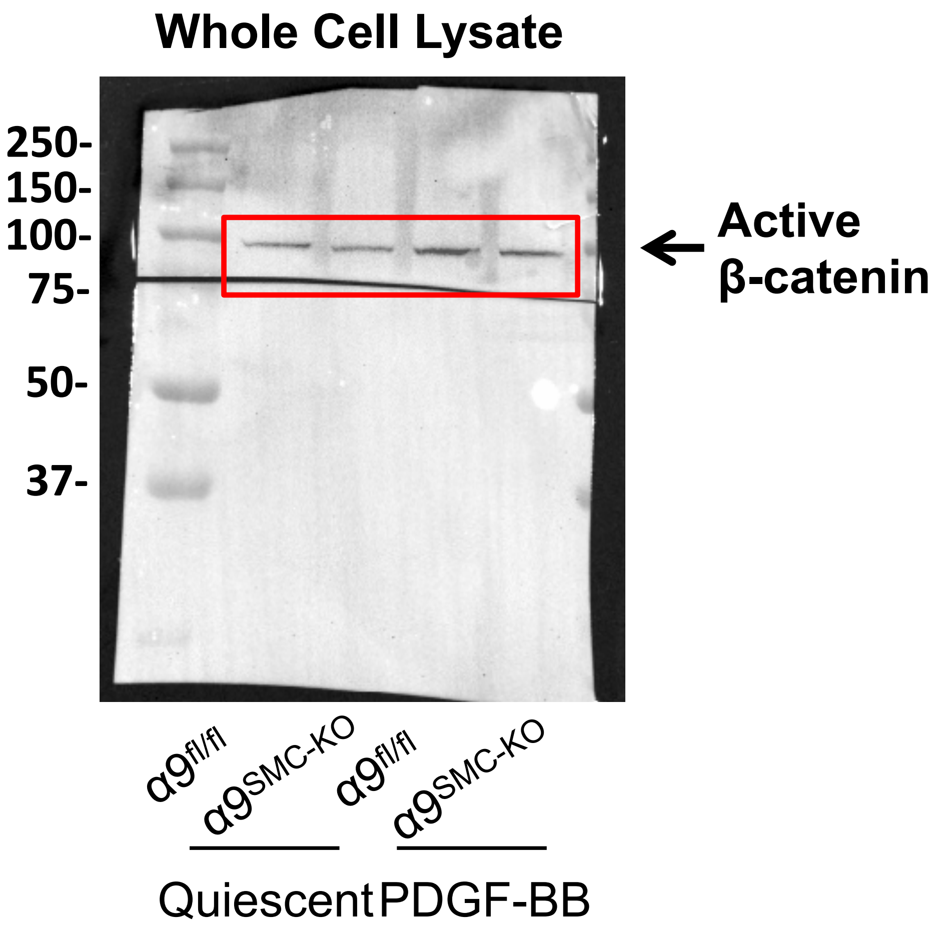

Full unedited gel for Figure 5B-Total β-catenin

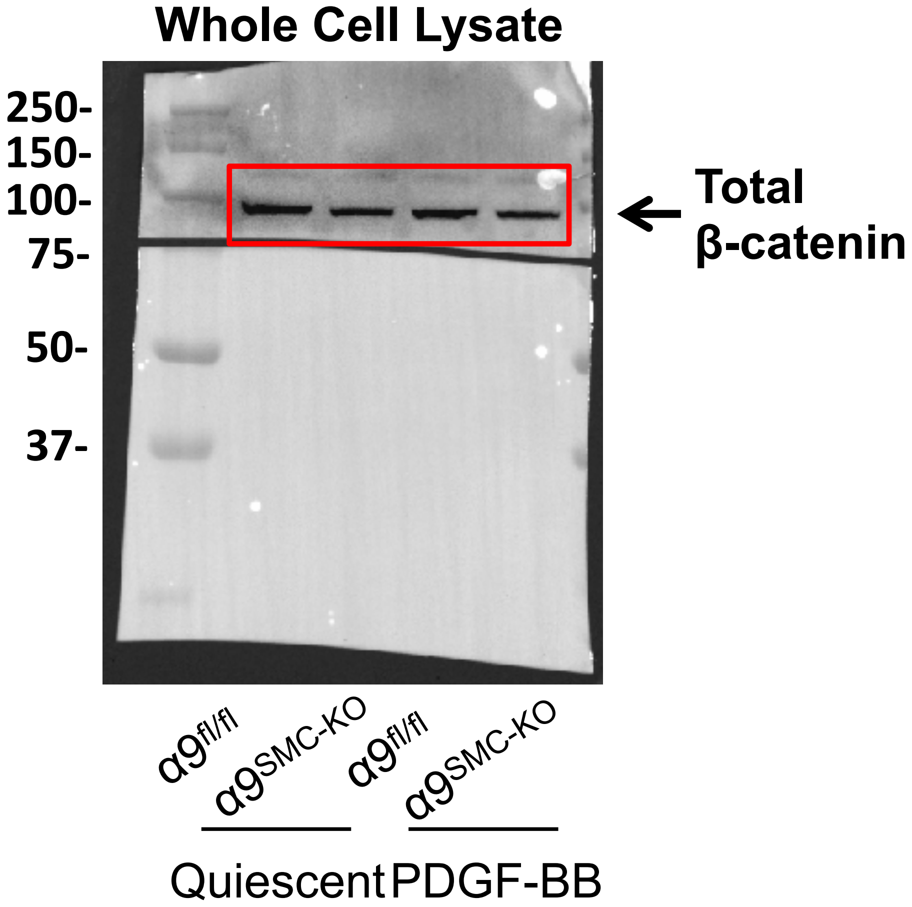

Full unedited gel for Figure 5B-β-Actin

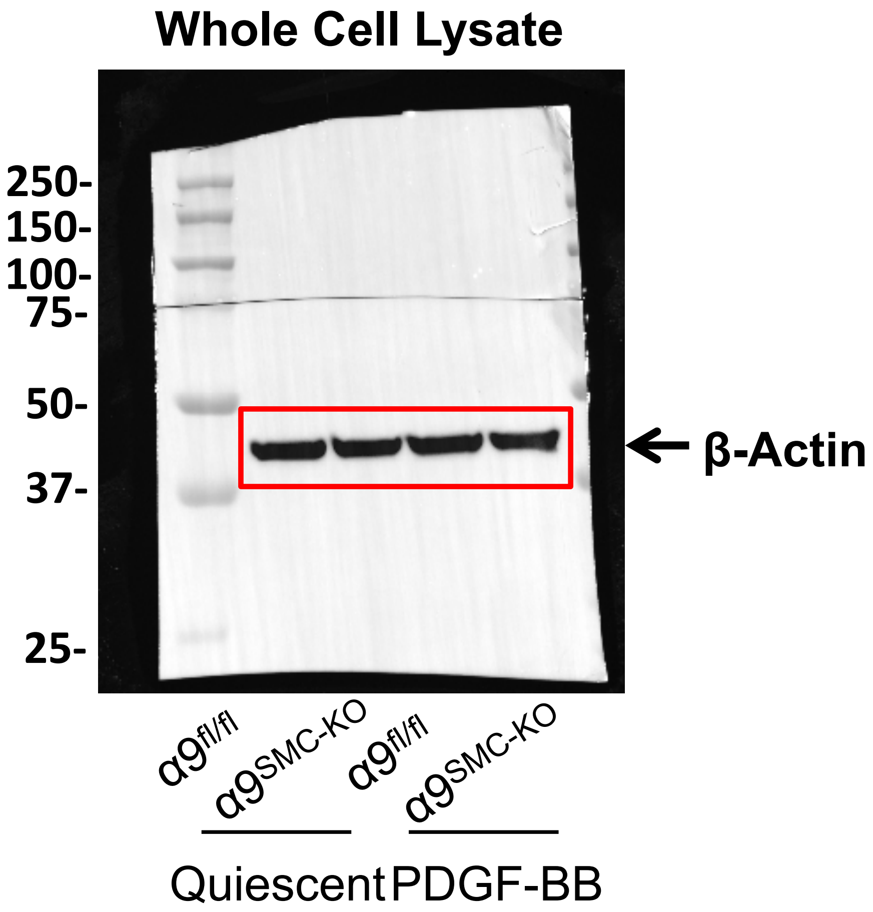

Full unedited gel for Figure 5C-β-catenin

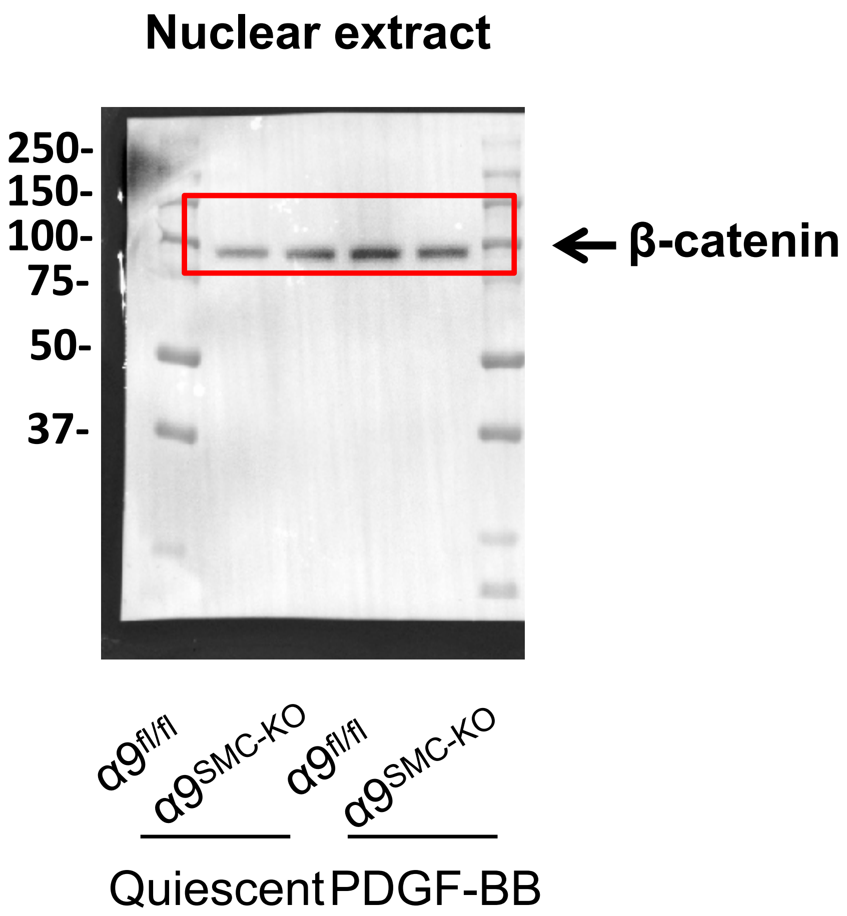

Full unedited gel for Figure 5C-Lamin B1

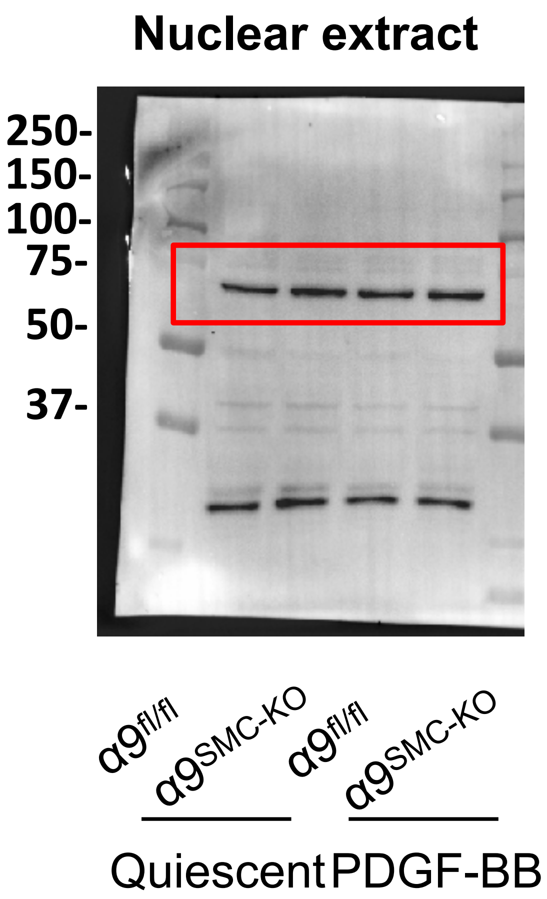

Full unedited gel for Figure 5C-GAPDH

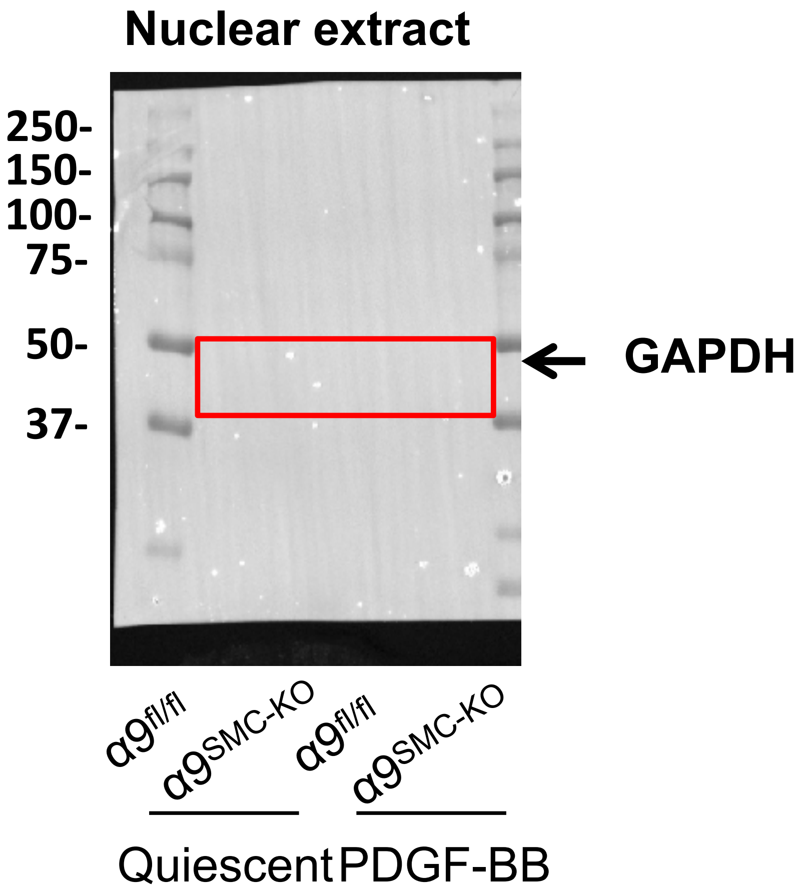

Full unedited gel for Figure 6C- p-FAK

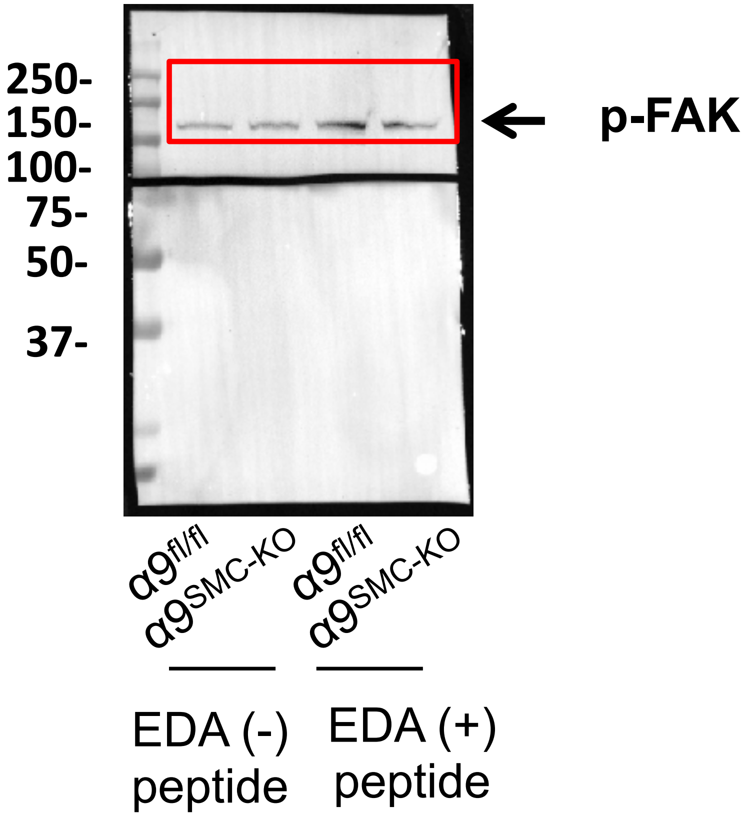

Full unedited gel for Figure 6C- FAK

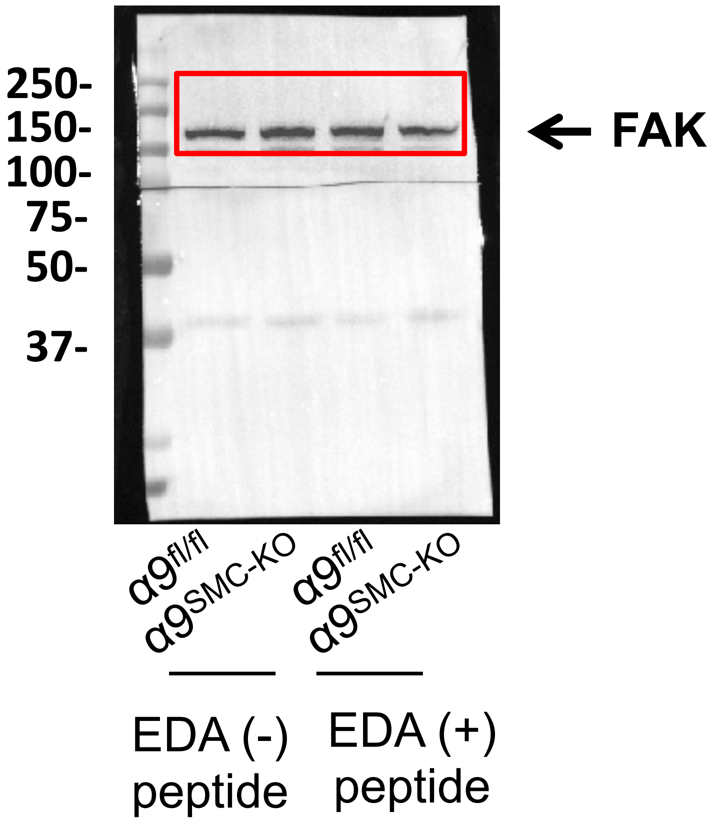

Full unedited gel for Figure 6C- p-ERK1/2

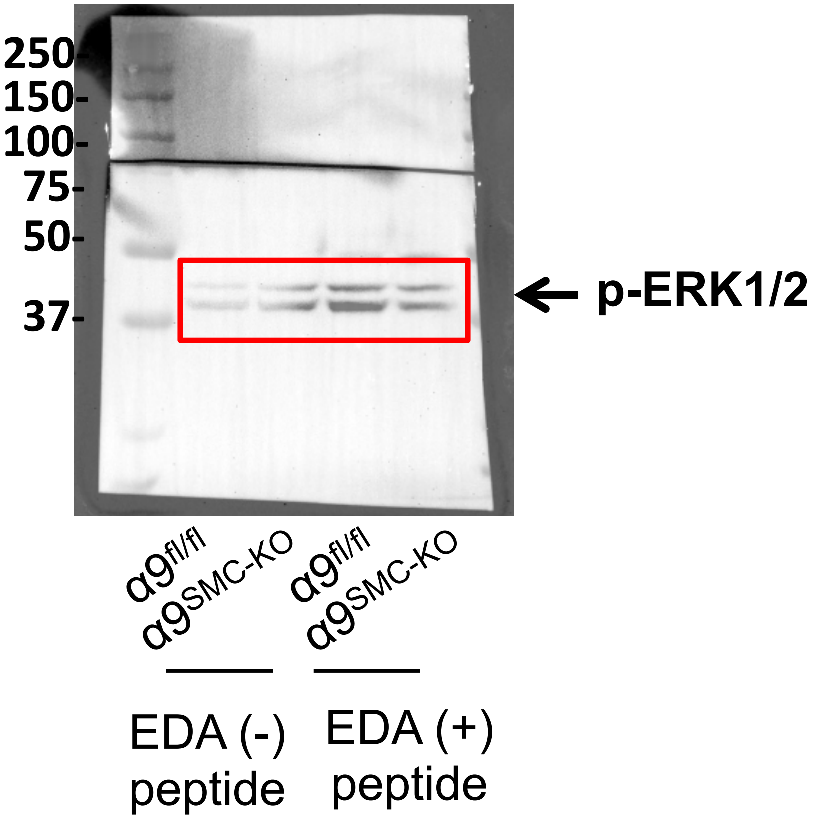

Full unedited gel for Figure 6C- ERK1/2

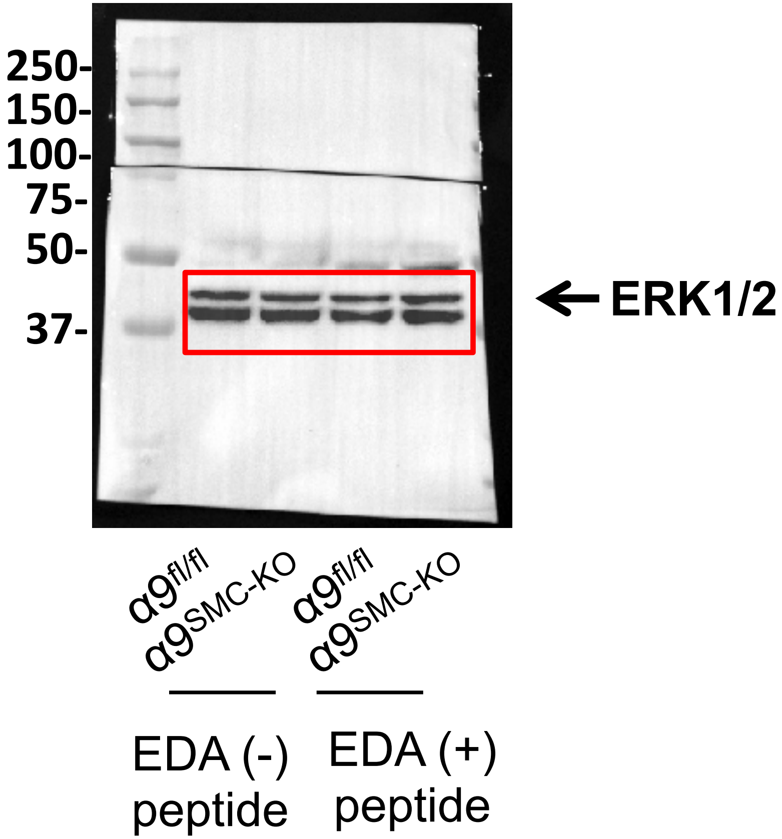

Full unedited gel for Figure 6C- p-p38

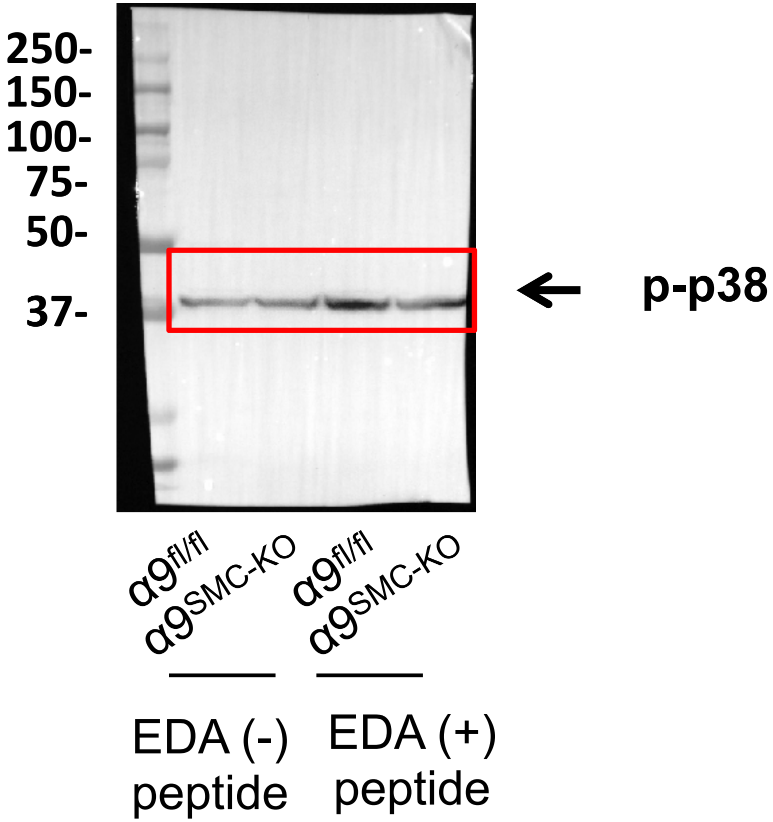

Full unedited gel for Figure 6C- p38

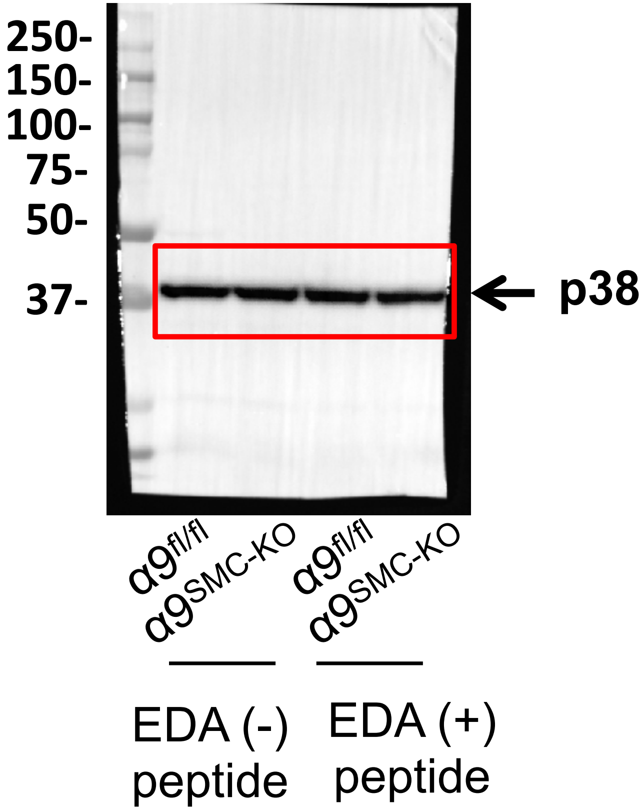

Full unedited gel for Figure 6C- β-catenin

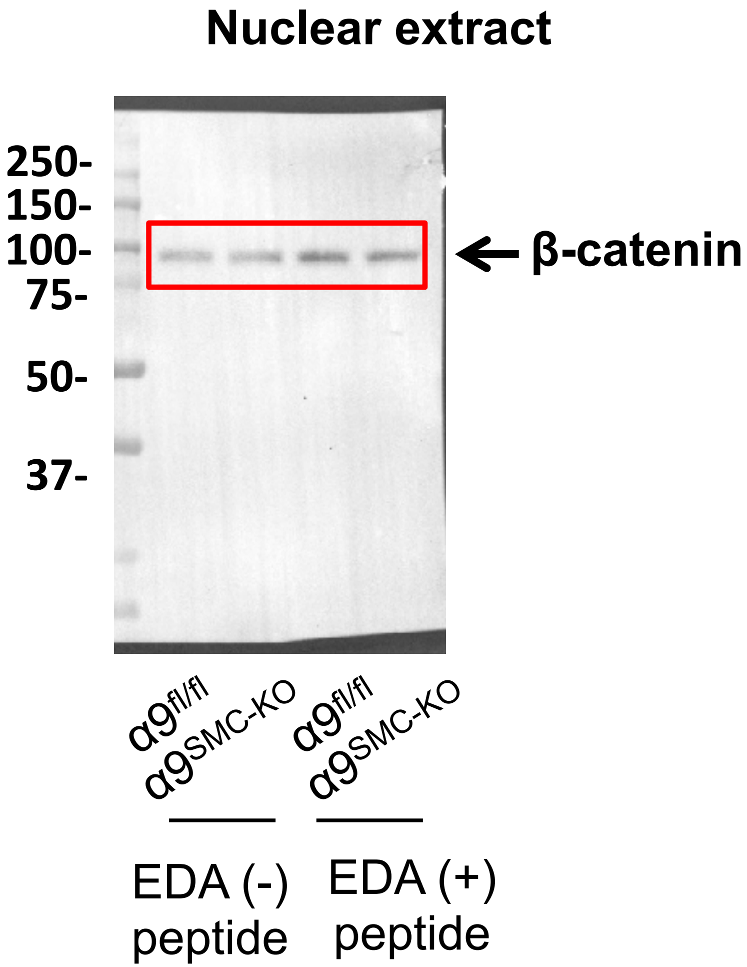

Full unedited gel for Figure 6C- Lamin B1

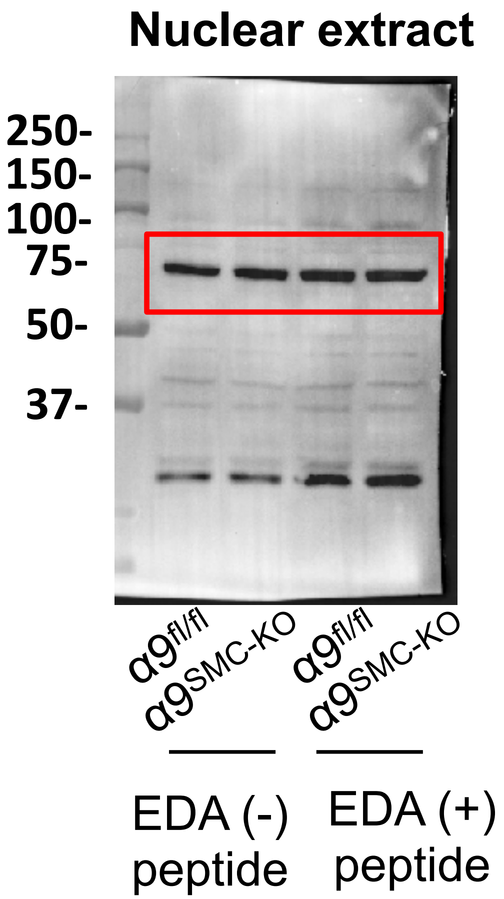

Full unedited gel for Figure 6C- GAPDH

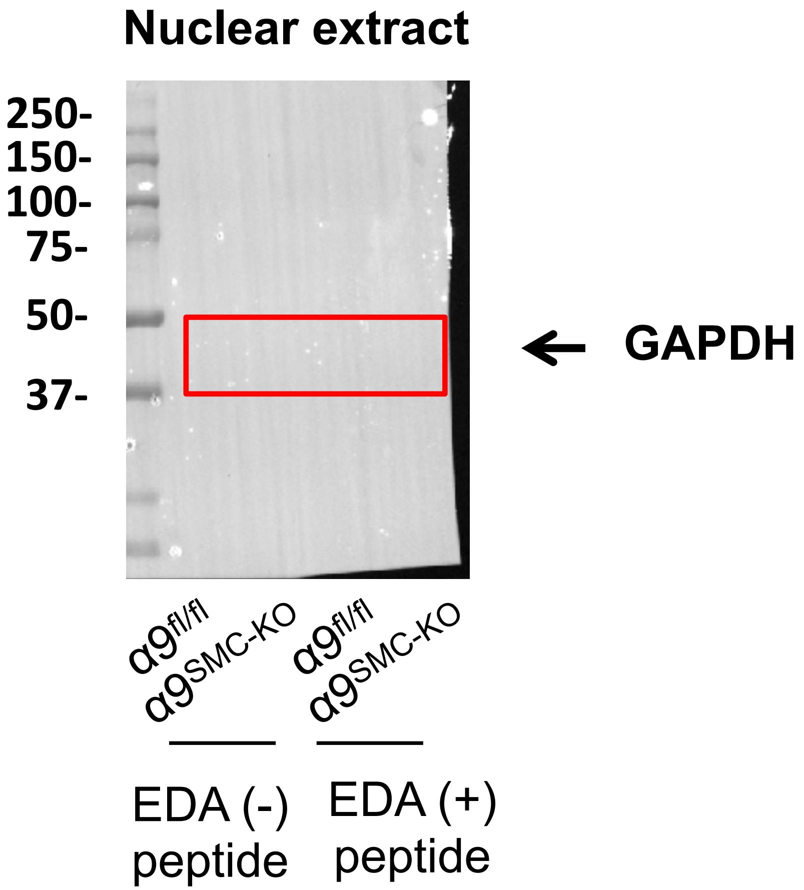

Full unedited gel for Supplementary Figure 3 –Integrin α9

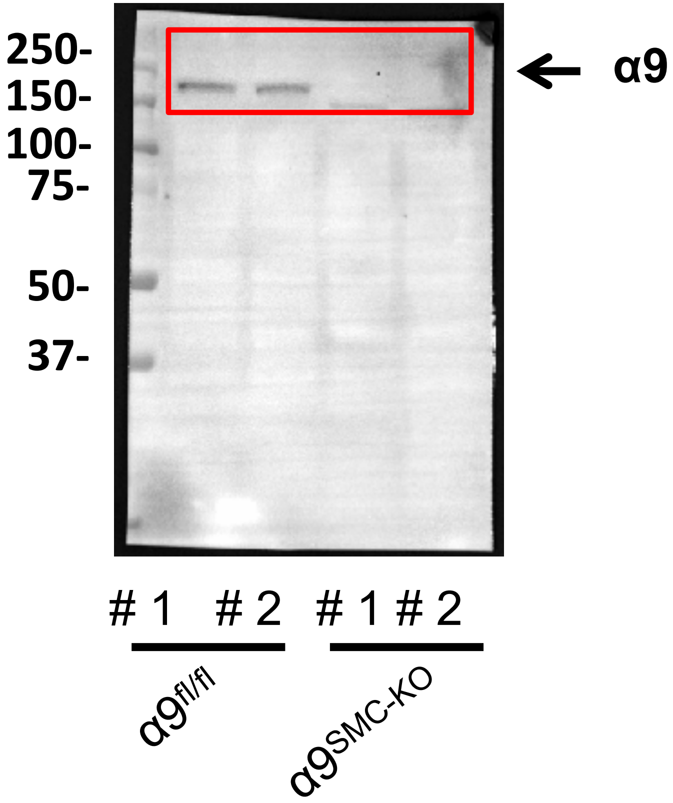

Full unedited gel for Supplementary Figure 3- Integrin β1

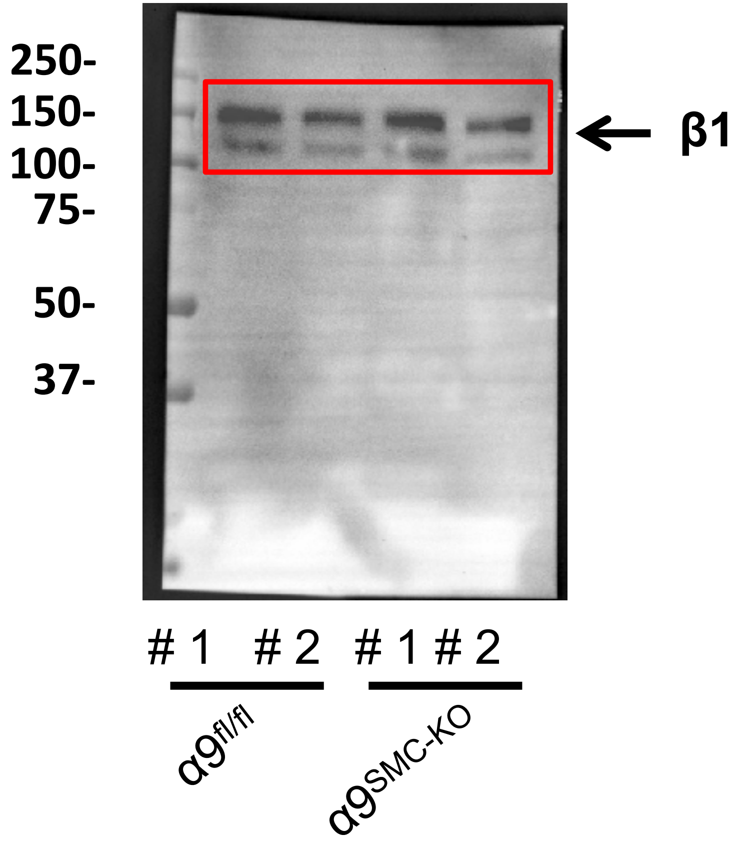

Full unedited gel for Supplementary Figure 3- β-Actin

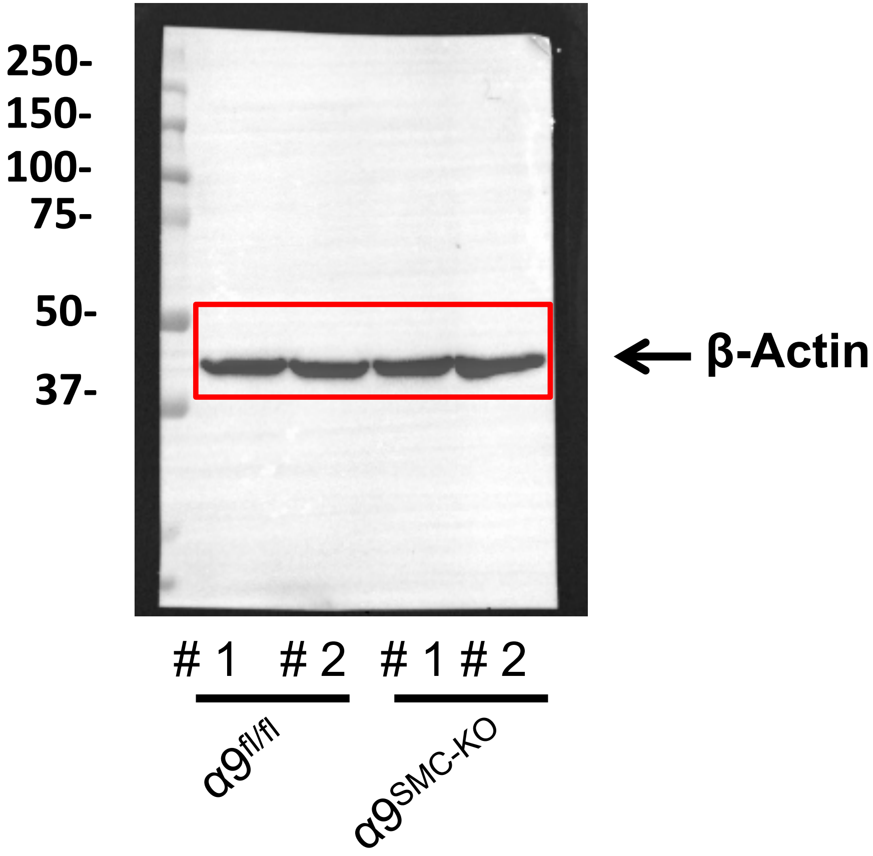

Full unedited gel for Supplementary Figure 6- β-catenin

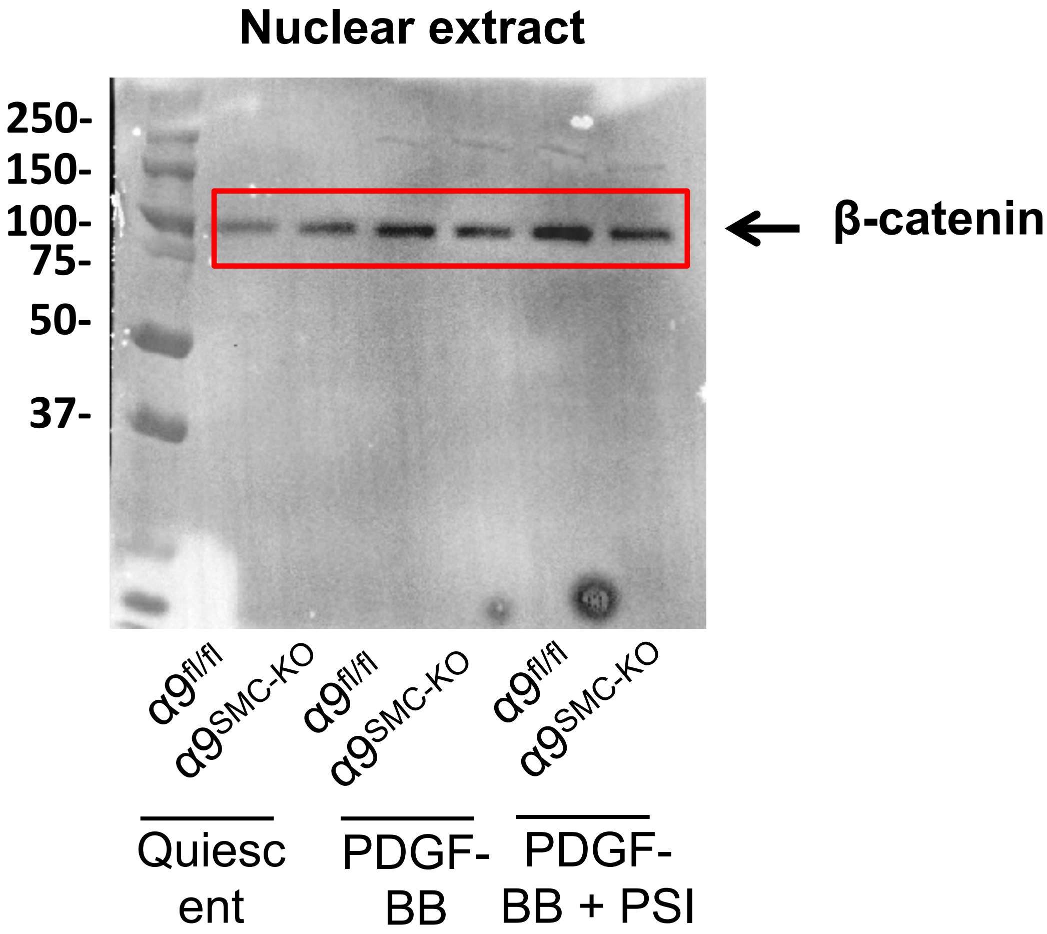

Full unedited gel for Supplementary Figure 6 -Lamin B1

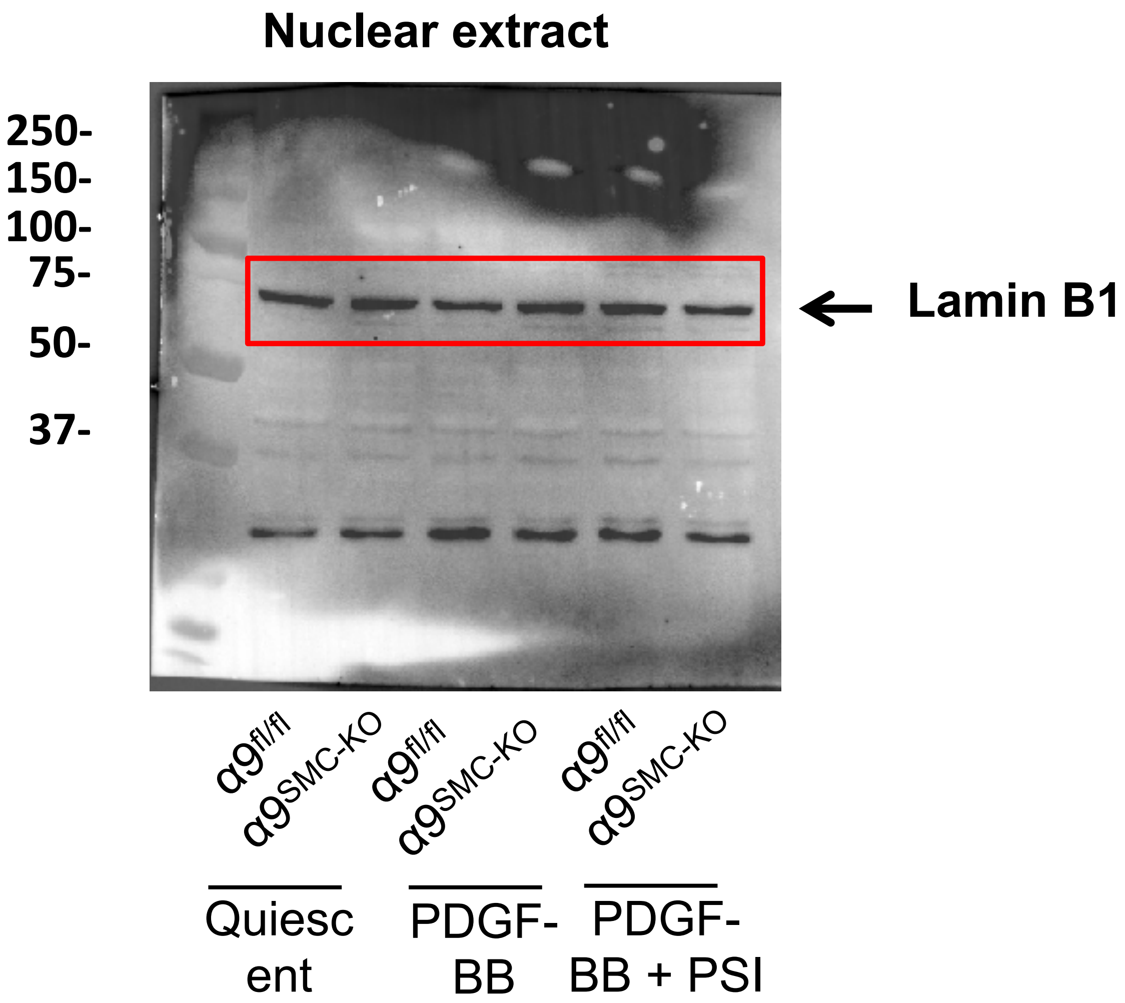

Full unedited gel for Supplementary Figure 6 -GAPDH

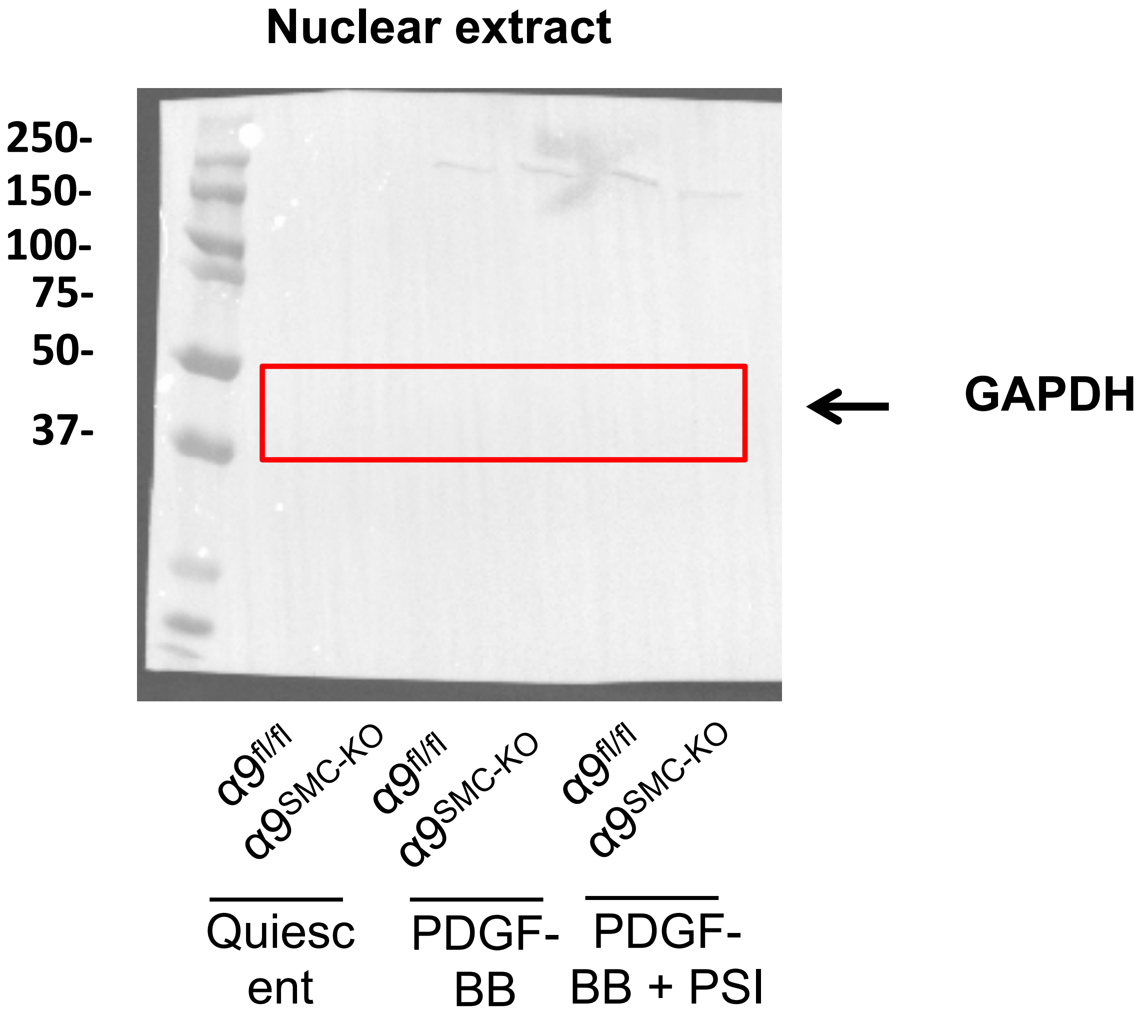

Full unedited gel for Supplementary Figure 6- β-Actin

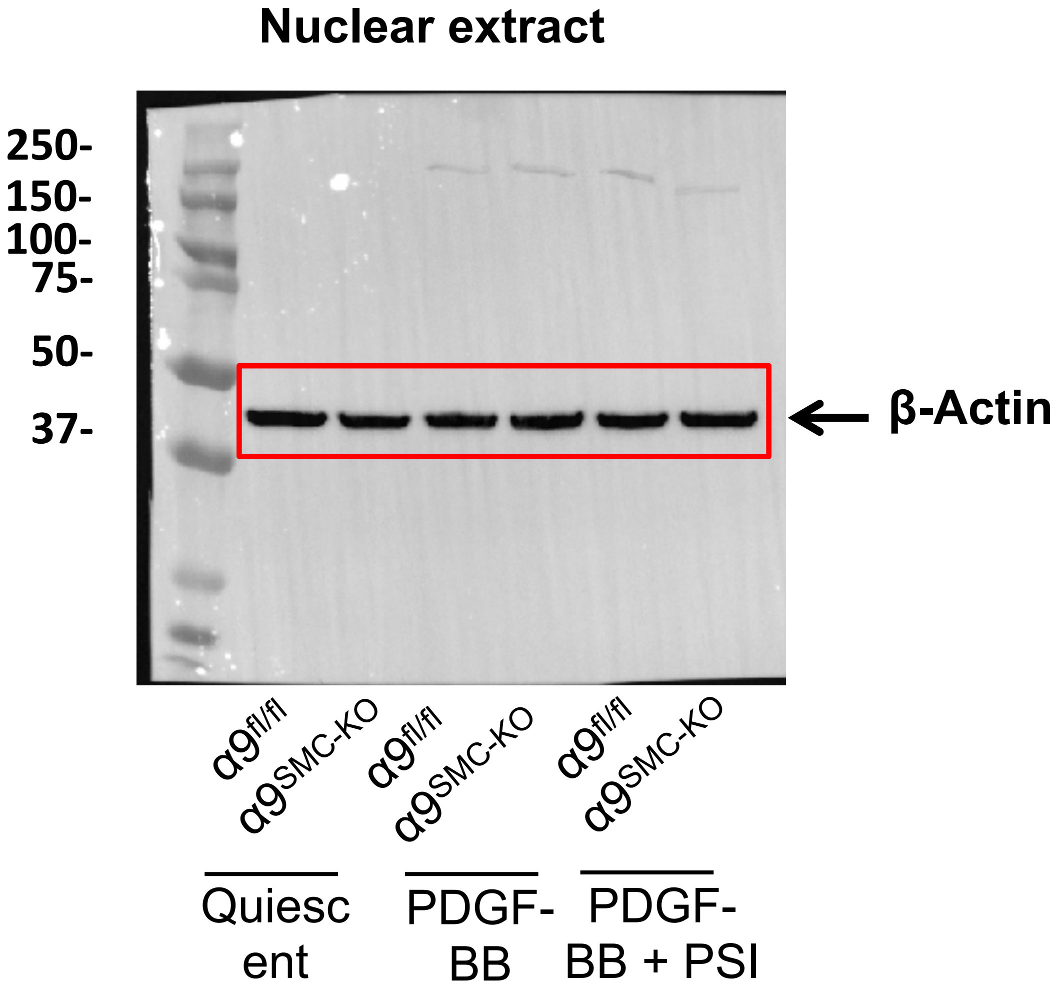

Supplement: Supplemental data [file jciinsight-6-147134-s081.pdf]
